# Supplementary material for: 5-HT regulates resistance to aumolertinib by attenuating ferroptosis in lung adenocarcinoma
Source: EMBO Mol Med. 2025 Sep 2;17(10):2586–611. doi: 10.1038/s44321-025-00293-5 (PMC12514003; doi:10.1038/s44321-025-00293-5)
Supplement: Supplementary file 1 — Appendix [file 44321_2025_293_MOESM1_ESM.pdf]

Appendix

5-HT regulates resistance to aumolertinib by attenuating ferroptosis in lung adenocarcinoma

Contents:

Appendix Figure

Appendix Figure S1. HER2 S310F mutation contributes to osimertinib resistance. (Page 2)

Appendix Figure S2. 5-HT contributes to osimertinib resistance. (Page 2)

Appendix Figure S3. MAOA expression is lower in various tumors compared to adjacent non-tumor tissues. (Page 3)

Appendix Figure S4. NRF2-mediated ferroptosis regulation. (Page 3)

Appendix Figure S5. the modulatory effects of 5-HT and palonosetron on core autophagy markers. (Page 4)

Appendix Figure S6. Histopathological analysis of murine cardiac, hepatic, and renal tissues. (Page 4)

Appendix Figure S7. Patient derived organoid validation of the effect of 5-HT/palonosetron on aumolertinib sensitivity. (Page 5)

Appendix Table

Appendix Table S1. FDA drug library screening for effective drugs in H1975 HER2 S310F cells (Page 6)

Appendix Table S2. cMAP database predicts effective drugs in H1975 HER2 S310F cells based on differential genes (Page 7)

Appendix Table S3. The sequences of lentivirus or plasmids used in this study (Page 8)

Appendix Table S4. List of primers for qRT-PCR (Page 8)

Appendix Table S5. List of primers for ChIP-PCR (Page 8)

Appendix Figure S1

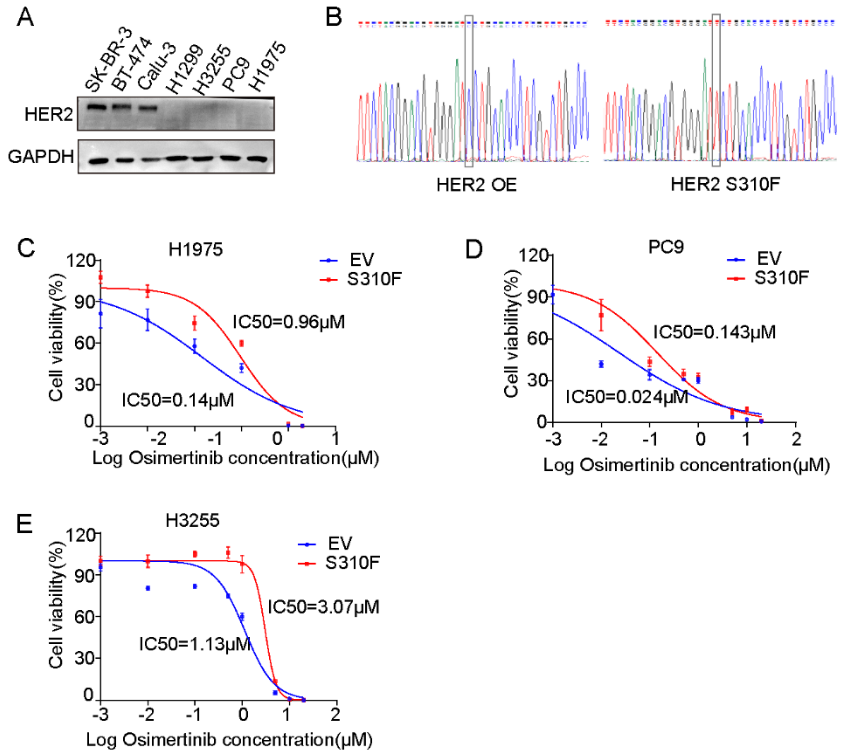

Appendix Figure S1. HER2 S310F mutation contributes to osimertinib resistance. A) The western blot analysis assesses the expression levels of HER2 across various cell lines. B) Validation of sequencing results for the HER2 S310F mutation. C-E) IC50 analysis of osimertinib by a CCK-8 assay in H1975 EV/S310F, PC9 EV/S310F and H3255 EV/S310F cells.

Appendix Figure S2

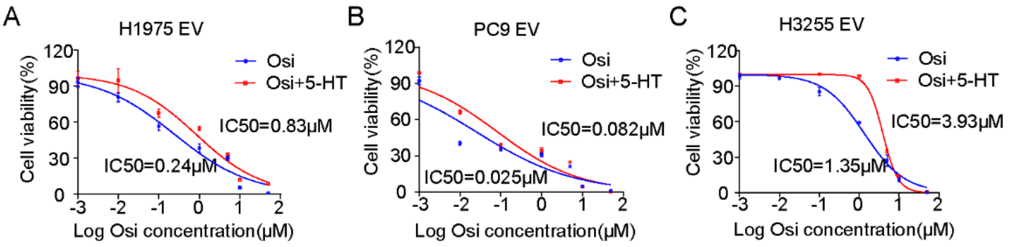

Appendix Figure S2 5-HT contributes to osimertinib resistance.A-C) Dose-response curves determined by the CCK-8 assay were used to calculate the IC50 values of osimertinib for 5 days in the presence or absence of 2.5 μM 5-HT.

Appendix Figure S3

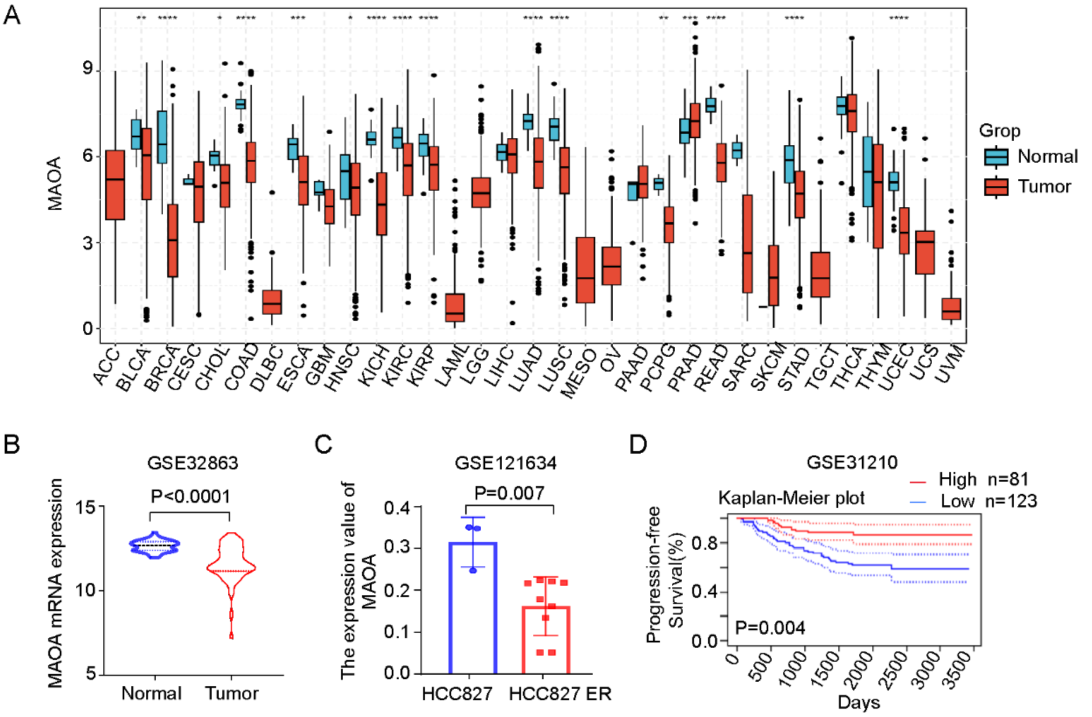

Appendix Figure S3. MAOA expression is lower in various tumors compared to adjacent non-tumor tissues. A) Analysis of MAOA expression in various tumors and adjacent normal tissues using the TCGA database. B) Analysis of MAOA expression in LUAD and adjacent normal tissues using the GSE32863 dataset. C) Analysis of MAOA expression in HCC827 wild-type and erlotinib-resistant cells using the GSE121634 dataset (HCC827, n = 3; HCC827 ER, n = 9). D) The Kaplan-Meier curve by PrognoScan shows significant PFS rate differences between the two kinds of pyroptosis phenotypes in the GSE31210 database. Data are presented as mean ± SD. Statistical test: Two-tailed unpaired Student's t test.

Appendix Figure S4

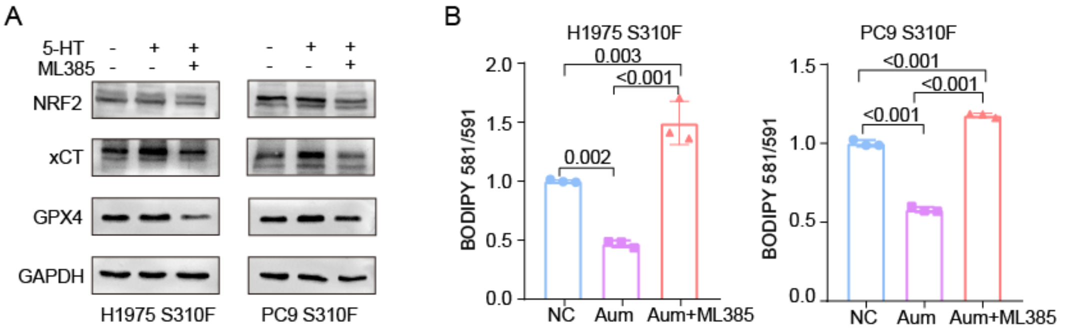

Appendix Figure S4 NRF2-mediated ferroptosis regulation. A) The protein levels of ferroptosis biomarkers after treatment with 5-HT alone with 5-HT alone or a combination of 5-HT and ML385 for 72 hours. B) The lipid ROS levels after treatment with aumolertinib alone or a combination of aumolertinib and ML385 in H1975 S310F and

PC9 S310F cells for 72 hours (n = 3). Data are presented as mean ± SD. Statistical test: one way ANOVA.

Appendix Figure S5

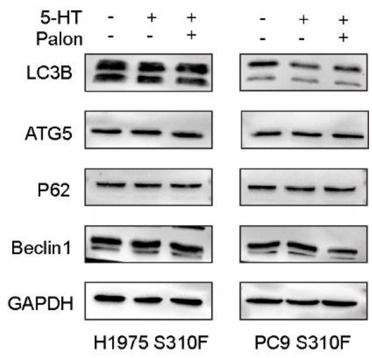

Appendix Figure S5 Western blotting analysis was performed to assess the modulatory effects of 5-HT and palonosetron on core autophagy markers.

Appendix Figure S6

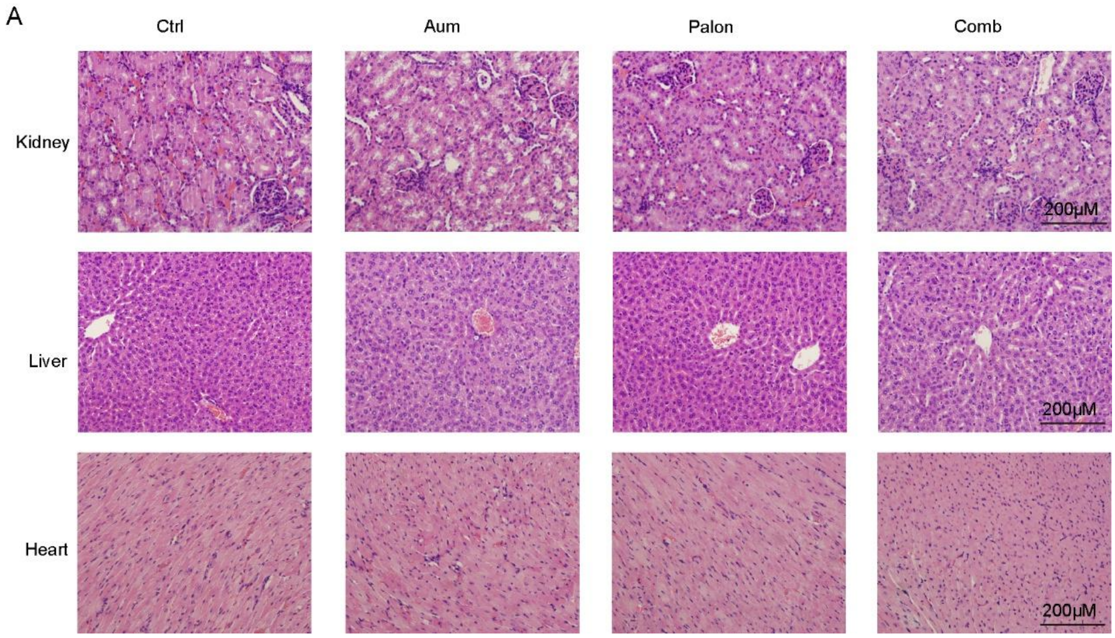

Appendix Figure S6 Histopathological analysis of murine cardiac, hepatic, and renal tissues following aumolertinib, palonosetron monotherapy or combination therapy.

Appendix Figure S7

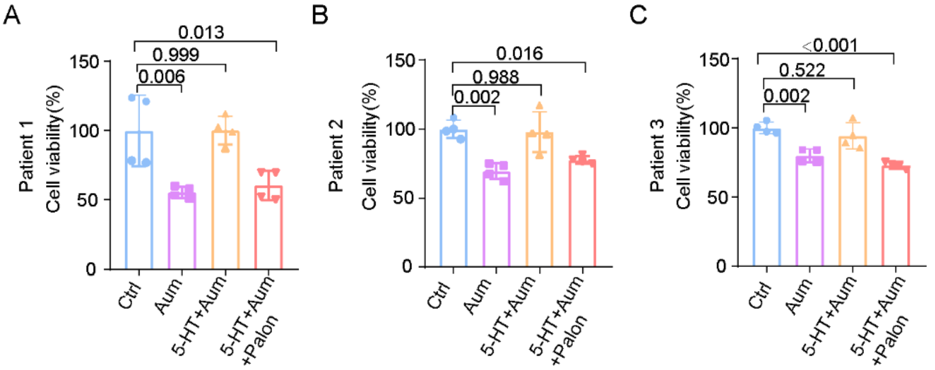

Appendix Figure S7 Patient derived organoid validation of the effect of 5-HT/palonosetron on aumolertinib sensitivity. A-C) Viability of lung adenocarcinoma organoids after treatment with aumolertinib, 5-HT + aumolertinib or a combination of 5-HT, aumolertinib and palonosetron. Data are presented as mean ± SD. Statistical test: one way ANOVA.

Appendix Table S1: FDA drug library screening for effective drugs in H1975 HER2 S310F cells

Agent	Target	Indication
Entinostat	HDAC	Cancer
Ondansetron HCl	HTR3	Antiemetic
Nisoldipine	Calcium channel	Cardiovascular Disease
Thioguanine	DNMT1	Cancer
Verteporfin	YAP	Angiology/Cancer
Chloroxine	Others	Infection/Inflammation
Ciclopirox	ATPase	Infection
Cepharanthine	TNF-alpha	Cancer/Inflammation
Methylene Blue	Others	Metabolic Disease
Nitroxoline	Topoisomerase	Infection
Tilorone dihydrochloride	Others	Infection
Nilotinib hydrochloride	Bcr-Abl	Cancer
Dronedarone	Others	Cardiovascular Disease
Afatinib Dimaleate	EGFR/HER2	Cancer
Belotecan hydrochloride	Topoisomerase	Cancer
Ebselen	Others	Infection
Staurosporine	PKC	Cancer/Infection
Ixazomib citrate	Proteasome	Cancer
Crizotinib hydrochloride	c-Met, Alk	Cancer
Cinacalcet	Calcium Channel	Endocrinology
Mometasone Furoate Hydrate	Others	Respiratory Disease
Palbociclib HCl	CDK	Cancer
Solifenacin succinate	AChR	Antispasmodic
Miltefosine	PI3K/Akt	Parasite
Cangrelor Tetrasodium	P2 Receptor	Cardiovascular Disease
Amifostine	Others	Cancer
Manganese chloride	Others	Metabolic Disease

Appendix Table S2: cMAP database predicts effective drugs in H1975 HER2 S310F cells based on differential genes

Score	Compound	Target
-98.31	U-0126	MEK inhibitor
-98.06	bisbenzimide	DNA binding agent
-97.29	BMS-536924	IGF-1 inhibitor
-97	lestaurtinib	FLT3 inhibitor
-94.75	saracatinib	SRC inhibitor
-91.91	VER-155008	HSP inhibitor
-91.56	fluticasone	Glucocorticoid receptor agonist
-90.78	TWS-119	Glycogen synthase kinase inhibitor
-90.75	SN-38	Topoisomerase inhibitor
-90.31	AS-605240	PI3K inhibitor
-89.97	dexamethasone	Glucocorticoid receptor agonist
-89.95	HG-5-113-01	Protein kinase inhibitor
-89.1	NVP-TAE684	ALK inhibitor
-88.78	LY-303511	Casein kinase inhibitor
-88.78	AS-703026	MEK inhibitor
-88.51	alosetron	HTR3
-87.83	teniposide	Topoisomerase inhibitor
-87.8	diflorasone	Corticosteroid agonist
-87.65	selumetinib	MEK inhibitor
-87.23	flubendazole	Tubulin inhibitor
-86.67	ethylestrenol	Progesterone receptor agonist
-86.56	QL-XI-92	DDR1 inhibitor
-85.32	neratinib	HER2 inhibitor

Appendix Table S3: The sequences of lentivirus or plasmids used in this study

Primer name	Primer sequence
HER2 S310F Forward	TGGGATCCTGCACCCTCGTCTGCCCC
HER2 S310F Reverse	GGGTGCAGAATCCCACGTCCGTAGAAAGGTAG
shHTR3A-1	CAAATATCCCGTACGTGTATA
shHTR3A-2	CTACAGCATCACCCTGGTTAT
shMAOA-1	AGCAGAGAGAAACCAGTTAAT
shMAOA-2	CCAGTCAAGTGACAACATCAT

Appendix Table S4: List of primers for qRT-PCR

Primer name	Primer sequence
Human TPH1 Forward	AACAAAGACCATTCCTCCGAAAG
Human TPH1 Reverse	TGTAACAGGCTCACATGATTCTC
Human MAOA Forward	GAATCAAGAGAAGGCGAGTATCG
Human MAOA Reverse	GGCAGCAGATAGTCCTGAAATG
Human L-AADC Forward	CCTACTGGCTGCTCGGACTAA
Human L-AADC Reverse	GCGTACCAGTGACTCAAACTC
Human SERT Forward	ACGGAGTTCTACAGAAGGTTGT
Human SERT Reverse	ATAGAGTGCCGTGTGTCATCT
Human VMAT1 Forward	GGTGGATTCTTCTATGATGCCC
Human VMAT1 Reverse	GTGGATGGACCTATAGCAAAGC
Human GAPDH Forward	TGAAGGTCGGAGTCAACGGATTTGGT
Human GAPDH Reverse	CATGTGGGCCATGAGGTCCACCAC

Appendix Table S5: List of primers for ChIP-PCR

Primer name	Primer sequence
MAOA-S1 Forward	CGGAGTTTCGCTCTTGTTGC
MAOA-S1 Reverse	GGAGGCTGAGGCAGGAAAAT
MAOA-S2 Forward	ACGTCTACTCCCCCACTCTC
MAOA-S2 Reverse	GGCGGGGAGAAGTGACTCTA
MAOA-S3 Forward	TTCTGCACCTTAGCCATCCC
MAOA-S3 Reverse	GGGTTTTTCAAGCTGAGGCG
