# Supplementary figures and images for: 5-HT regulates resistance to aumolertinib by attenuating ferroptosis in lung adenocarcinoma (part 1 of 2)
Source: EMBO Mol Med. 2025 Sep 2;17(10):2586–611. doi: 10.1038/s44321-025-00293-5 (PMC12514003; doi:10.1038/s44321-025-00293-5)

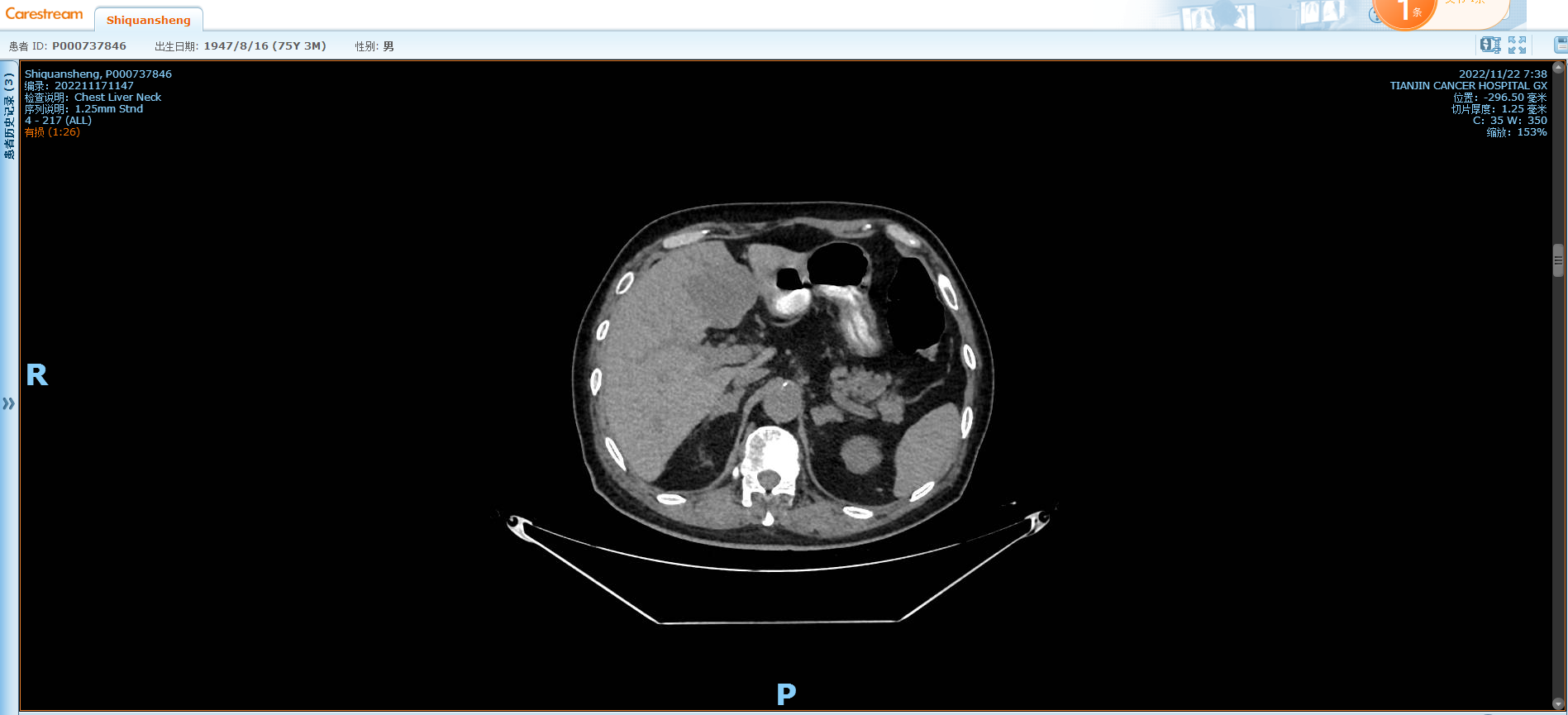

Supplement: Supplementary file 4 — Source data Fig. 1 [file 44321_2025_293_MOESM4_ESM.zip › Figure 1/1A/22.11CT.tif]

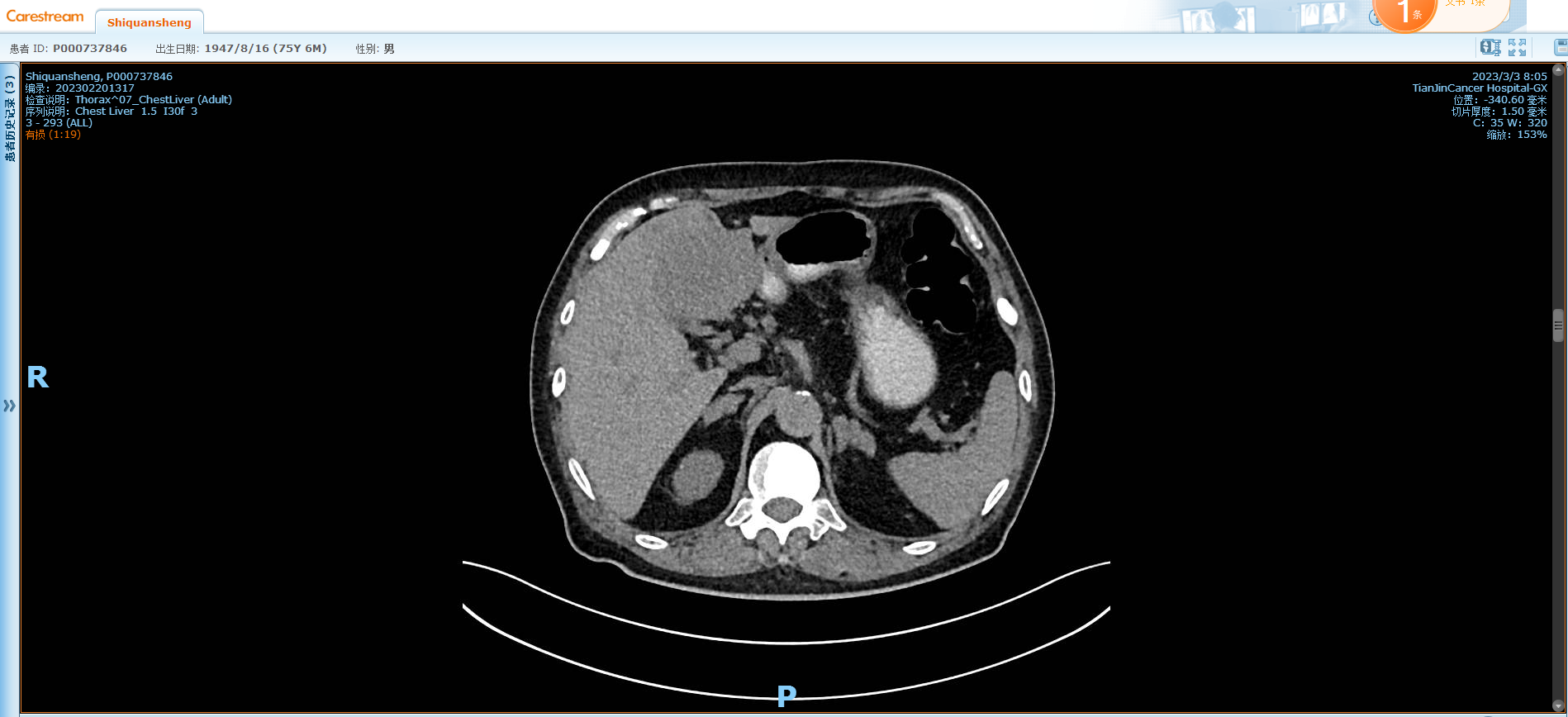

Supplement: Supplementary file 4 — Source data Fig. 1 [file 44321_2025_293_MOESM4_ESM.zip › Figure 1/1A/23.3CT.tif]

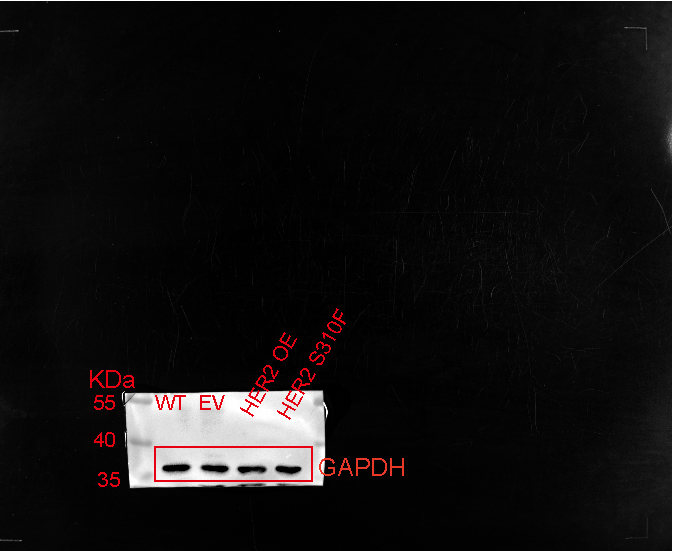

Supplement: Supplementary file 4 — Source data Fig. 1 [file 44321_2025_293_MOESM4_ESM.zip › Figure 1/1B/western H1975_GAPDH.tif]

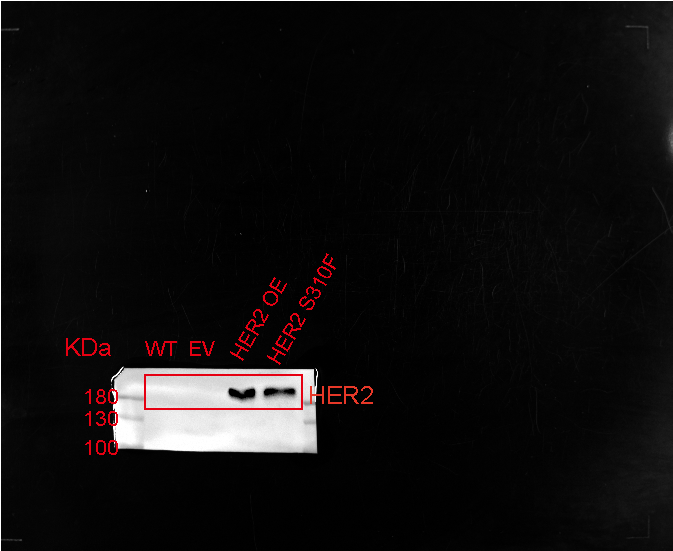

Supplement: Supplementary file 4 — Source data Fig. 1 [file 44321_2025_293_MOESM4_ESM.zip › Figure 1/1B/western H1975_HER2.tif]

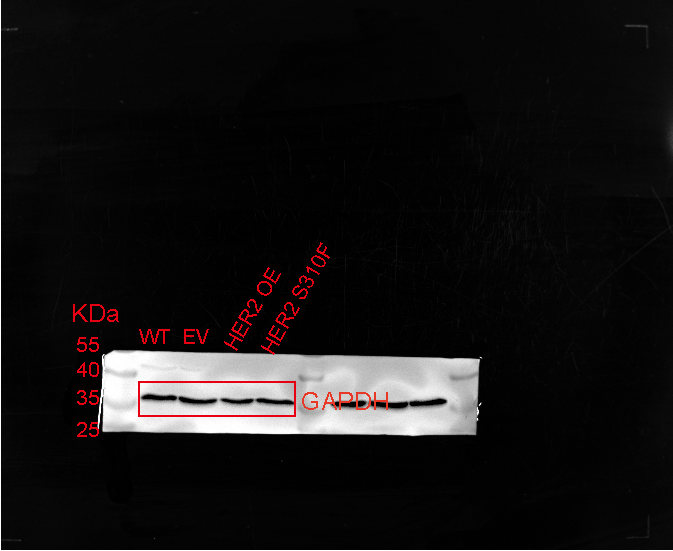

Supplement: Supplementary file 4 — Source data Fig. 1 [file 44321_2025_293_MOESM4_ESM.zip › Figure 1/1B/western H3255_GAPDH.tif]

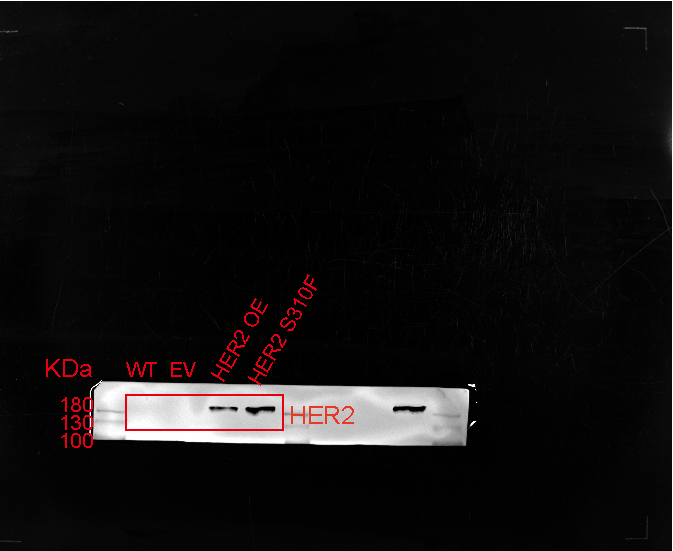

Supplement: Supplementary file 4 — Source data Fig. 1 [file 44321_2025_293_MOESM4_ESM.zip › Figure 1/1B/western H3255_HER2.tif]

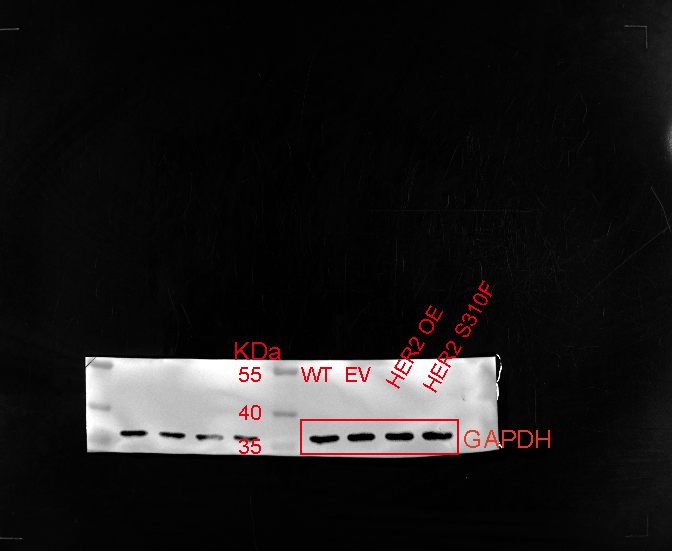

Supplement: Supplementary file 4 — Source data Fig. 1 [file 44321_2025_293_MOESM4_ESM.zip › Figure 1/1B/western PC9_GAPDH.tif]

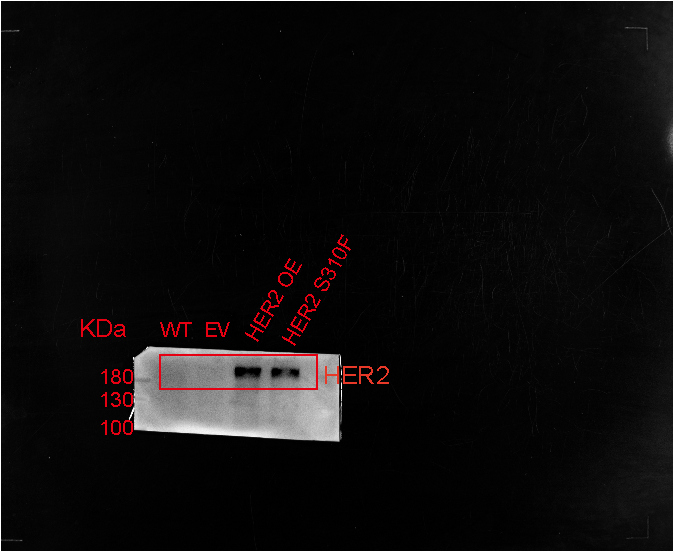

Supplement: Supplementary file 4 — Source data Fig. 1 [file 44321_2025_293_MOESM4_ESM.zip › Figure 1/1B/western PC9_HER2.tif]

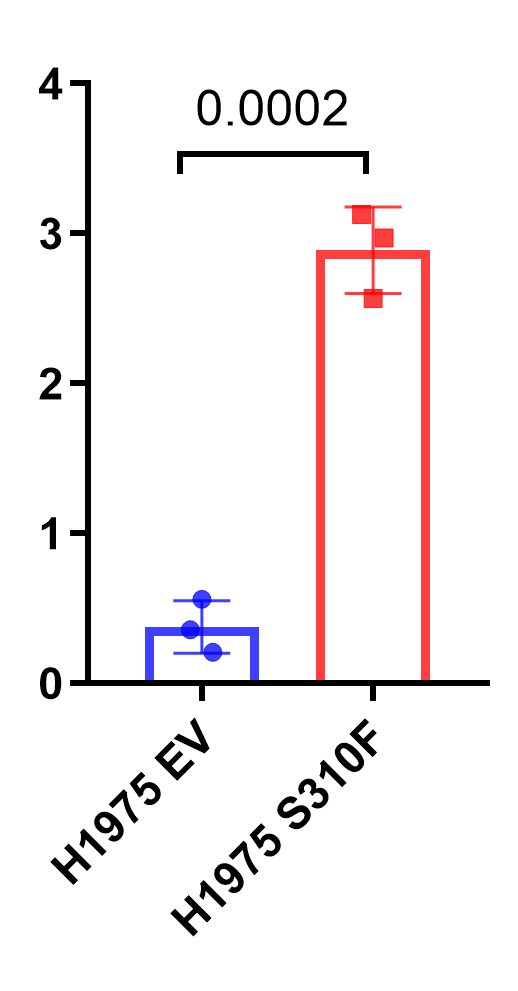

Supplement: Supplementary file 4 — Source data Fig. 1 [file 44321_2025_293_MOESM4_ESM.zip › Figure 1/1C/H1975 3 re.tif]

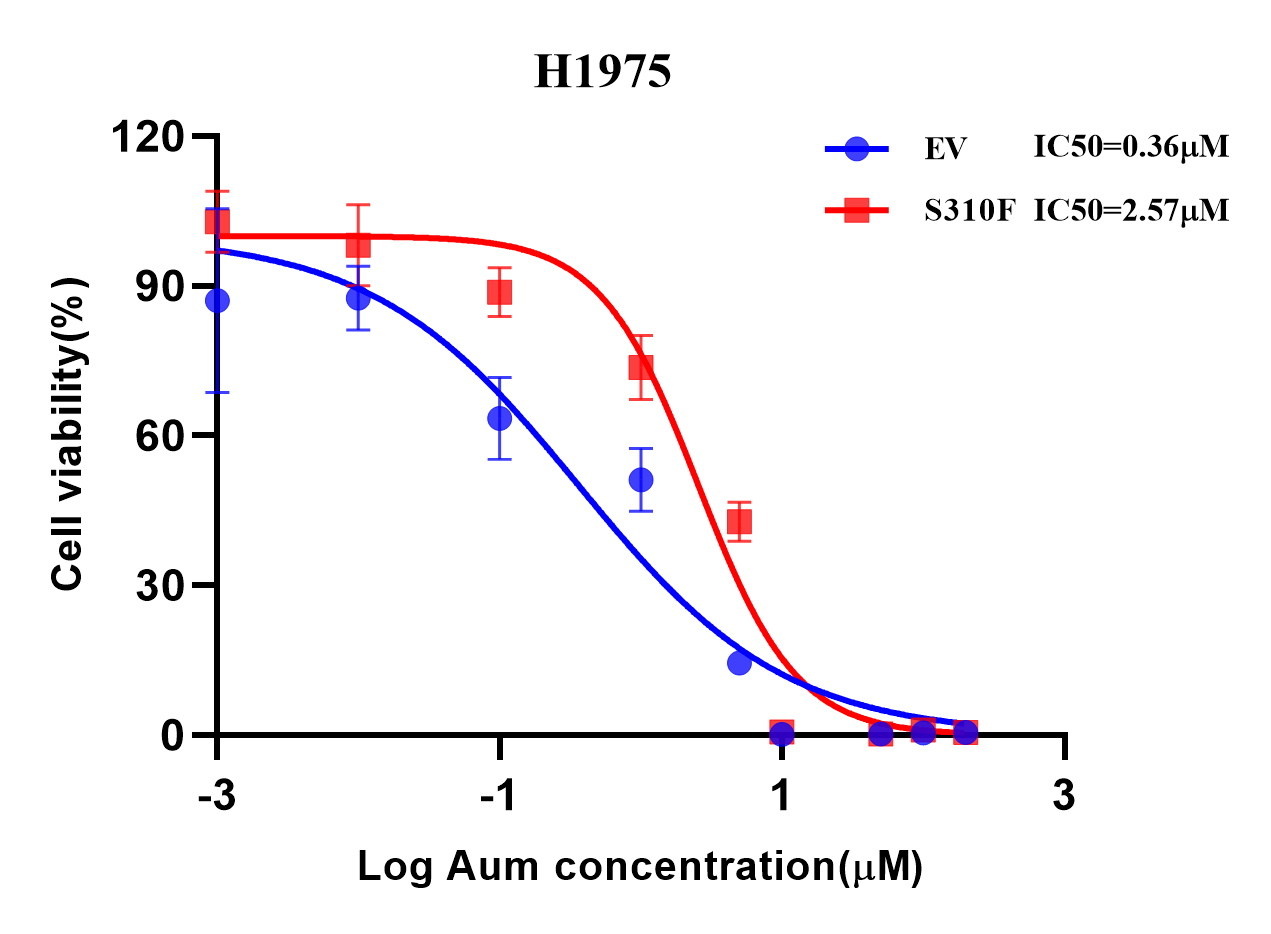

Supplement: Supplementary file 4 — Source data Fig. 1 [file 44321_2025_293_MOESM4_ESM.zip › Figure 1/1C/H1975 Aum IC50.tif]

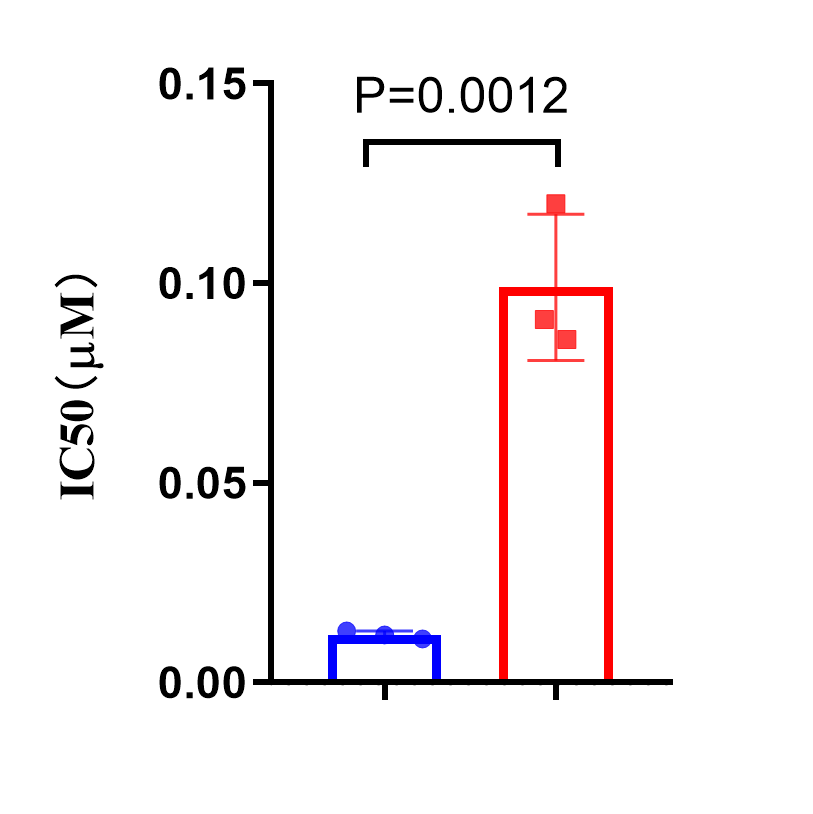

Supplement: Supplementary file 4 — Source data Fig. 1 [file 44321_2025_293_MOESM4_ESM.zip › Figure 1/1D/PC9 IC50 3re.tif]

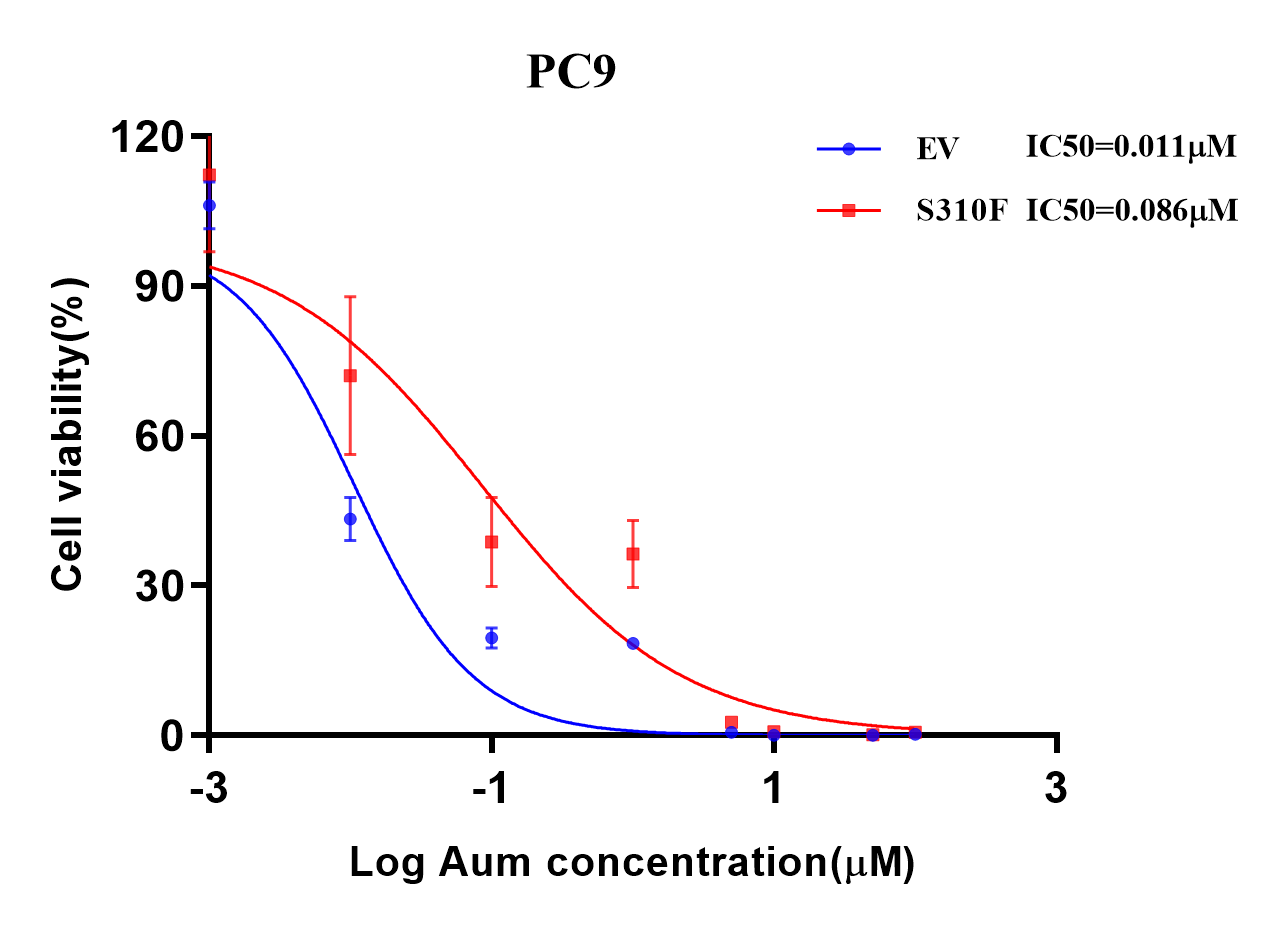

Supplement: Supplementary file 4 — Source data Fig. 1 [file 44321_2025_293_MOESM4_ESM.zip › Figure 1/1D/PC9 IC50.tif]

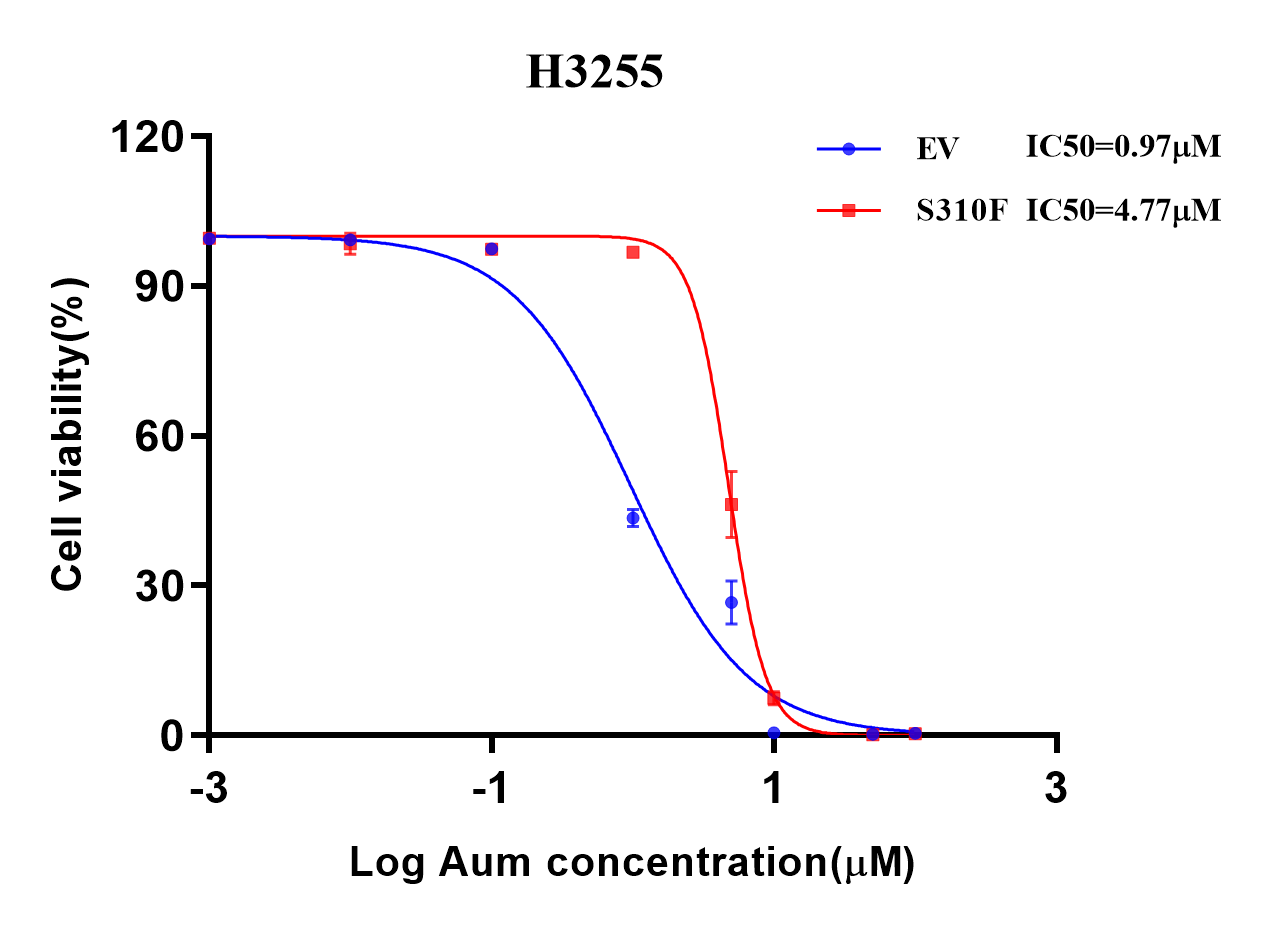

Supplement: Supplementary file 4 — Source data Fig. 1 [file 44321_2025_293_MOESM4_ESM.zip › Figure 1/1E/H3255 IC50.tif]

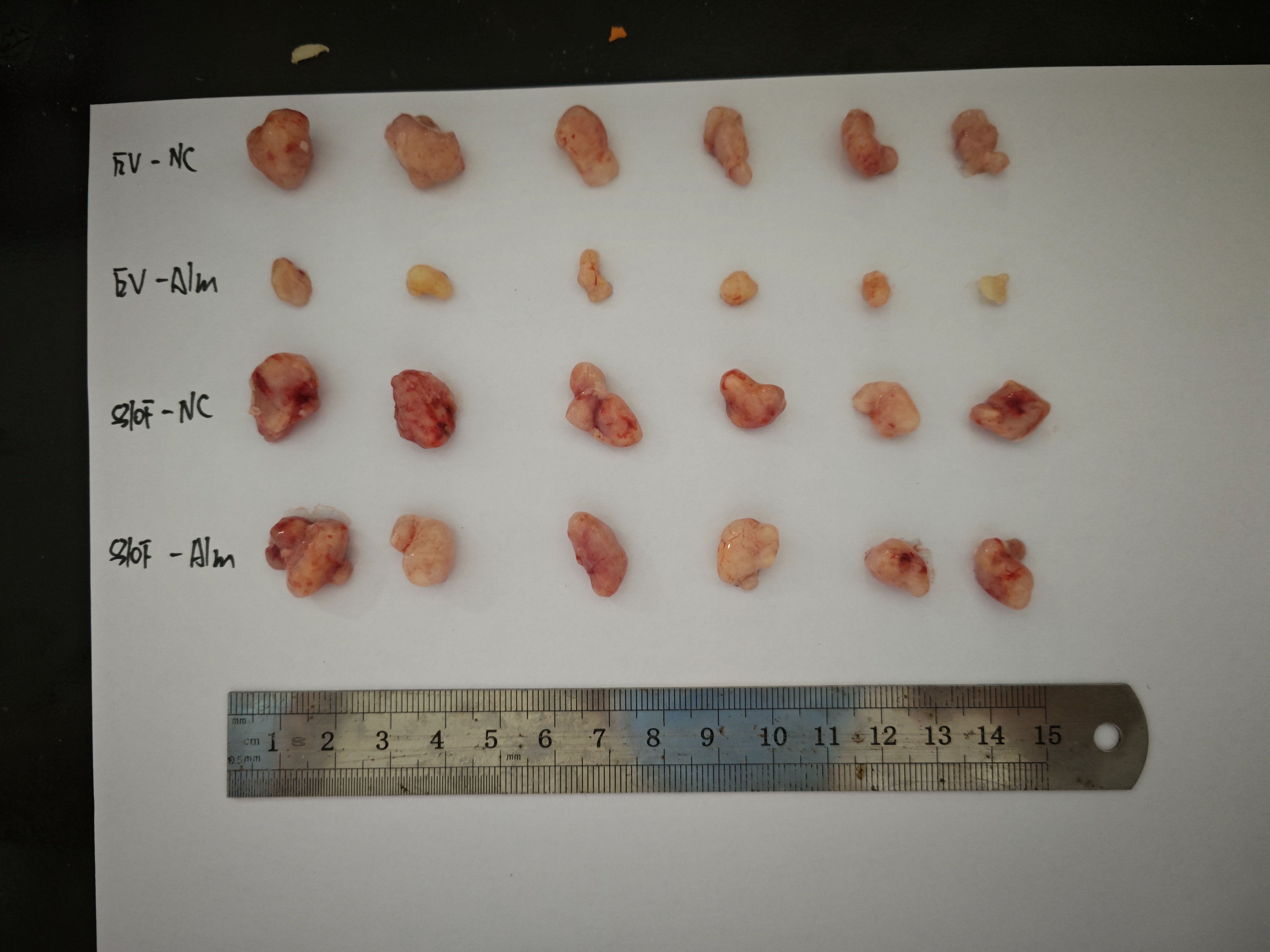

Supplement: Supplementary file 4 — Source data Fig. 1 [file 44321_2025_293_MOESM4_ESM.zip › Figure 1/1F/mice tumor.jpg]

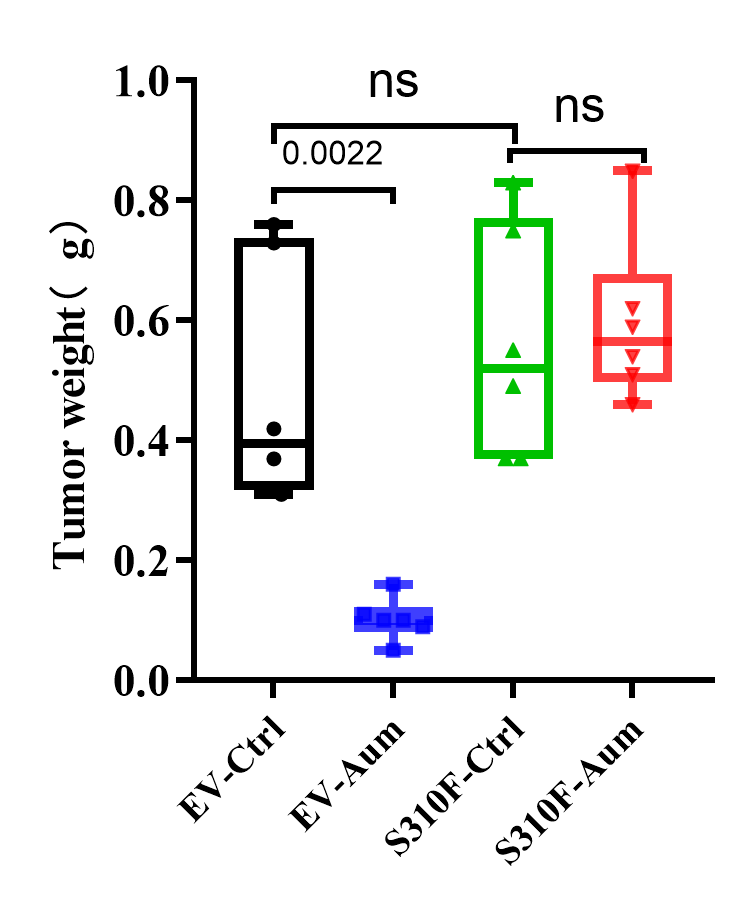

Supplement: Supplementary file 4 — Source data Fig. 1 [file 44321_2025_293_MOESM4_ESM.zip › Figure 1/1G/Tumor weight.tif]

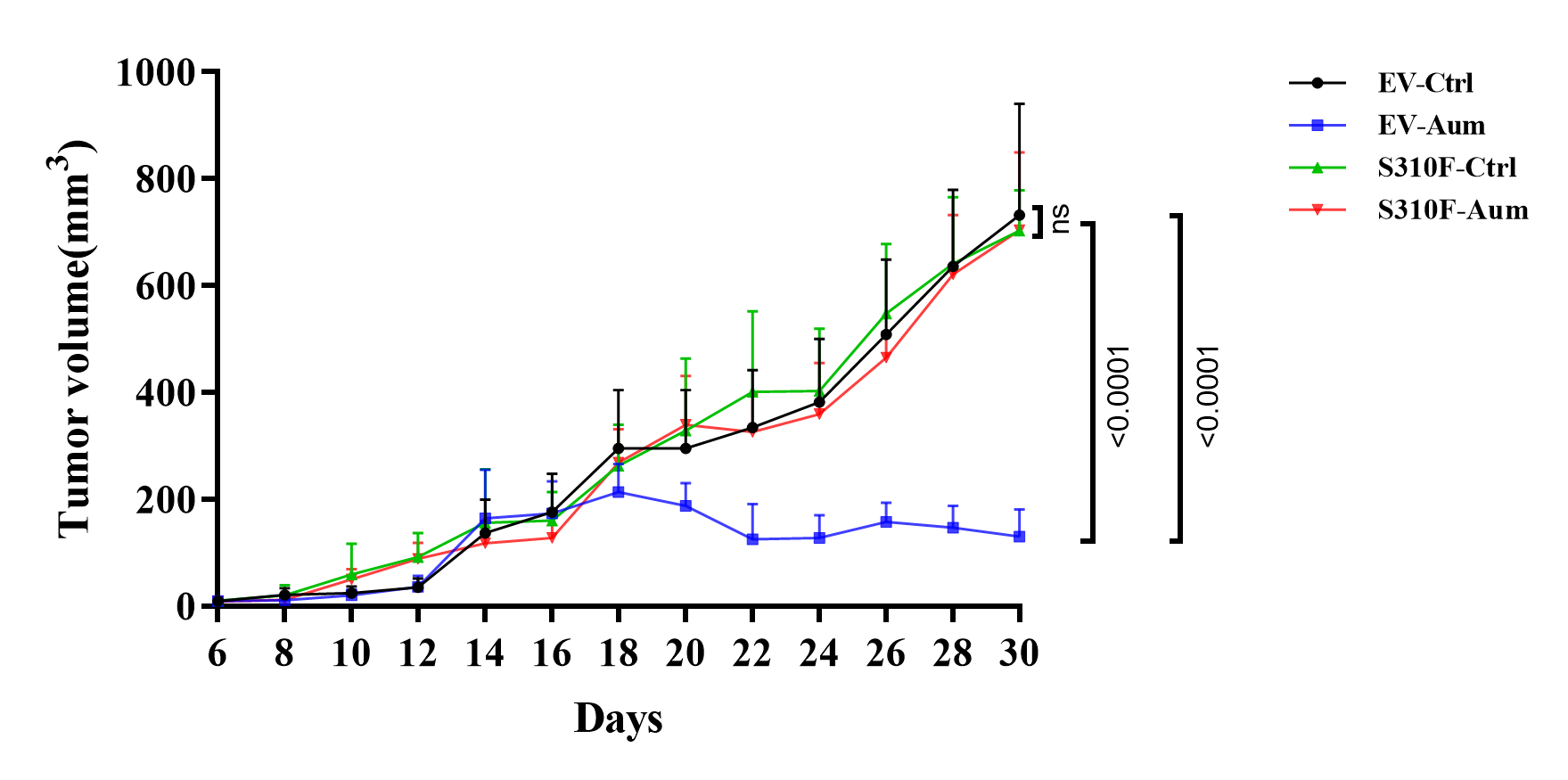

Supplement: Supplementary file 4 — Source data Fig. 1 [file 44321_2025_293_MOESM4_ESM.zip › Figure 1/1H/Tumor volume.tif]

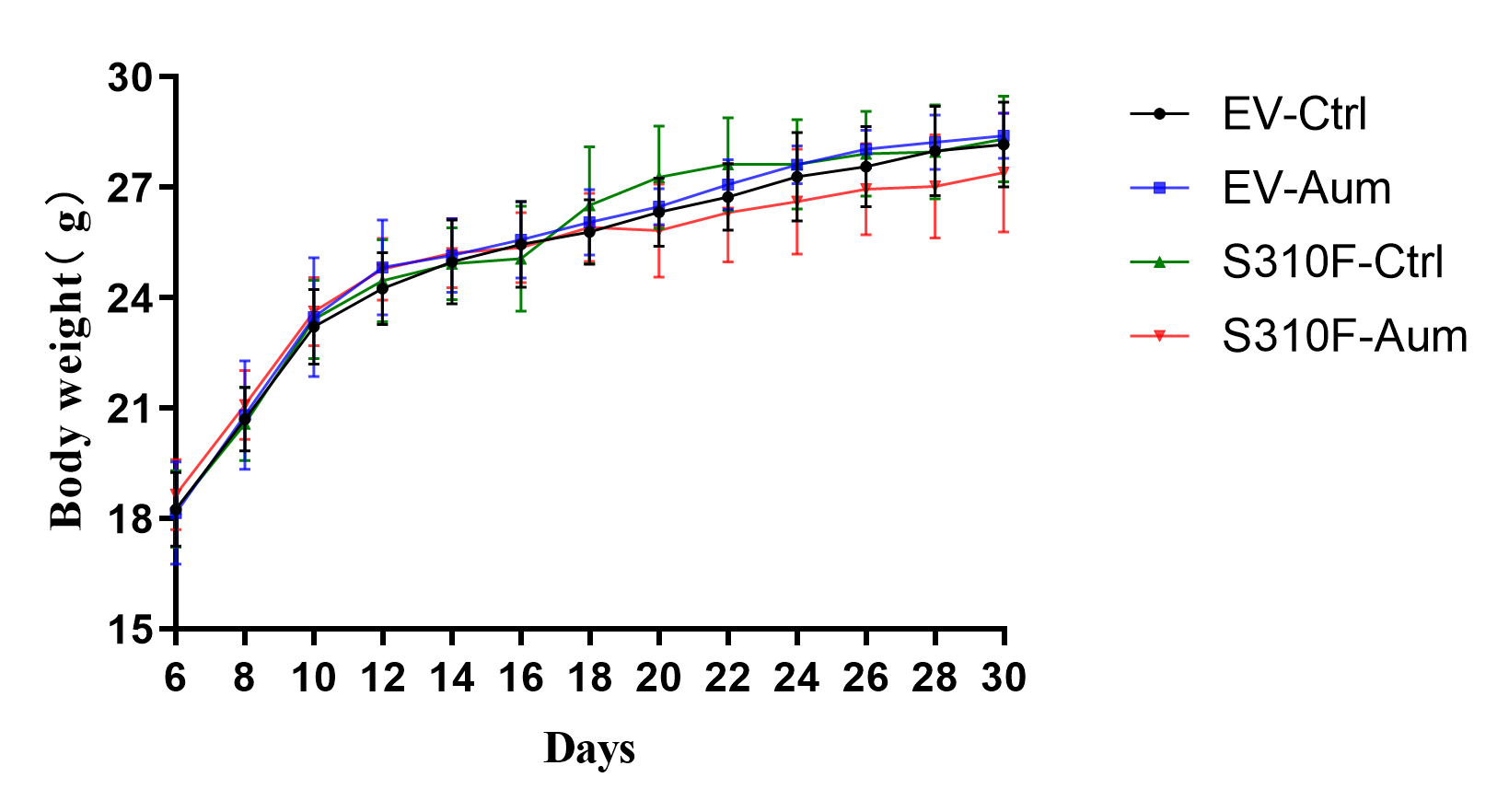

Supplement: Supplementary file 4 — Source data Fig. 1 [file 44321_2025_293_MOESM4_ESM.zip › Figure 1/1I/Body weight.tif]

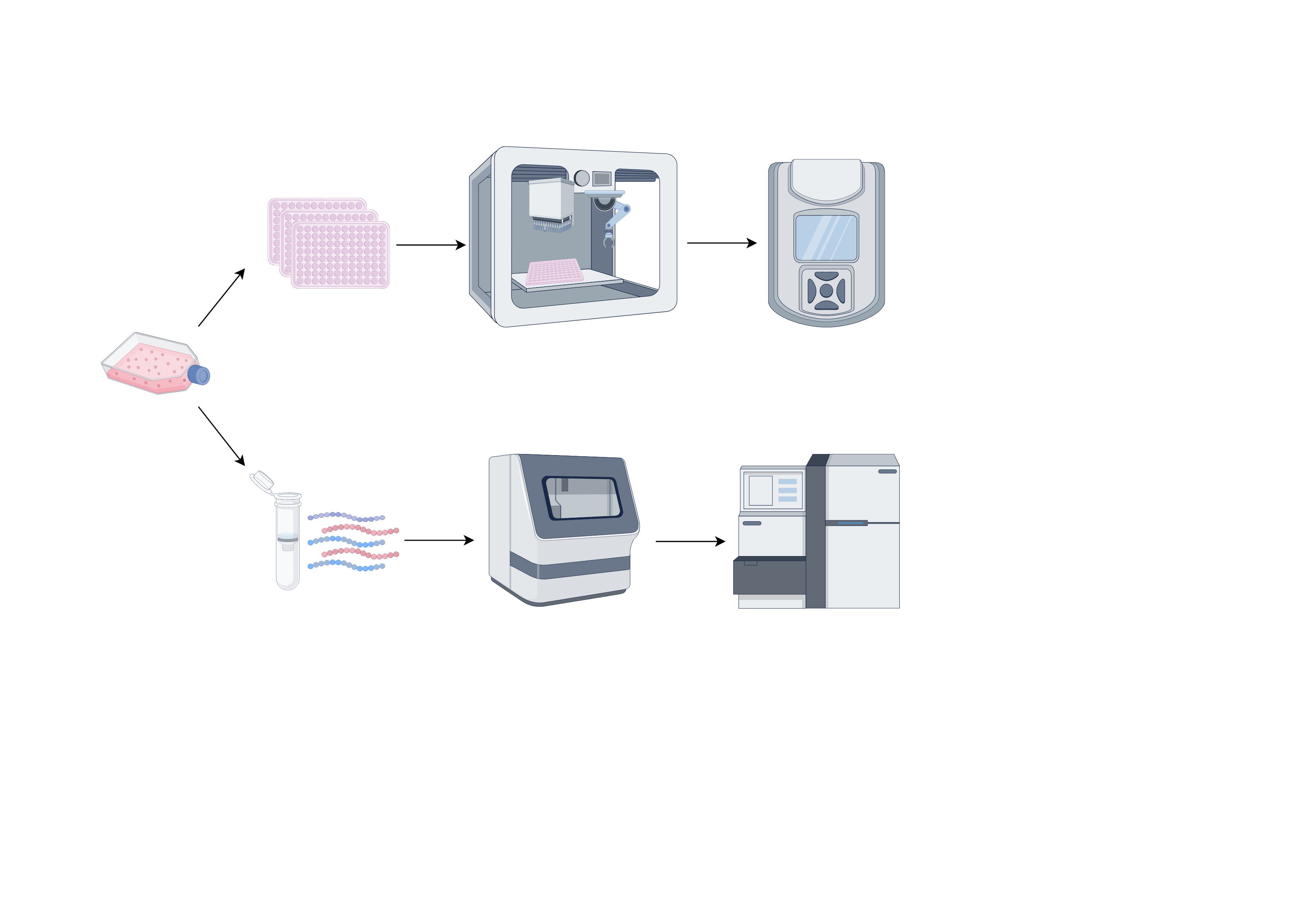

Supplement: Supplementary file 5 — Source data Fig. 2 [file 44321_2025_293_MOESM5_ESM.zip › Figure 2/2A/flowchart.tiff]

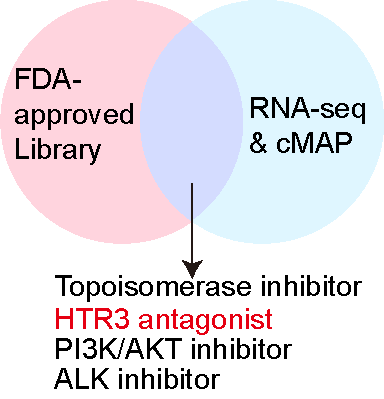

Supplement: Supplementary file 5 — Source data Fig. 2 [file 44321_2025_293_MOESM5_ESM.zip › Figure 2/2B/Fig 2B.tif]

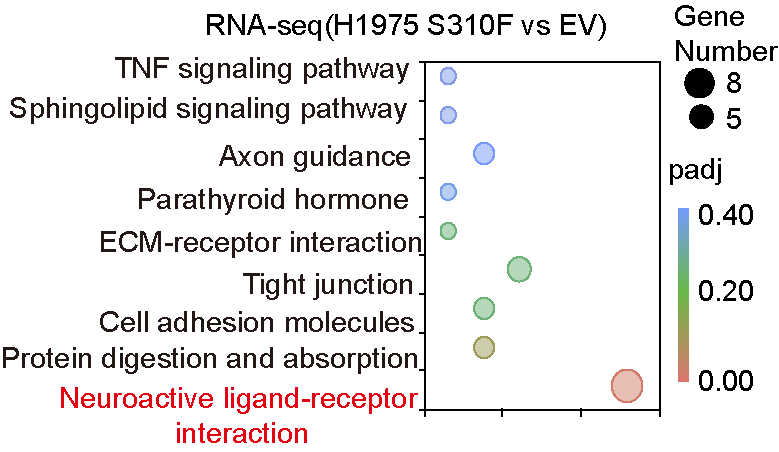

Supplement: Supplementary file 5 — Source data Fig. 2 [file 44321_2025_293_MOESM5_ESM.zip › Figure 2/2C/Fig 2C.tif]

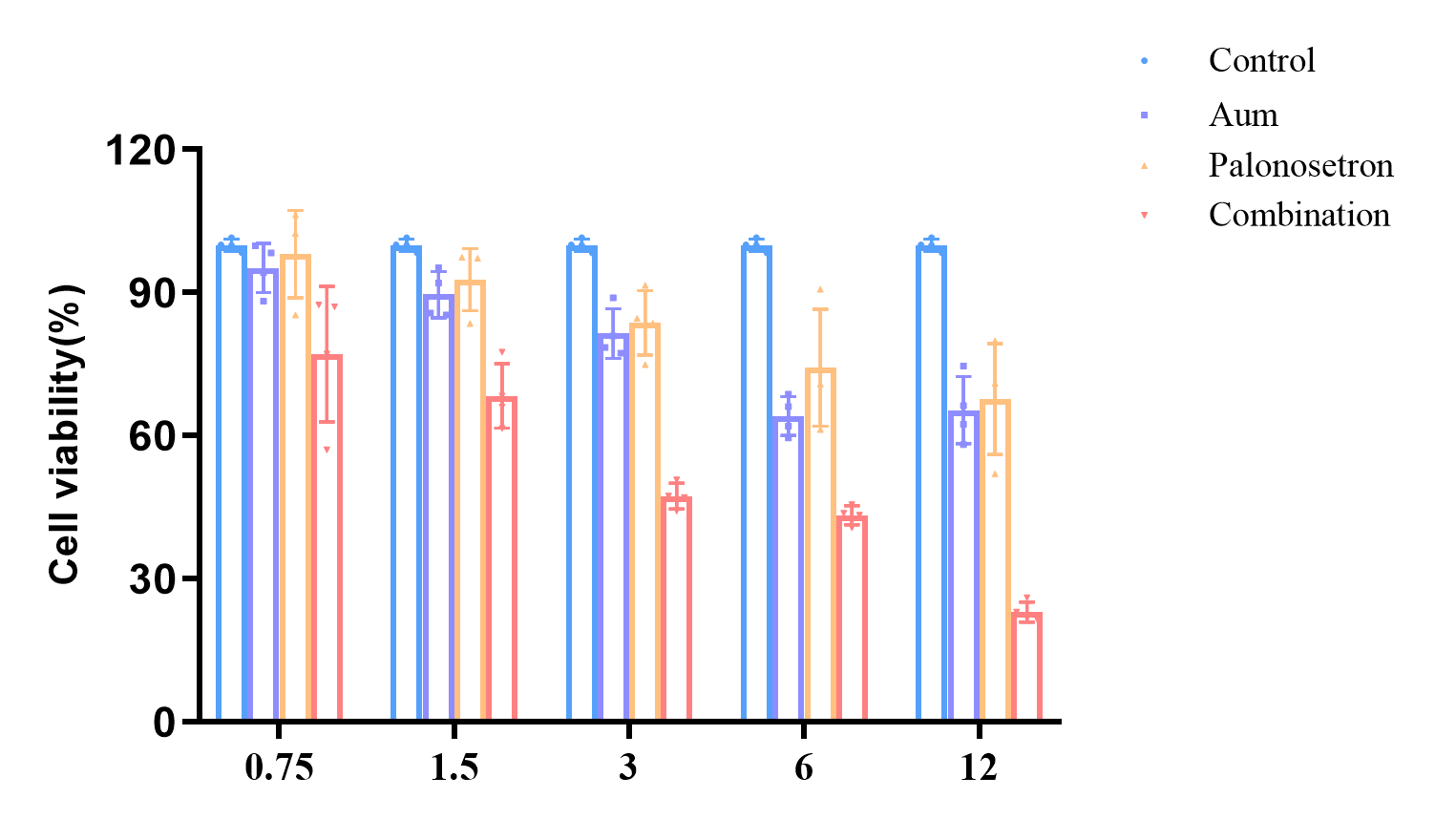

Supplement: Supplementary file 5 — Source data Fig. 2 [file 44321_2025_293_MOESM5_ESM.zip › Figure 2/2D/H1975 Palon+Aum CI.tif]

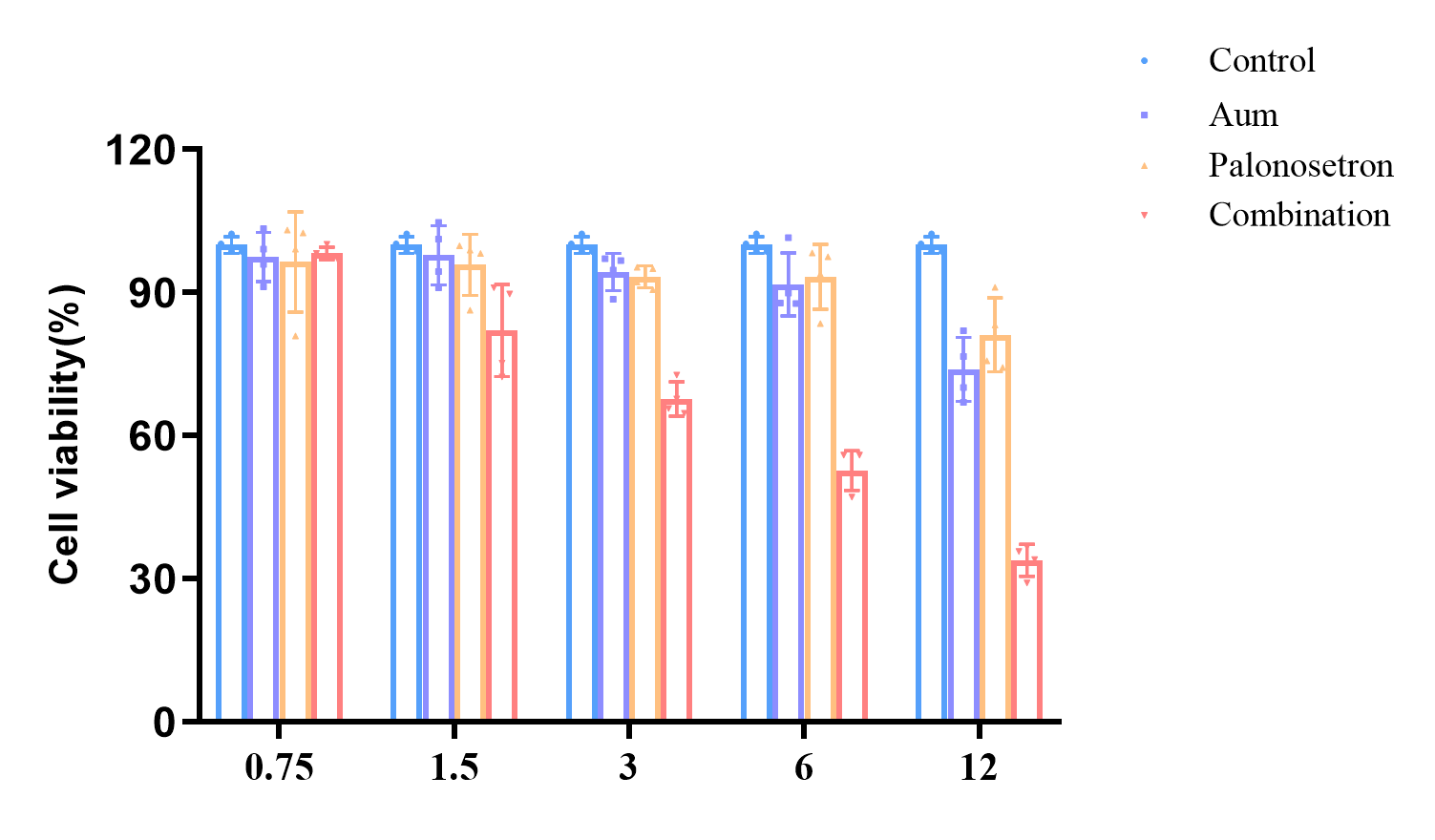

Supplement: Supplementary file 5 — Source data Fig. 2 [file 44321_2025_293_MOESM5_ESM.zip › Figure 2/2E/H3255 Palon+Aum.tif]

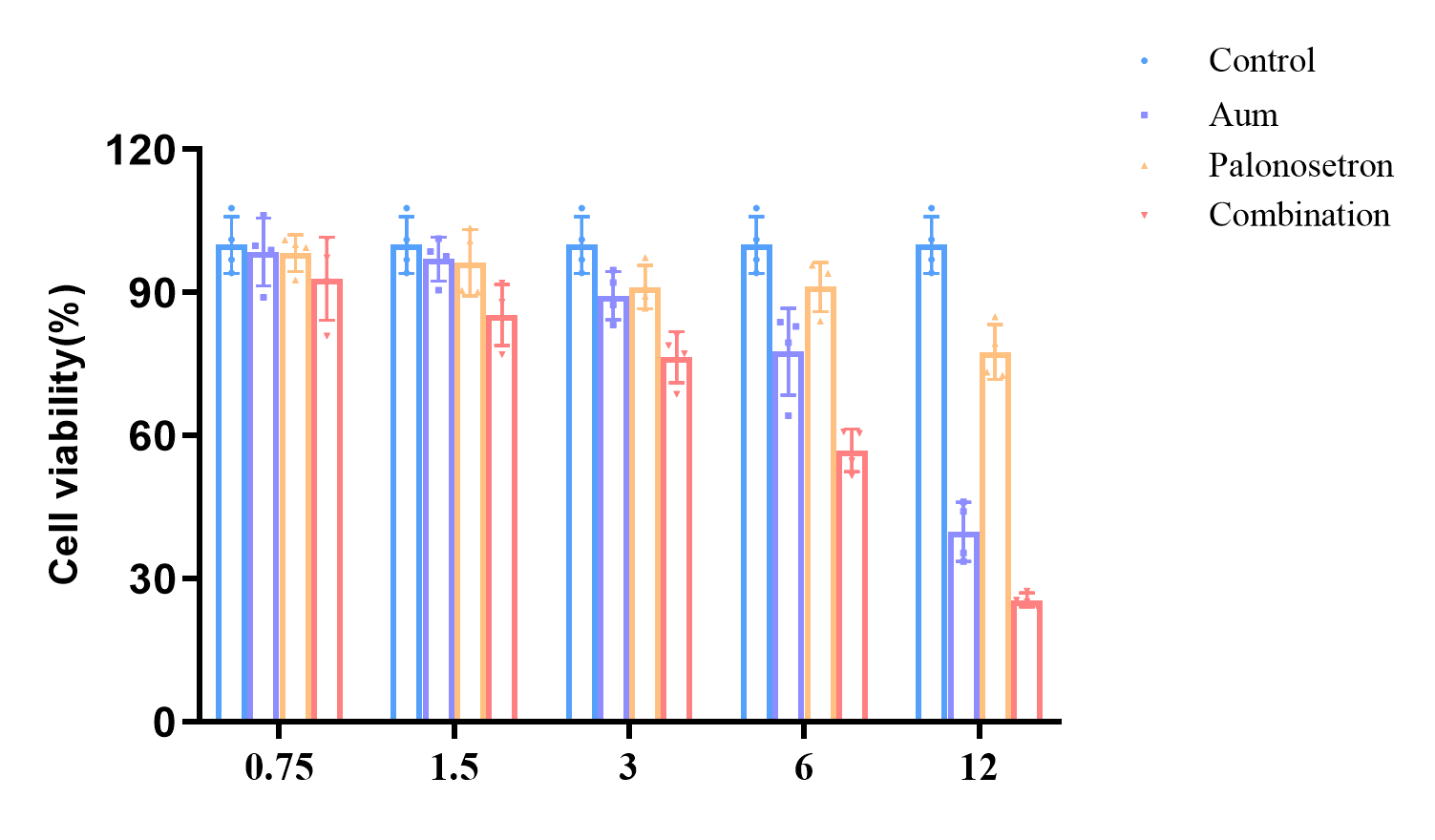

Supplement: Supplementary file 5 — Source data Fig. 2 [file 44321_2025_293_MOESM5_ESM.zip › Figure 2/2F/PC9 Palon+Aum.tif]

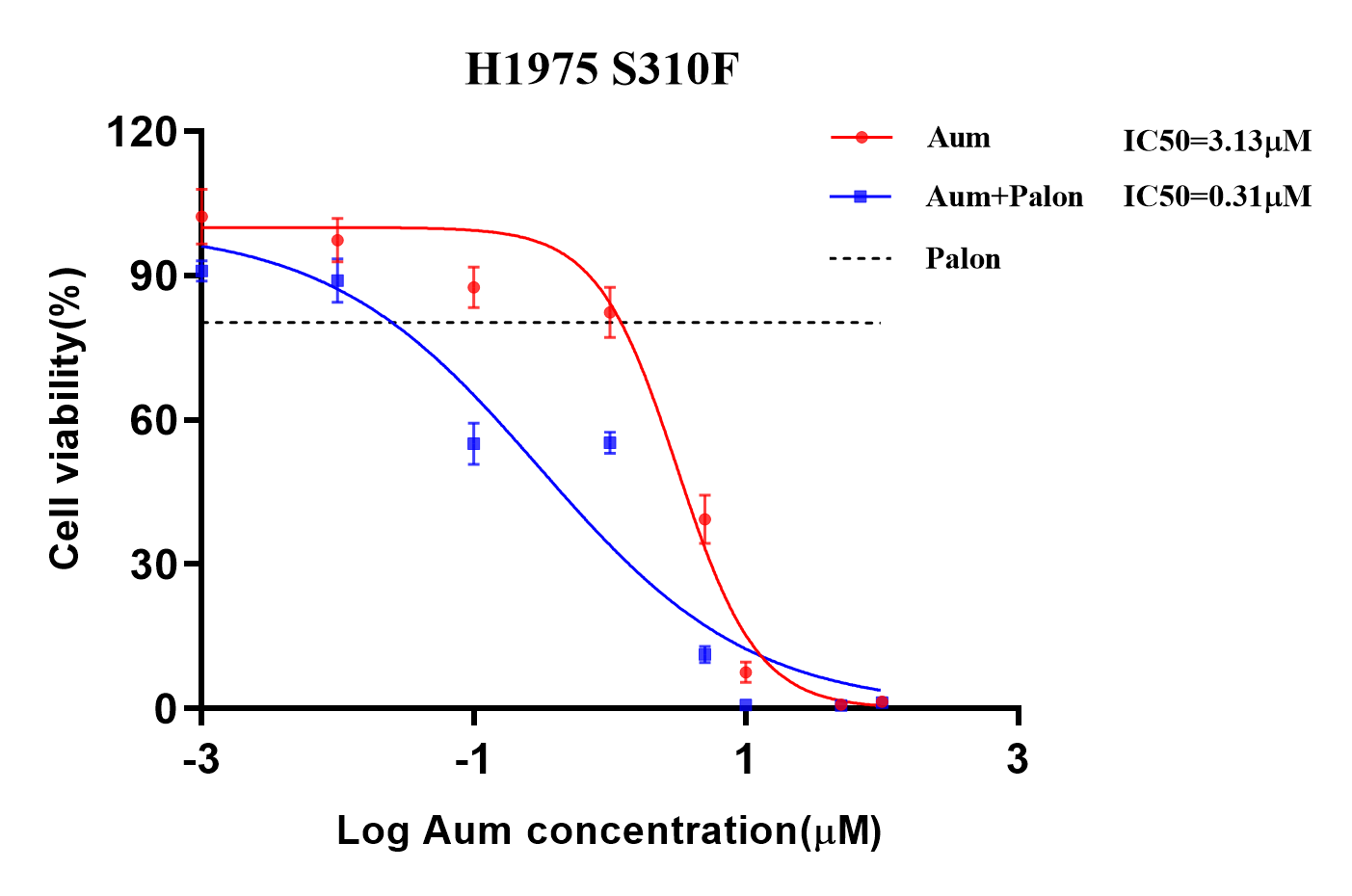

Supplement: Supplementary file 5 — Source data Fig. 2 [file 44321_2025_293_MOESM5_ESM.zip › Figure 2/2G/CCK8 Fig 2G.tif]

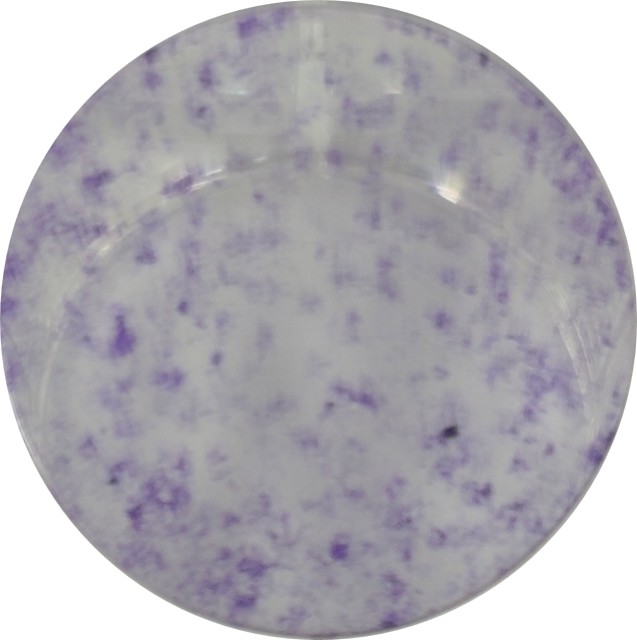

Supplement: Supplementary file 5 — Source data Fig. 2 [file 44321_2025_293_MOESM5_ESM.zip › Figure 2/2H/Aum/Aum-1.jpg]

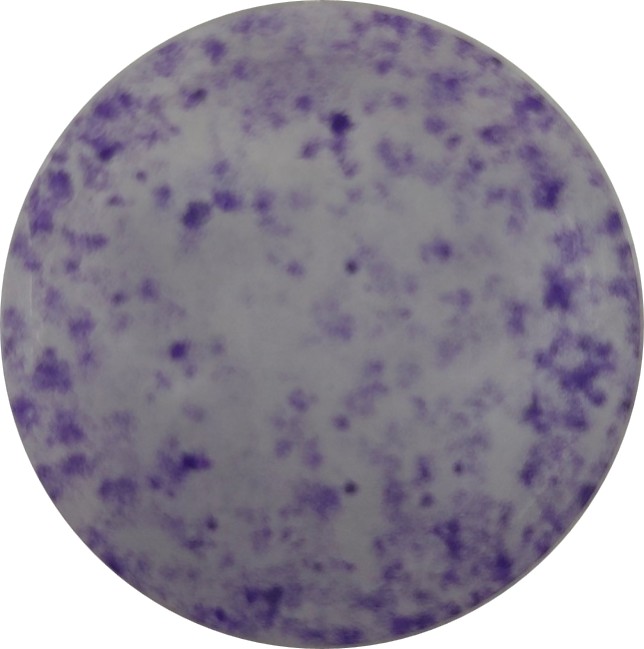

Supplement: Supplementary file 5 — Source data Fig. 2 [file 44321_2025_293_MOESM5_ESM.zip › Figure 2/2H/Aum/Aum-2.jpg]

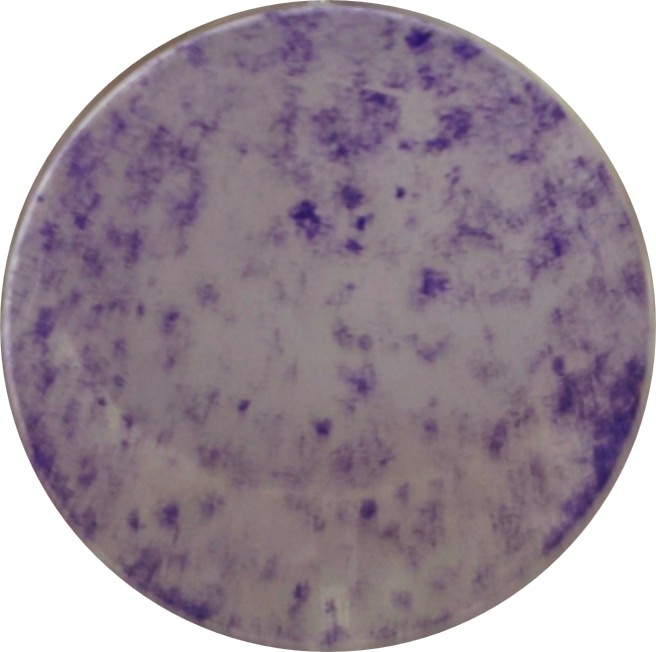

Supplement: Supplementary file 5 — Source data Fig. 2 [file 44321_2025_293_MOESM5_ESM.zip › Figure 2/2H/Aum/Aum-3.jpg]

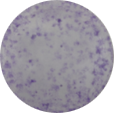

Supplement: Supplementary file 5 — Source data Fig. 2 [file 44321_2025_293_MOESM5_ESM.zip › Figure 2/2H/Aum/Aum1.1.png]

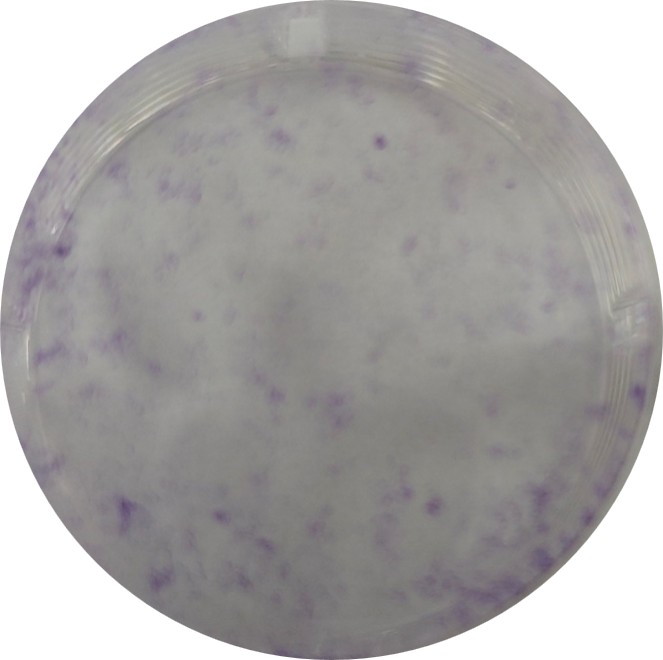

Supplement: Supplementary file 5 — Source data Fig. 2 [file 44321_2025_293_MOESM5_ESM.zip › Figure 2/2H/Comb/Comb-1.jpg]

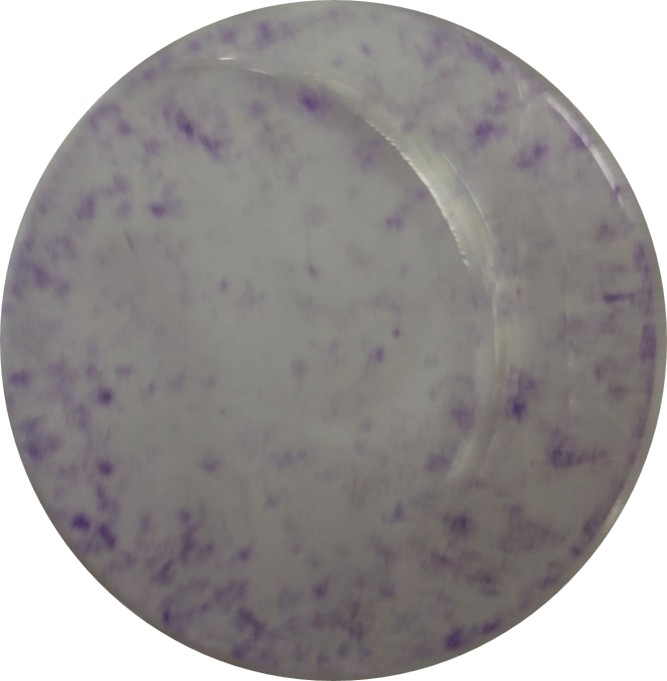

Supplement: Supplementary file 5 — Source data Fig. 2 [file 44321_2025_293_MOESM5_ESM.zip › Figure 2/2H/Comb/Comb-2.jpg]

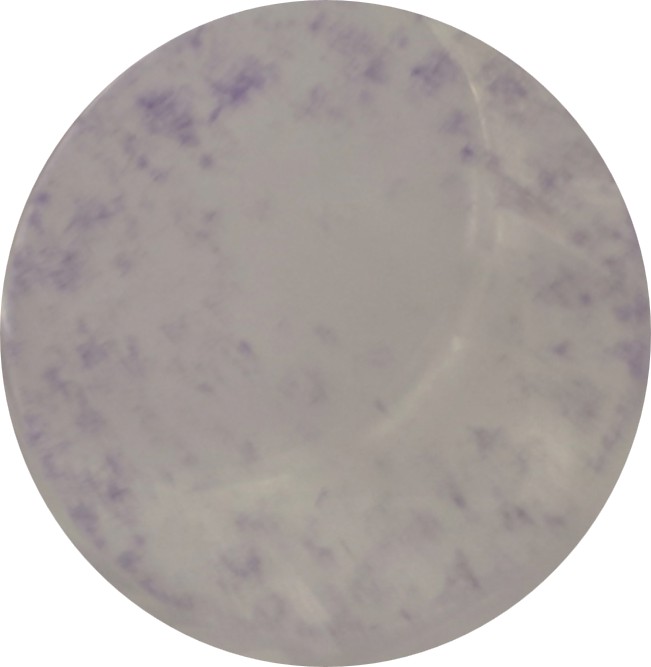

Supplement: Supplementary file 5 — Source data Fig. 2 [file 44321_2025_293_MOESM5_ESM.zip › Figure 2/2H/Comb/Comb-3.jpg]

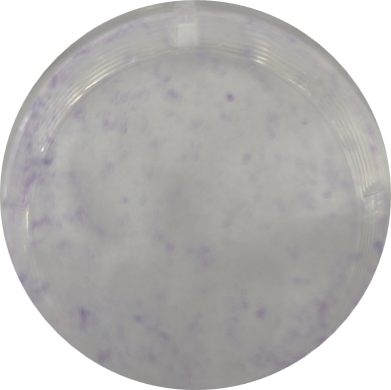

Supplement: Supplementary file 5 — Source data Fig. 2 [file 44321_2025_293_MOESM5_ESM.zip › Figure 2/2H/Comb/Comb1.1.png]

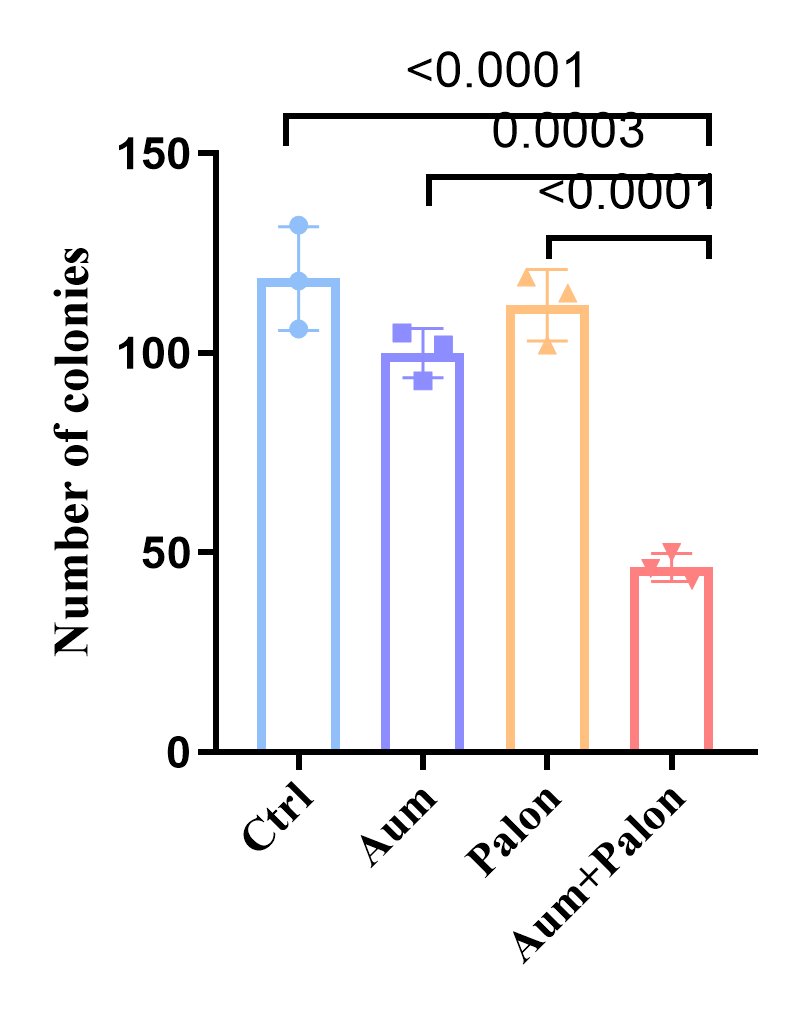

Supplement: Supplementary file 5 — Source data Fig. 2 [file 44321_2025_293_MOESM5_ESM.zip › Figure 2/2H/Fig 2H.tif]

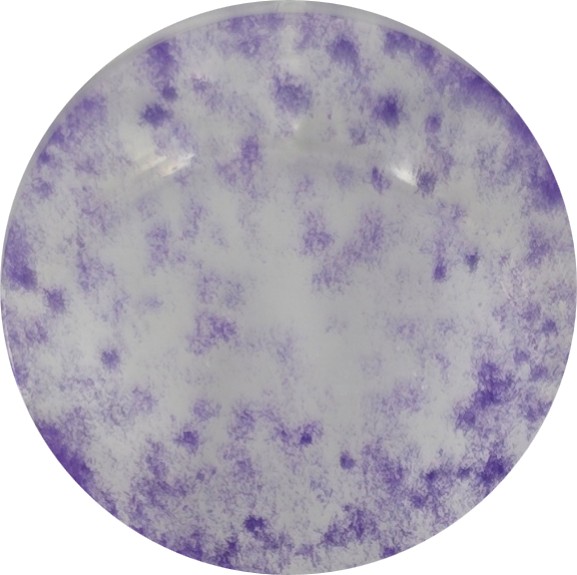

Supplement: Supplementary file 5 — Source data Fig. 2 [file 44321_2025_293_MOESM5_ESM.zip › Figure 2/2H/NC/NC-1.jpg]

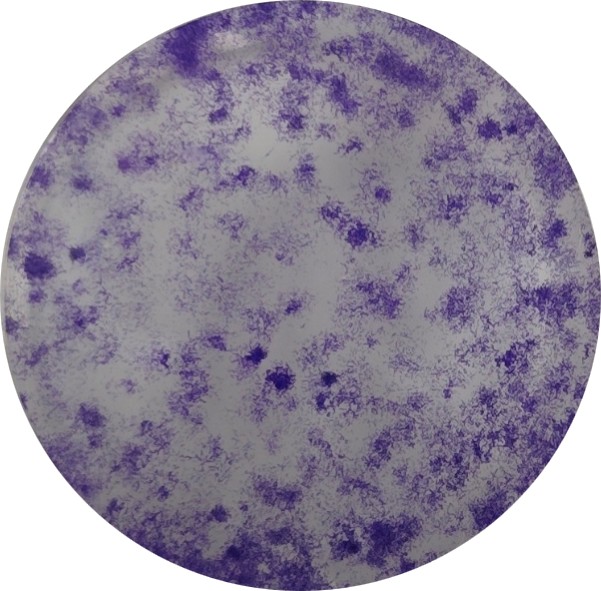

Supplement: Supplementary file 5 — Source data Fig. 2 [file 44321_2025_293_MOESM5_ESM.zip › Figure 2/2H/NC/NC-2.jpg]

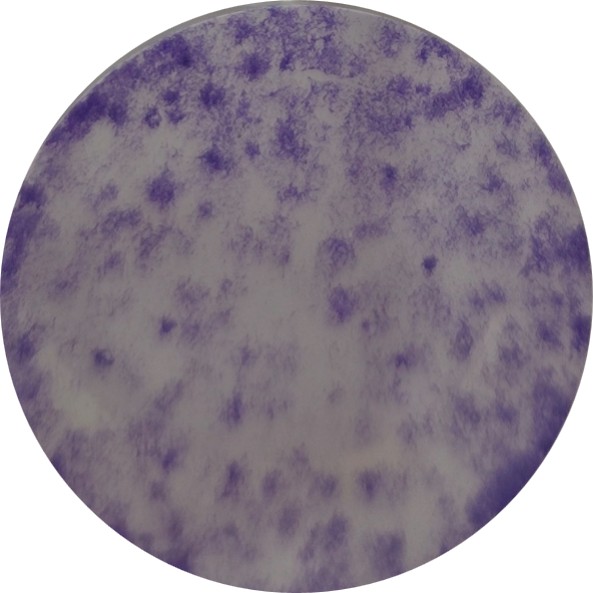

Supplement: Supplementary file 5 — Source data Fig. 2 [file 44321_2025_293_MOESM5_ESM.zip › Figure 2/2H/NC/NC-3.jpg]

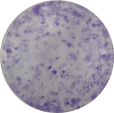

Supplement: Supplementary file 5 — Source data Fig. 2 [file 44321_2025_293_MOESM5_ESM.zip › Figure 2/2H/NC/NC.1.png]

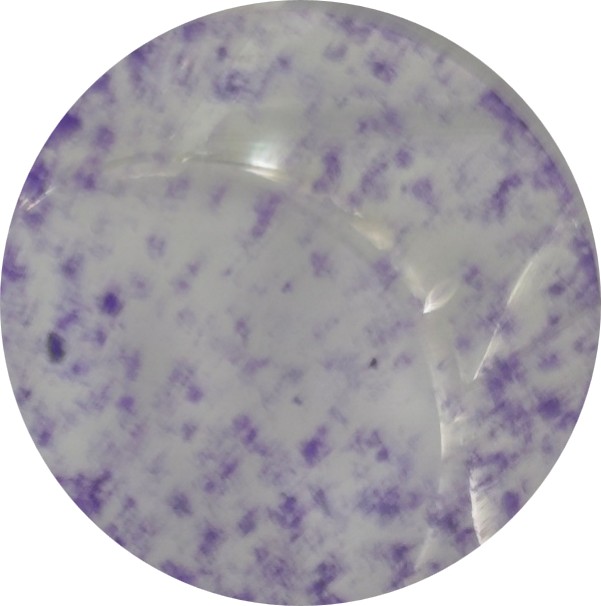

Supplement: Supplementary file 5 — Source data Fig. 2 [file 44321_2025_293_MOESM5_ESM.zip › Figure 2/2H/Palon/Palon-1.jpg]

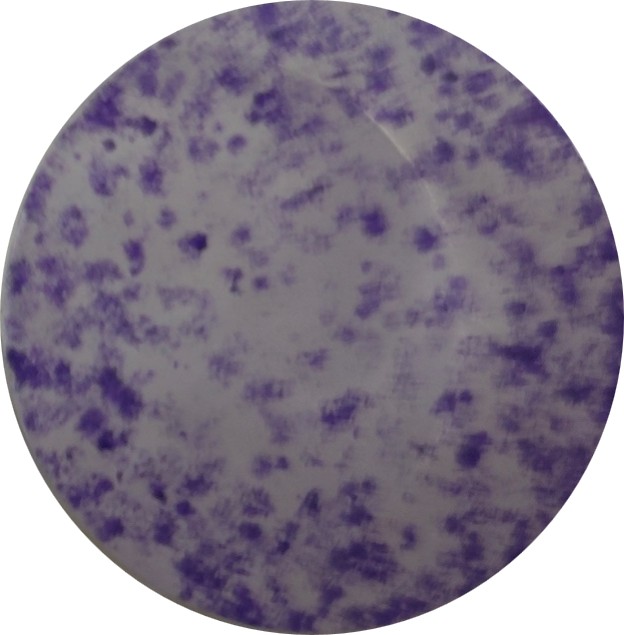

Supplement: Supplementary file 5 — Source data Fig. 2 [file 44321_2025_293_MOESM5_ESM.zip › Figure 2/2H/Palon/Palon-2.jpg]

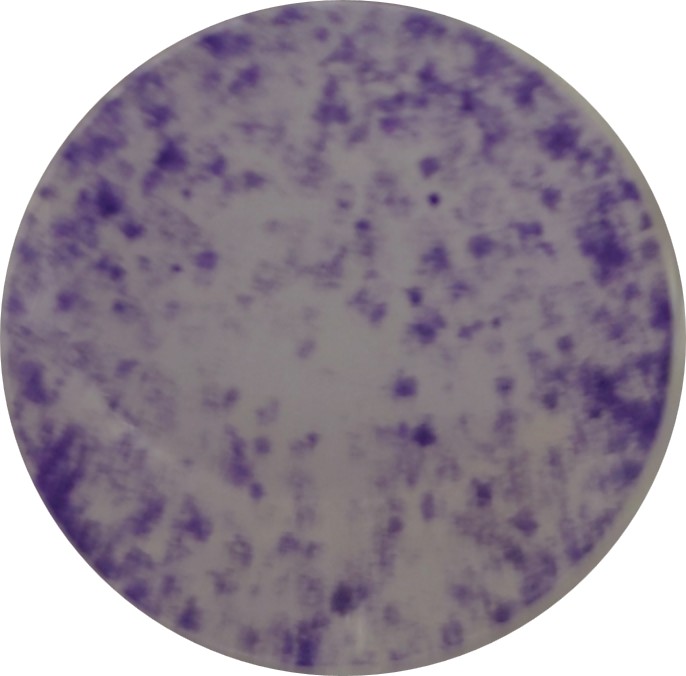

Supplement: Supplementary file 5 — Source data Fig. 2 [file 44321_2025_293_MOESM5_ESM.zip › Figure 2/2H/Palon/Palon-3.jpg]

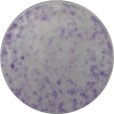

Supplement: Supplementary file 5 — Source data Fig. 2 [file 44321_2025_293_MOESM5_ESM.zip › Figure 2/2H/Palon/Palon1.1.png]

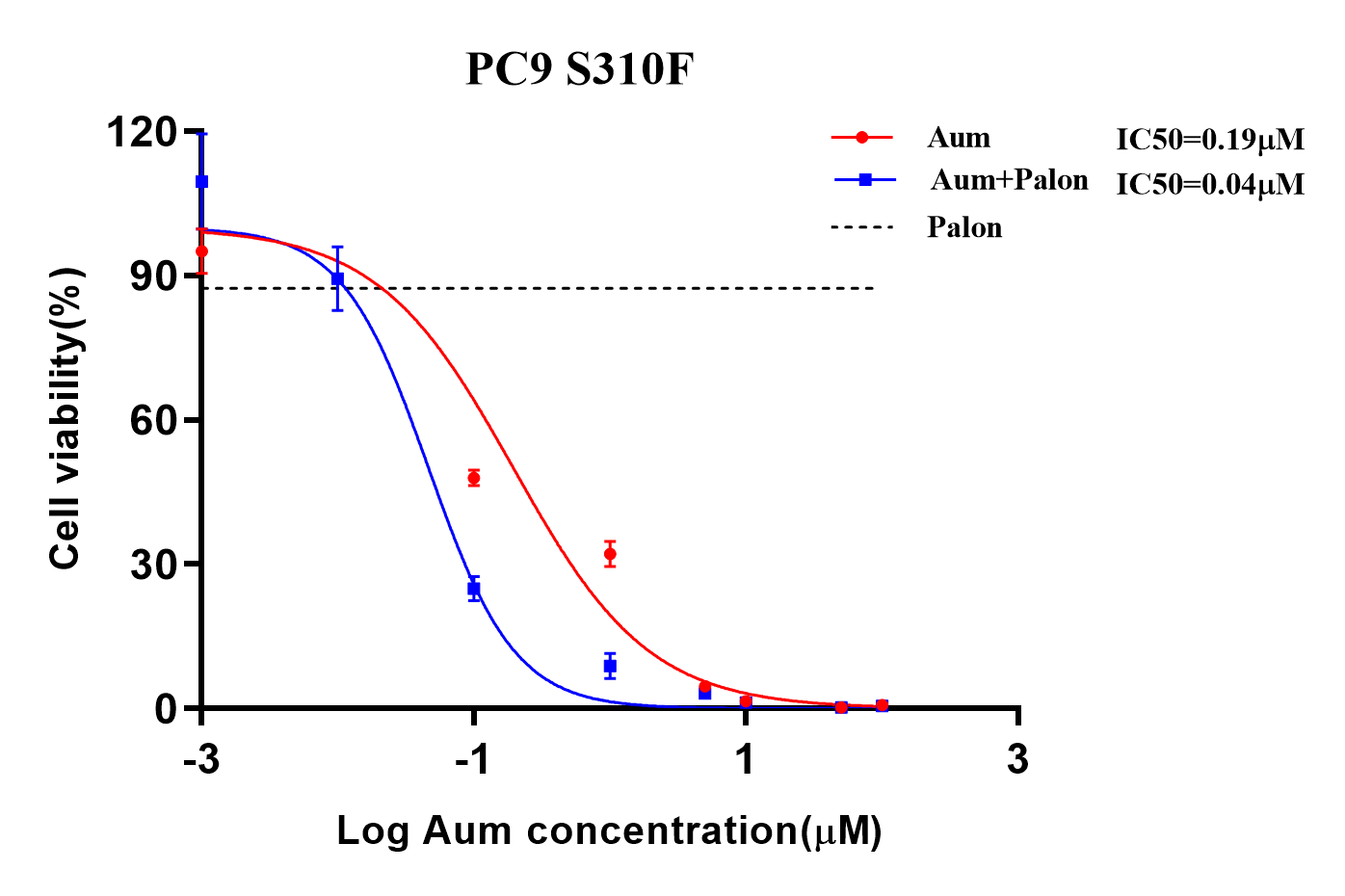

Supplement: Supplementary file 5 — Source data Fig. 2 [file 44321_2025_293_MOESM5_ESM.zip › Figure 2/2I/Fig 2I.tif]

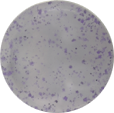

Supplement: Supplementary file 5 — Source data Fig. 2 [file 44321_2025_293_MOESM5_ESM.zip › Figure 2/2J/Aum/Aum1.1.png]

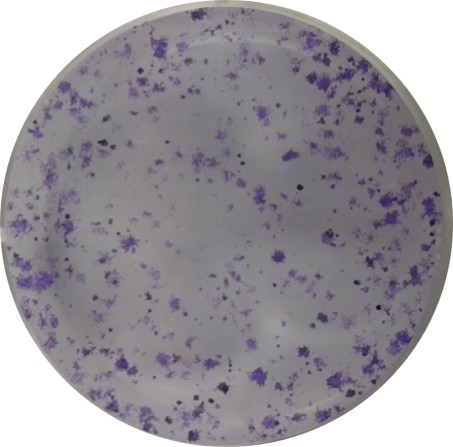

Supplement: Supplementary file 5 — Source data Fig. 2 [file 44321_2025_293_MOESM5_ESM.zip › Figure 2/2J/Aum/Aum1.jpg]

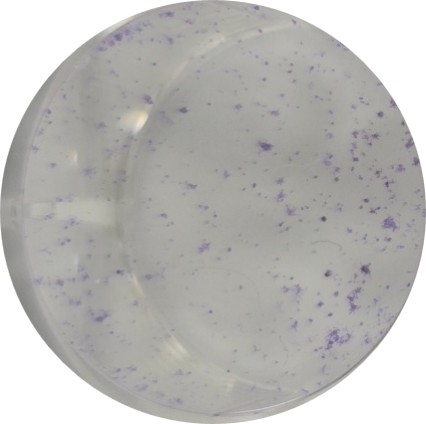

Supplement: Supplementary file 5 — Source data Fig. 2 [file 44321_2025_293_MOESM5_ESM.zip › Figure 2/2J/Aum/Aum2.jpg]

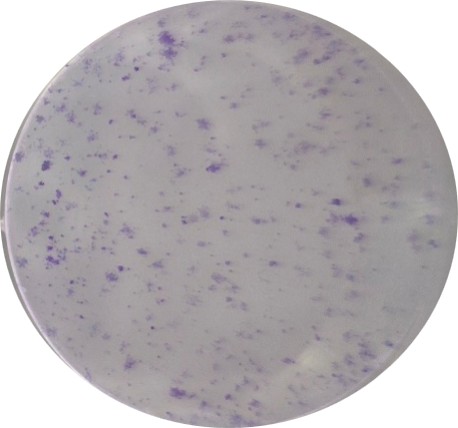

Supplement: Supplementary file 5 — Source data Fig. 2 [file 44321_2025_293_MOESM5_ESM.zip › Figure 2/2J/Aum/Aum3.jpg]

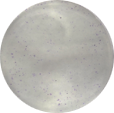

Supplement: Supplementary file 5 — Source data Fig. 2 [file 44321_2025_293_MOESM5_ESM.zip › Figure 2/2J/Comb/Comb1.1.png]

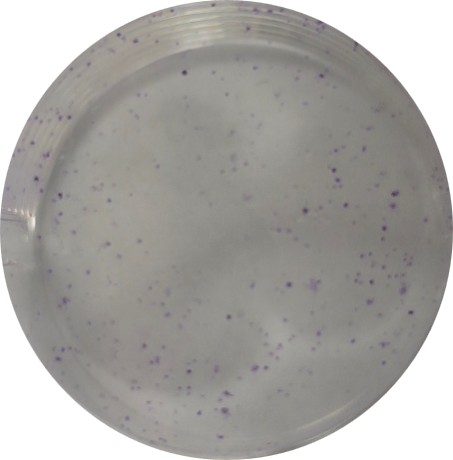

Supplement: Supplementary file 5 — Source data Fig. 2 [file 44321_2025_293_MOESM5_ESM.zip › Figure 2/2J/Comb/Comb1.jpg]

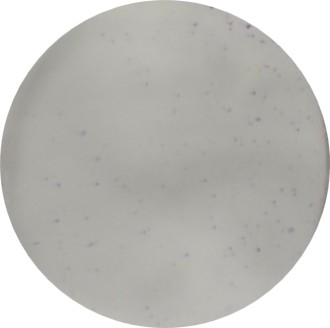

Supplement: Supplementary file 5 — Source data Fig. 2 [file 44321_2025_293_MOESM5_ESM.zip › Figure 2/2J/Comb/Comb2.jpg]

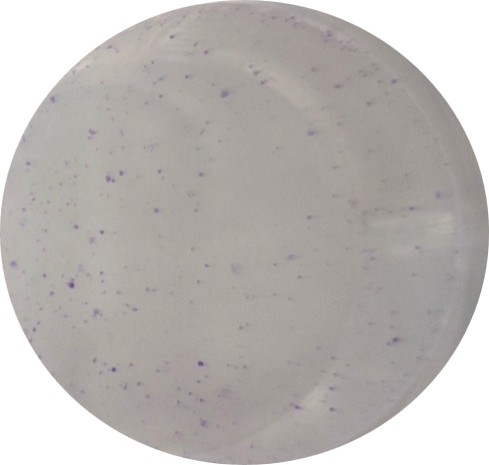

Supplement: Supplementary file 5 — Source data Fig. 2 [file 44321_2025_293_MOESM5_ESM.zip › Figure 2/2J/Comb/Comb3.jpg]

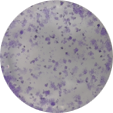

Supplement: Supplementary file 5 — Source data Fig. 2 [file 44321_2025_293_MOESM5_ESM.zip › Figure 2/2J/NC/NC1.1.png]

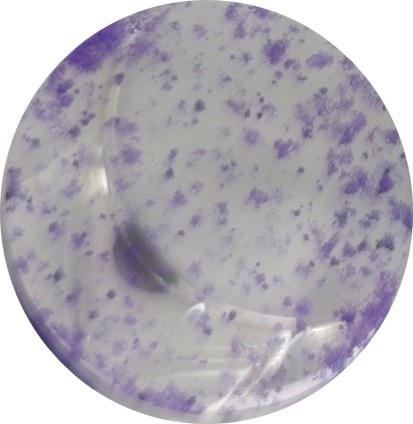

Supplement: Supplementary file 5 — Source data Fig. 2 [file 44321_2025_293_MOESM5_ESM.zip › Figure 2/2J/NC/NC1.jpg]

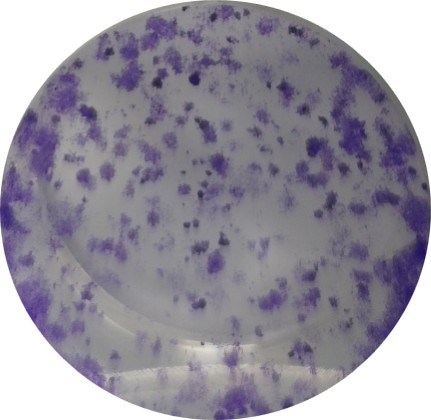

Supplement: Supplementary file 5 — Source data Fig. 2 [file 44321_2025_293_MOESM5_ESM.zip › Figure 2/2J/NC/NC2.jpg]

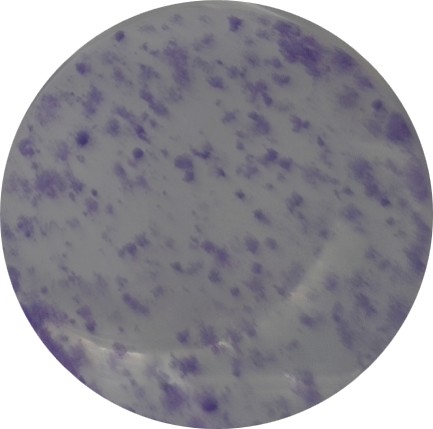

Supplement: Supplementary file 5 — Source data Fig. 2 [file 44321_2025_293_MOESM5_ESM.zip › Figure 2/2J/NC/NC3.jpg]

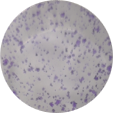

Supplement: Supplementary file 5 — Source data Fig. 2 [file 44321_2025_293_MOESM5_ESM.zip › Figure 2/2J/Palon/Palon1.1.png]

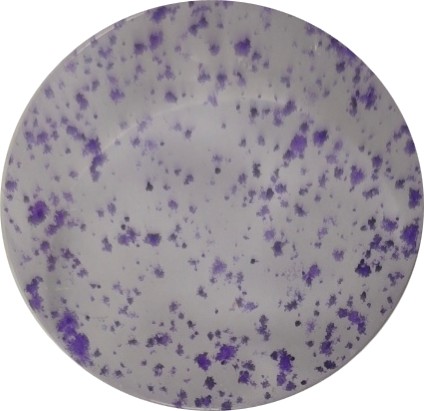

Supplement: Supplementary file 5 — Source data Fig. 2 [file 44321_2025_293_MOESM5_ESM.zip › Figure 2/2J/Palon/Palon1.jpg]

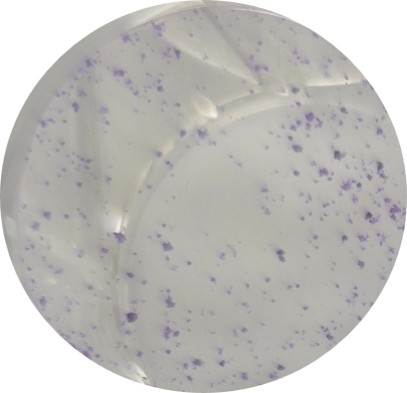

Supplement: Supplementary file 5 — Source data Fig. 2 [file 44321_2025_293_MOESM5_ESM.zip › Figure 2/2J/Palon/Palon2.jpg]

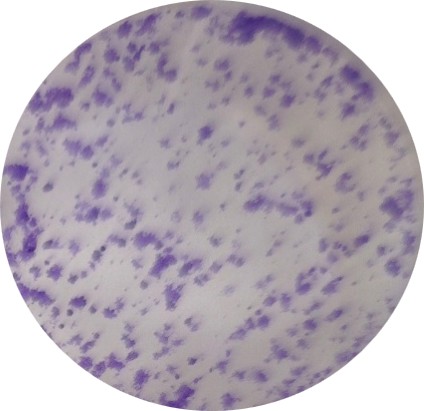

Supplement: Supplementary file 5 — Source data Fig. 2 [file 44321_2025_293_MOESM5_ESM.zip › Figure 2/2J/Palon/Palon3.jpg]

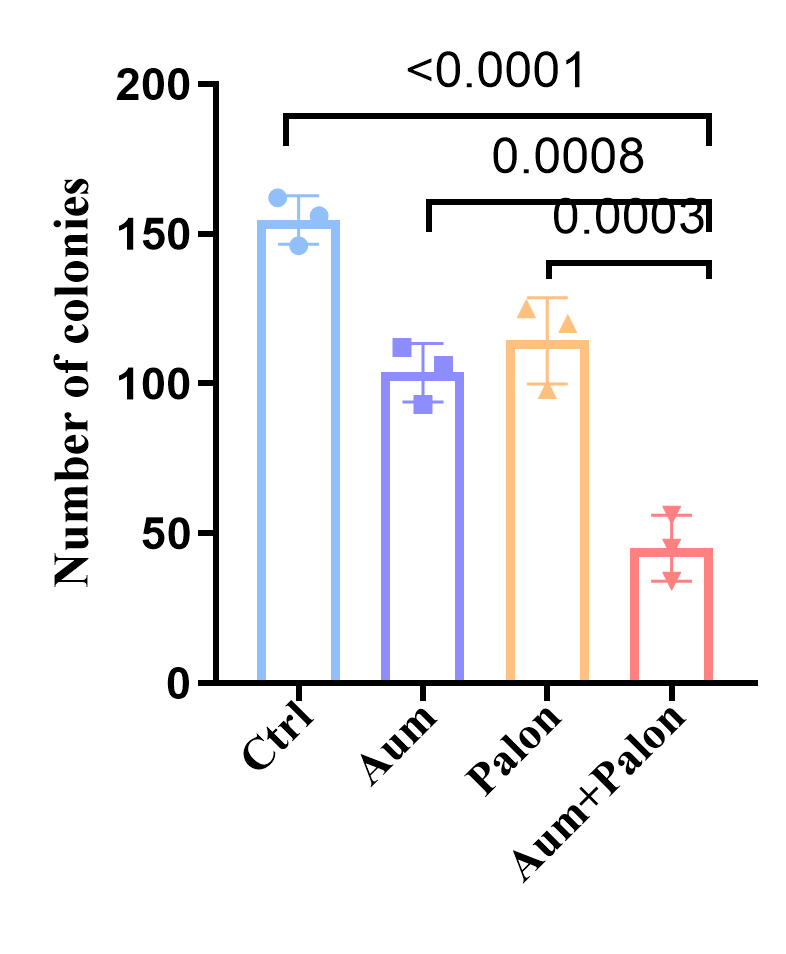

Supplement: Supplementary file 5 — Source data Fig. 2 [file 44321_2025_293_MOESM5_ESM.zip › Figure 2/2J/PC9 S310F.tif]

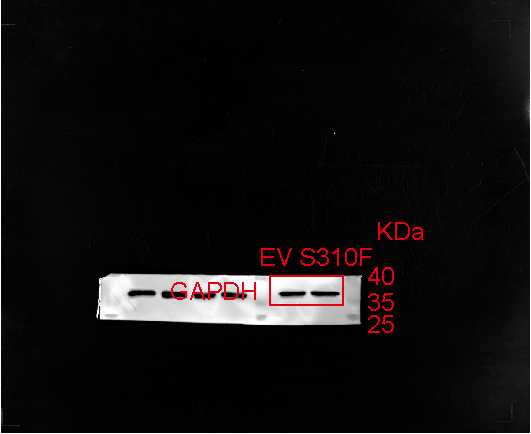

Supplement: Supplementary file 6 — Source data Fig. 3 [file 44321_2025_293_MOESM6_ESM.zip › Figure 3/3A/western GAPDH.tif]

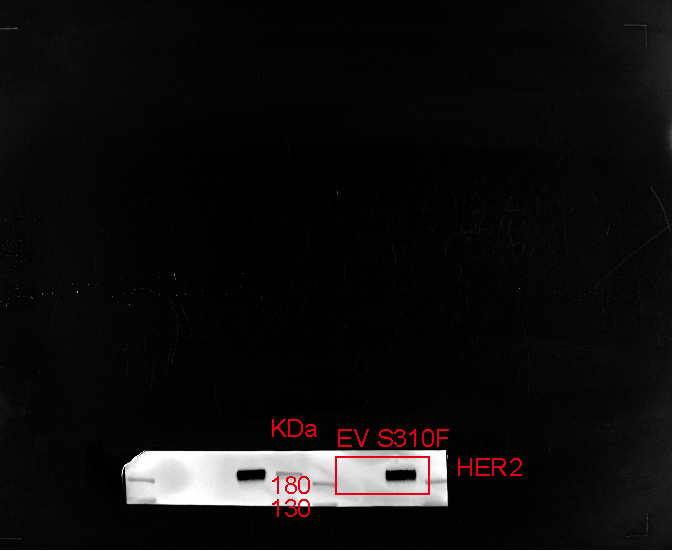

Supplement: Supplementary file 6 — Source data Fig. 3 [file 44321_2025_293_MOESM6_ESM.zip › Figure 3/3A/western HER2.tif]

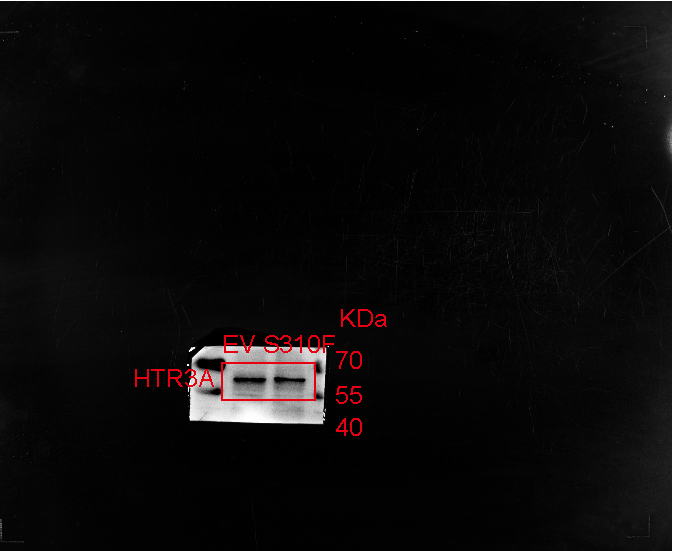

Supplement: Supplementary file 6 — Source data Fig. 3 [file 44321_2025_293_MOESM6_ESM.zip › Figure 3/3A/western HTR3A.tif]

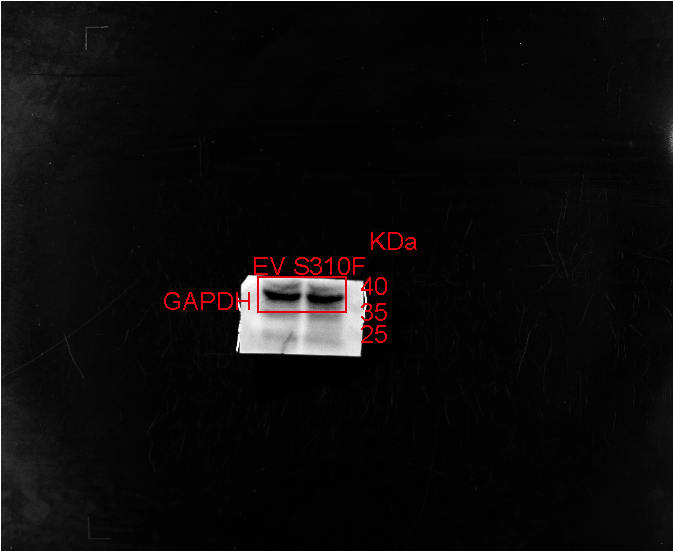

Supplement: Supplementary file 6 — Source data Fig. 3 [file 44321_2025_293_MOESM6_ESM.zip › Figure 3/3B/western GAPDH.tif]

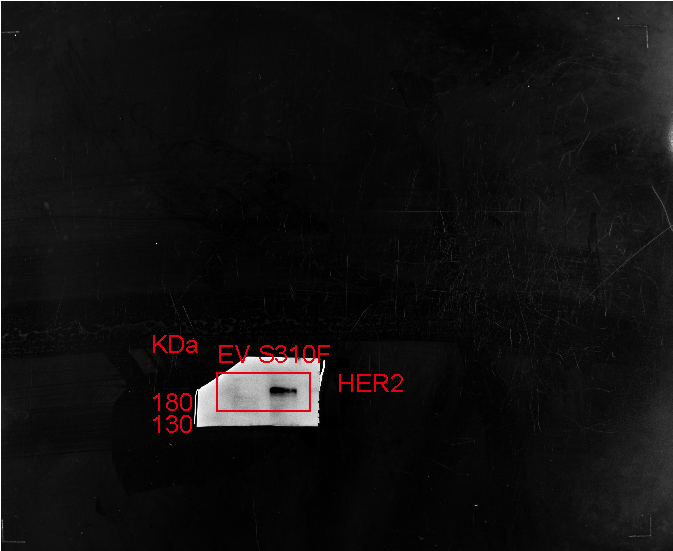

Supplement: Supplementary file 6 — Source data Fig. 3 [file 44321_2025_293_MOESM6_ESM.zip › Figure 3/3B/western HER2.tif]

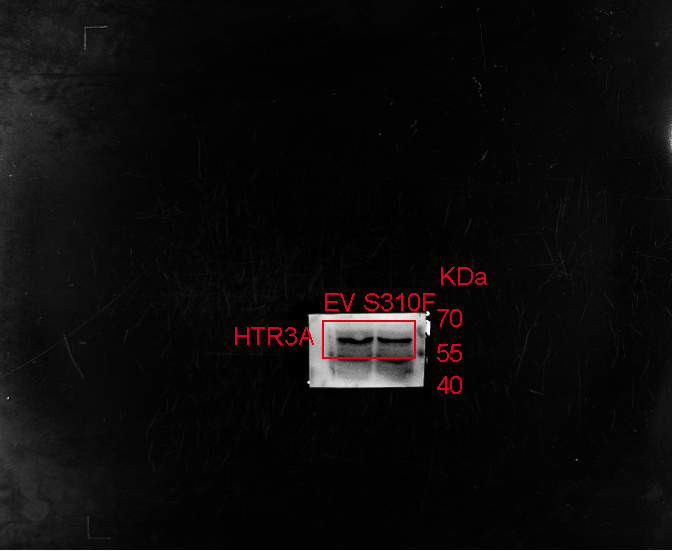

Supplement: Supplementary file 6 — Source data Fig. 3 [file 44321_2025_293_MOESM6_ESM.zip › Figure 3/3B/western HTR3A.tif]

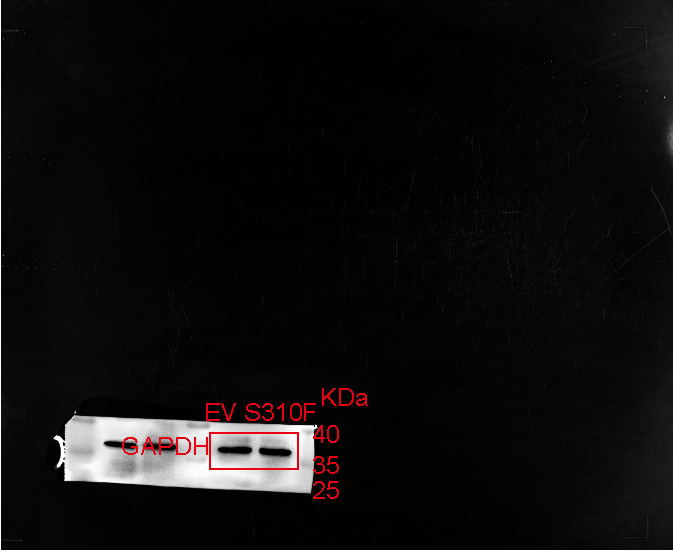

Supplement: Supplementary file 6 — Source data Fig. 3 [file 44321_2025_293_MOESM6_ESM.zip › Figure 3/3C/western GAPDH.tif]

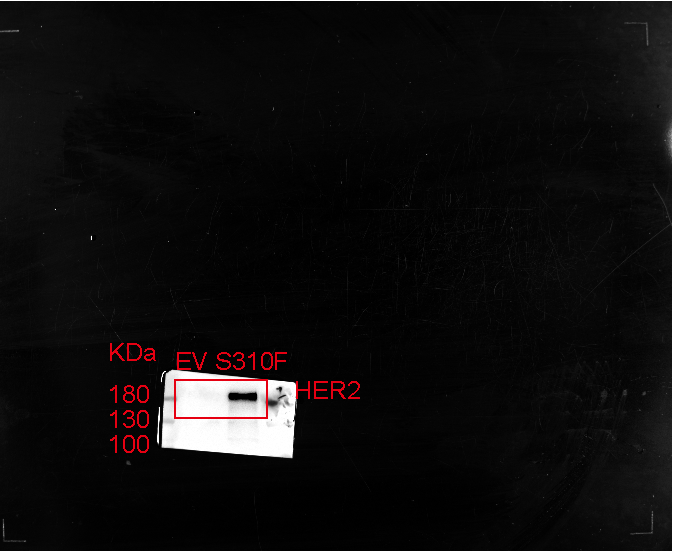

Supplement: Supplementary file 6 — Source data Fig. 3 [file 44321_2025_293_MOESM6_ESM.zip › Figure 3/3C/western HER2.tif]

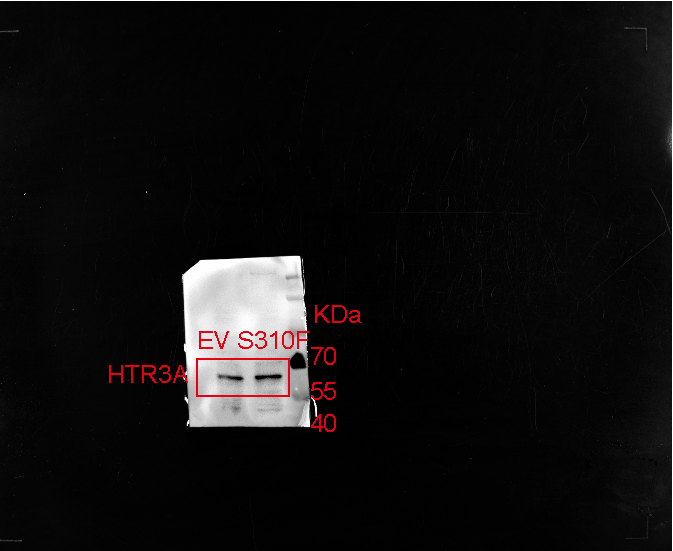

Supplement: Supplementary file 6 — Source data Fig. 3 [file 44321_2025_293_MOESM6_ESM.zip › Figure 3/3C/western HTR3A.tif]

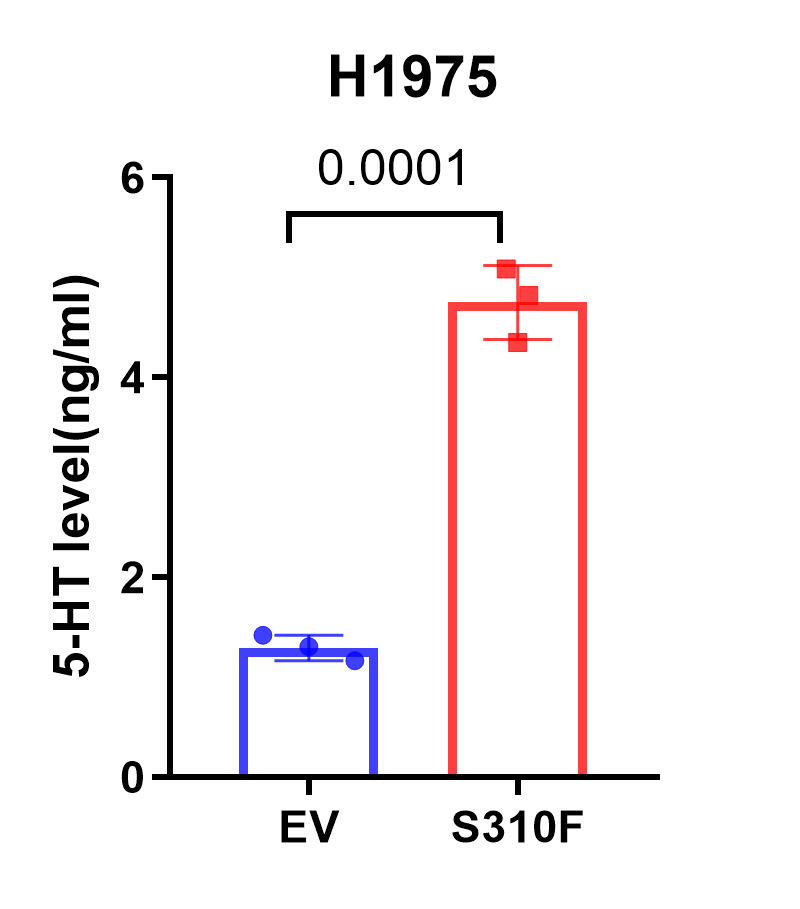

Supplement: Supplementary file 6 — Source data Fig. 3 [file 44321_2025_293_MOESM6_ESM.zip › Figure 3/3D-3F/Fig 3D.tif]

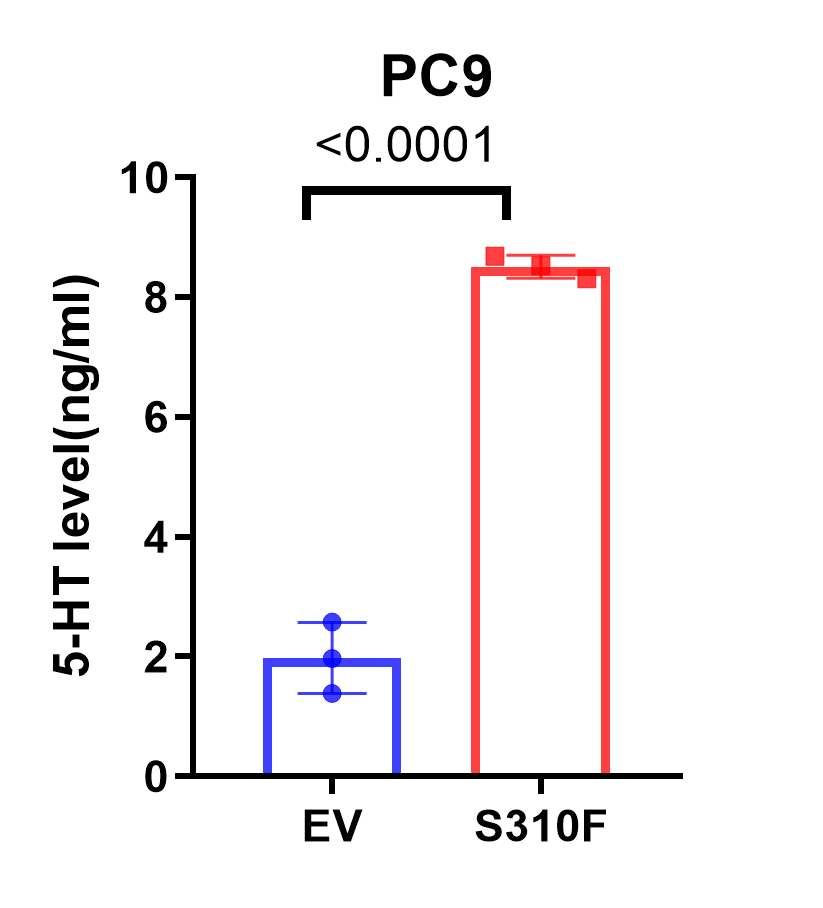

Supplement: Supplementary file 6 — Source data Fig. 3 [file 44321_2025_293_MOESM6_ESM.zip › Figure 3/3D-3F/Fig 3E.tif]

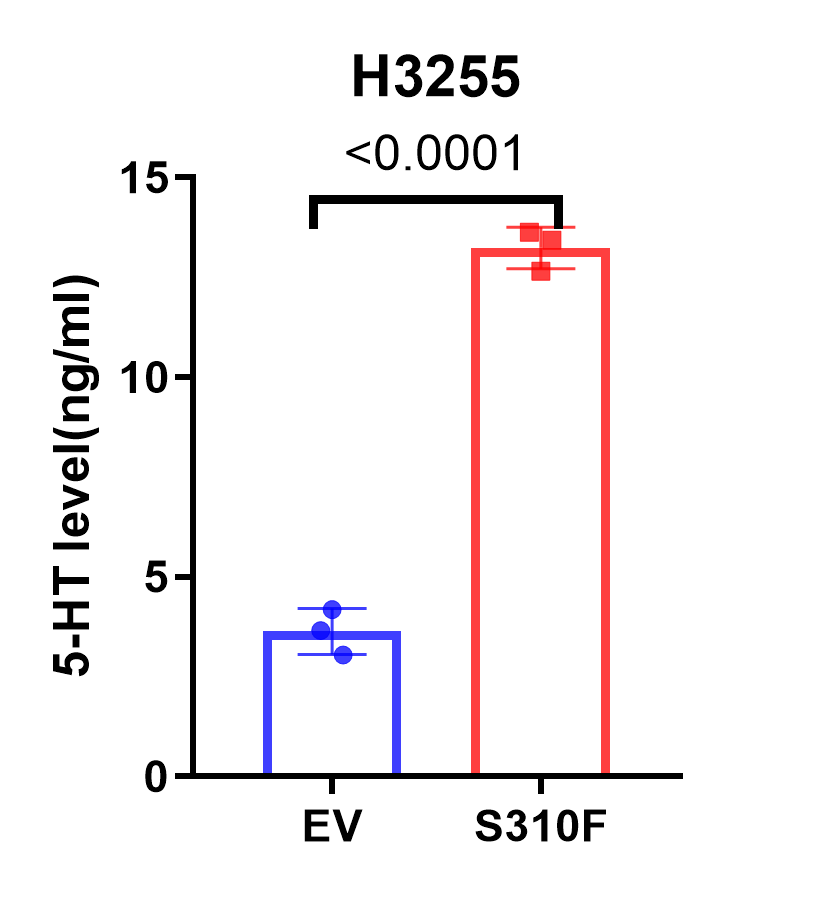

Supplement: Supplementary file 6 — Source data Fig. 3 [file 44321_2025_293_MOESM6_ESM.zip › Figure 3/3D-3F/Fig 3F.tif]

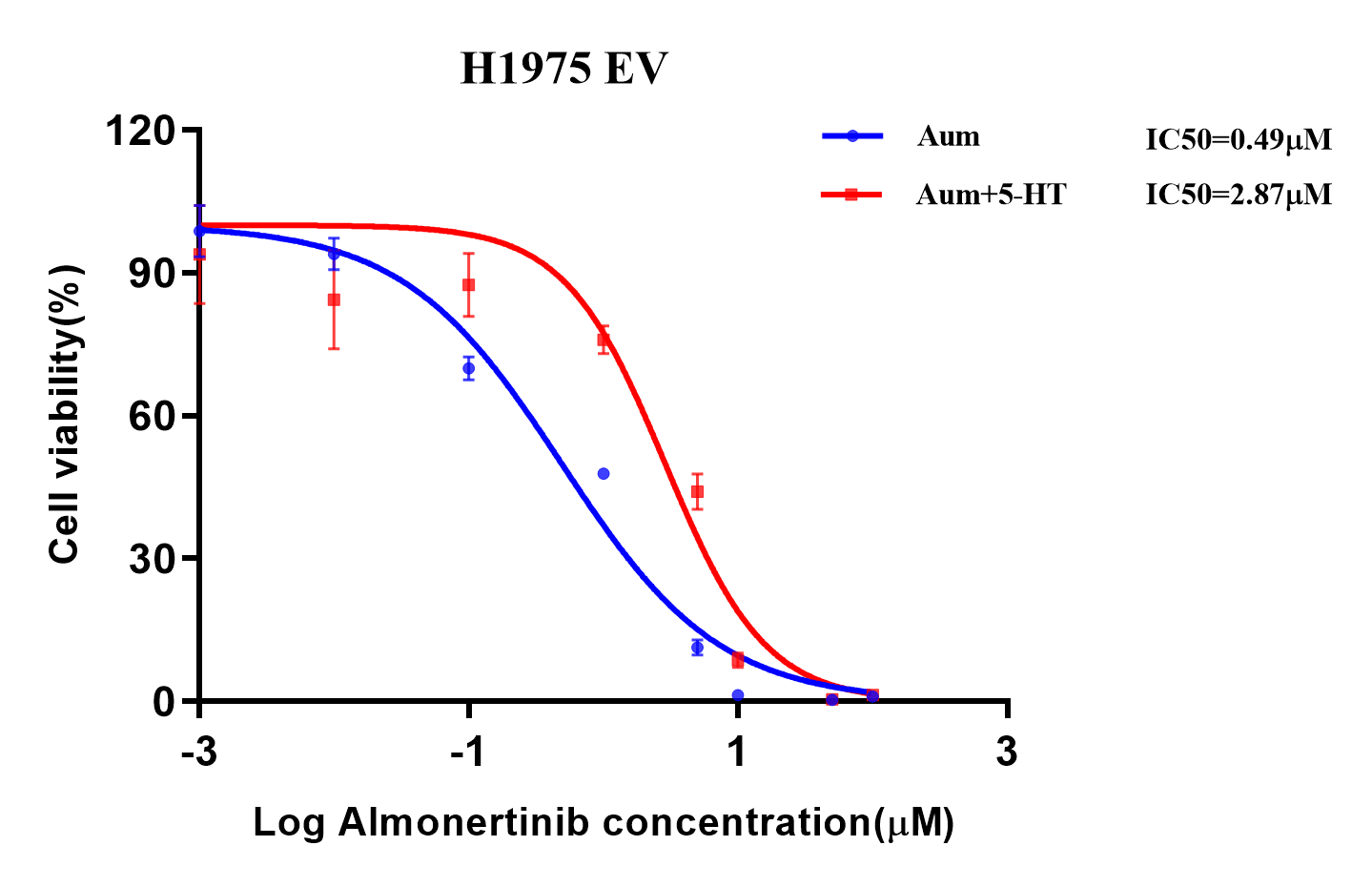

Supplement: Supplementary file 6 — Source data Fig. 3 [file 44321_2025_293_MOESM6_ESM.zip › Figure 3/3G-3I/Fig 3G.tif]

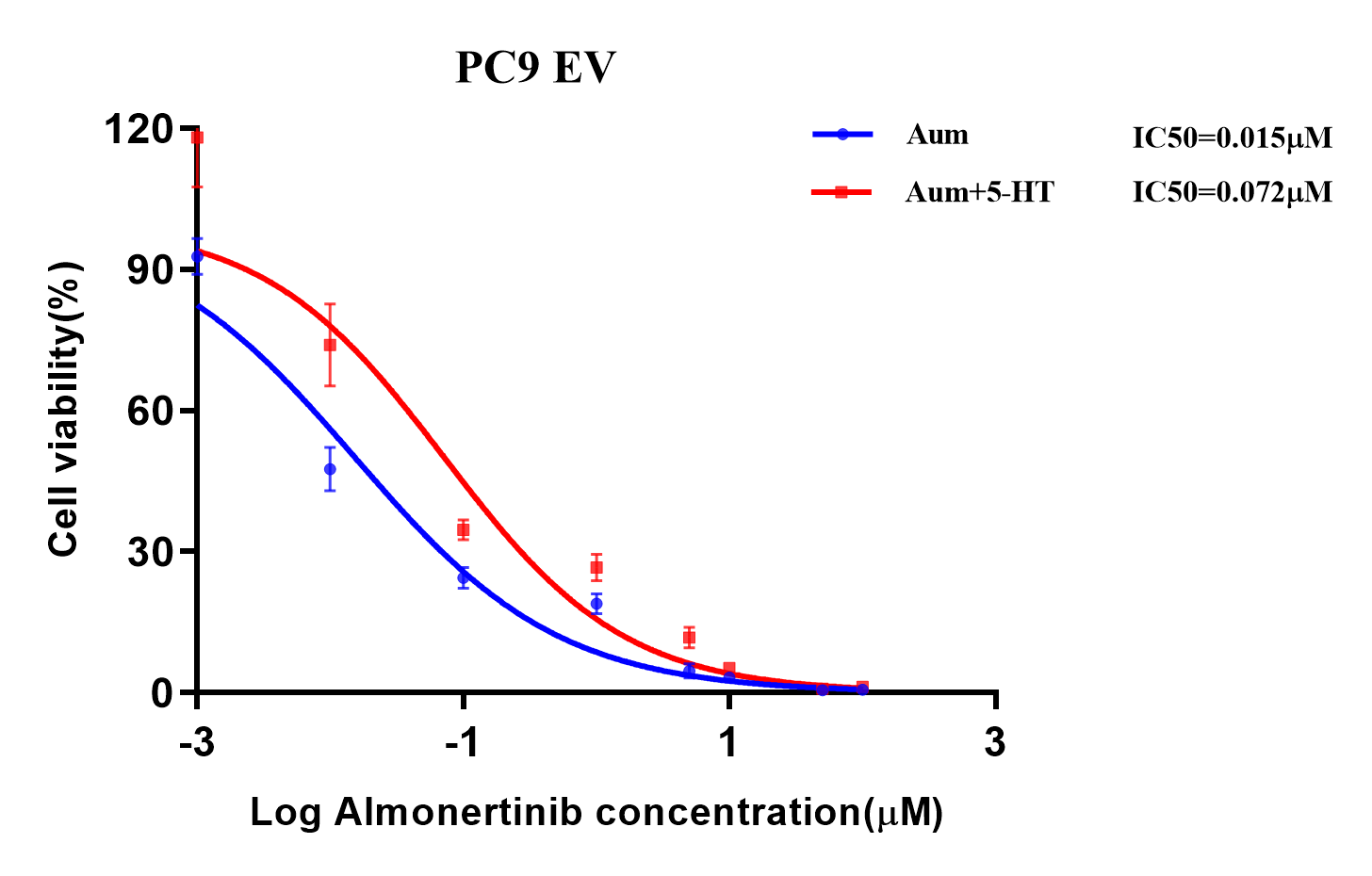

Supplement: Supplementary file 6 — Source data Fig. 3 [file 44321_2025_293_MOESM6_ESM.zip › Figure 3/3G-3I/Fig 3H.tif]

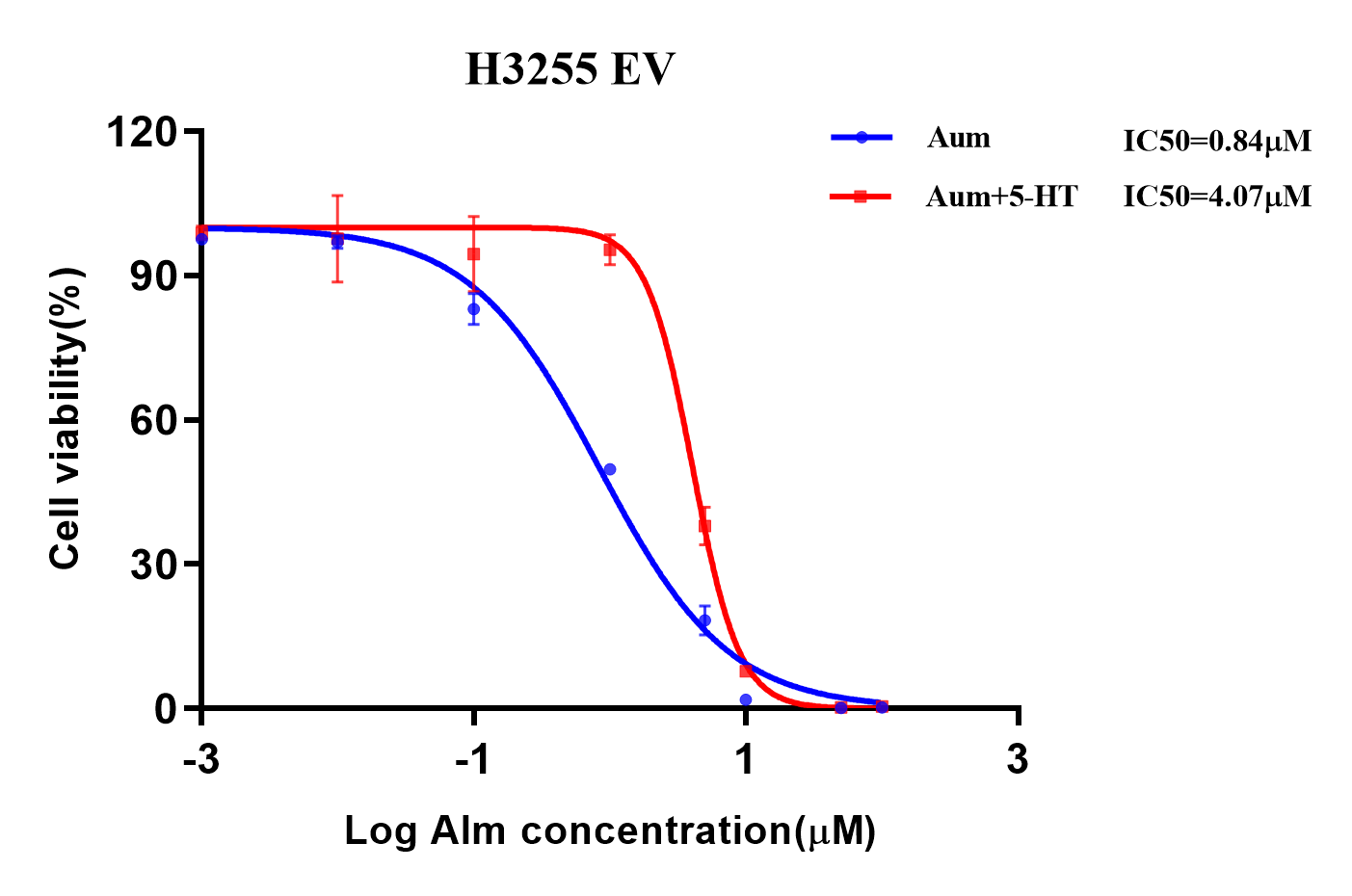

Supplement: Supplementary file 6 — Source data Fig. 3 [file 44321_2025_293_MOESM6_ESM.zip › Figure 3/3G-3I/Fig 3I.tif]

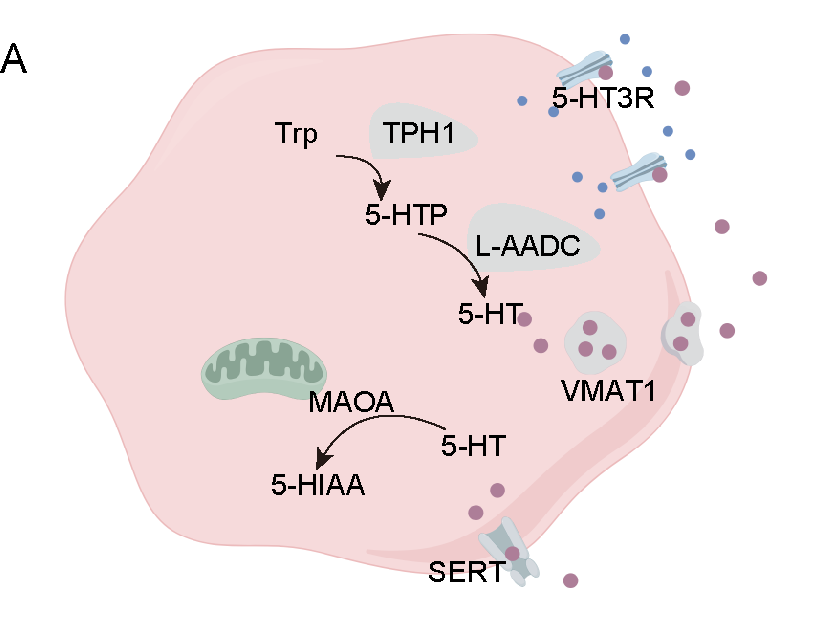

Supplement: Supplementary file 7 — Source data Fig. 4 [file 44321_2025_293_MOESM7_ESM.zip › Figure 4/4A/4A.tif]

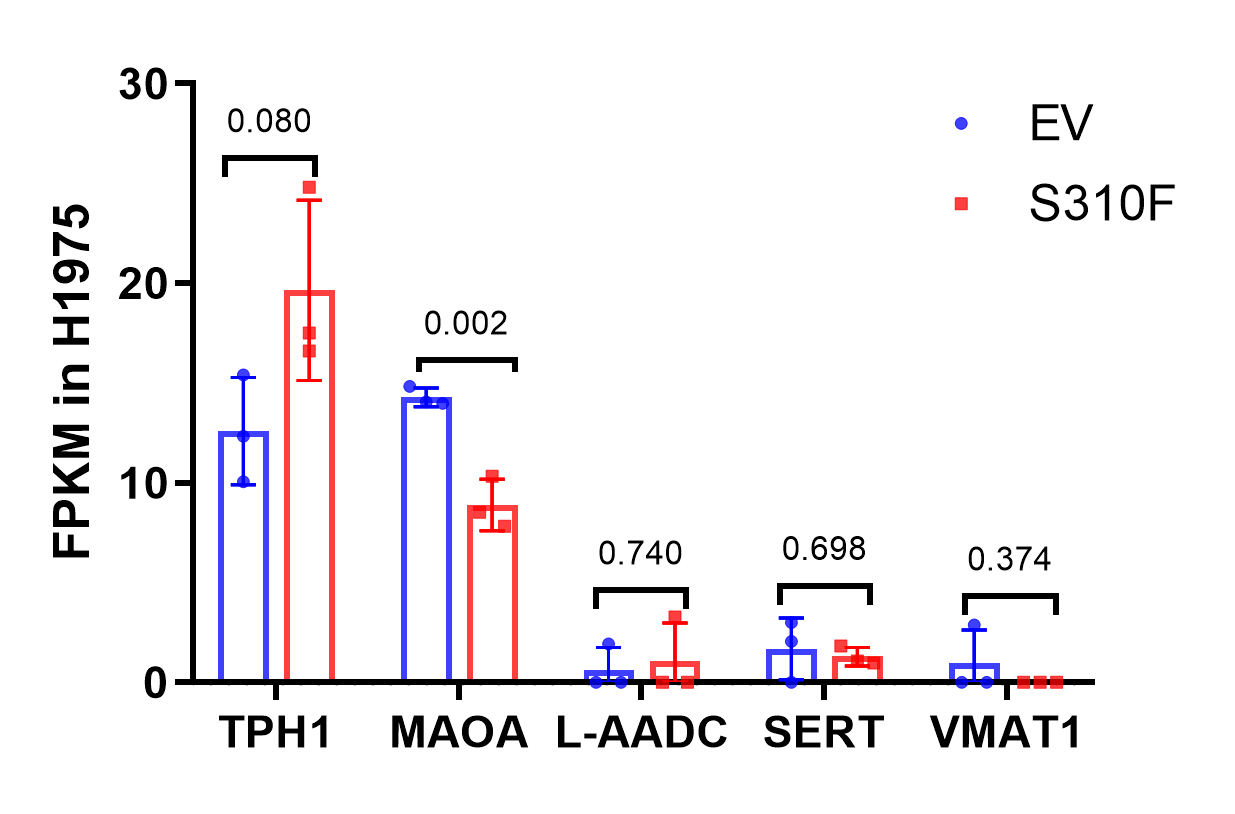

Supplement: Supplementary file 7 — Source data Fig. 4 [file 44321_2025_293_MOESM7_ESM.zip › Figure 4/4B/Fig 4B.tif]

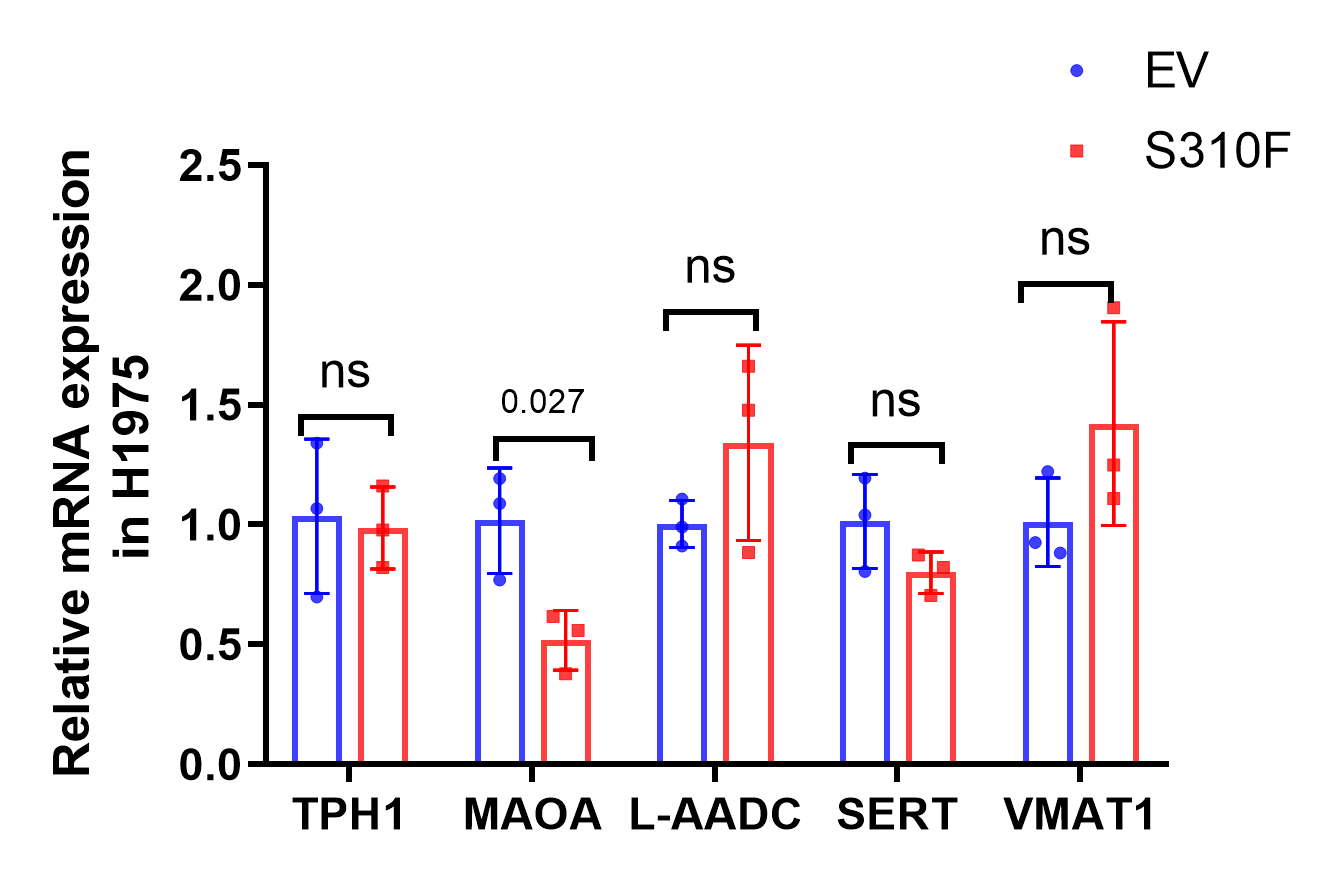

Supplement: Supplementary file 7 — Source data Fig. 4 [file 44321_2025_293_MOESM7_ESM.zip › Figure 4/4C-4D/Fig 4C.tif]

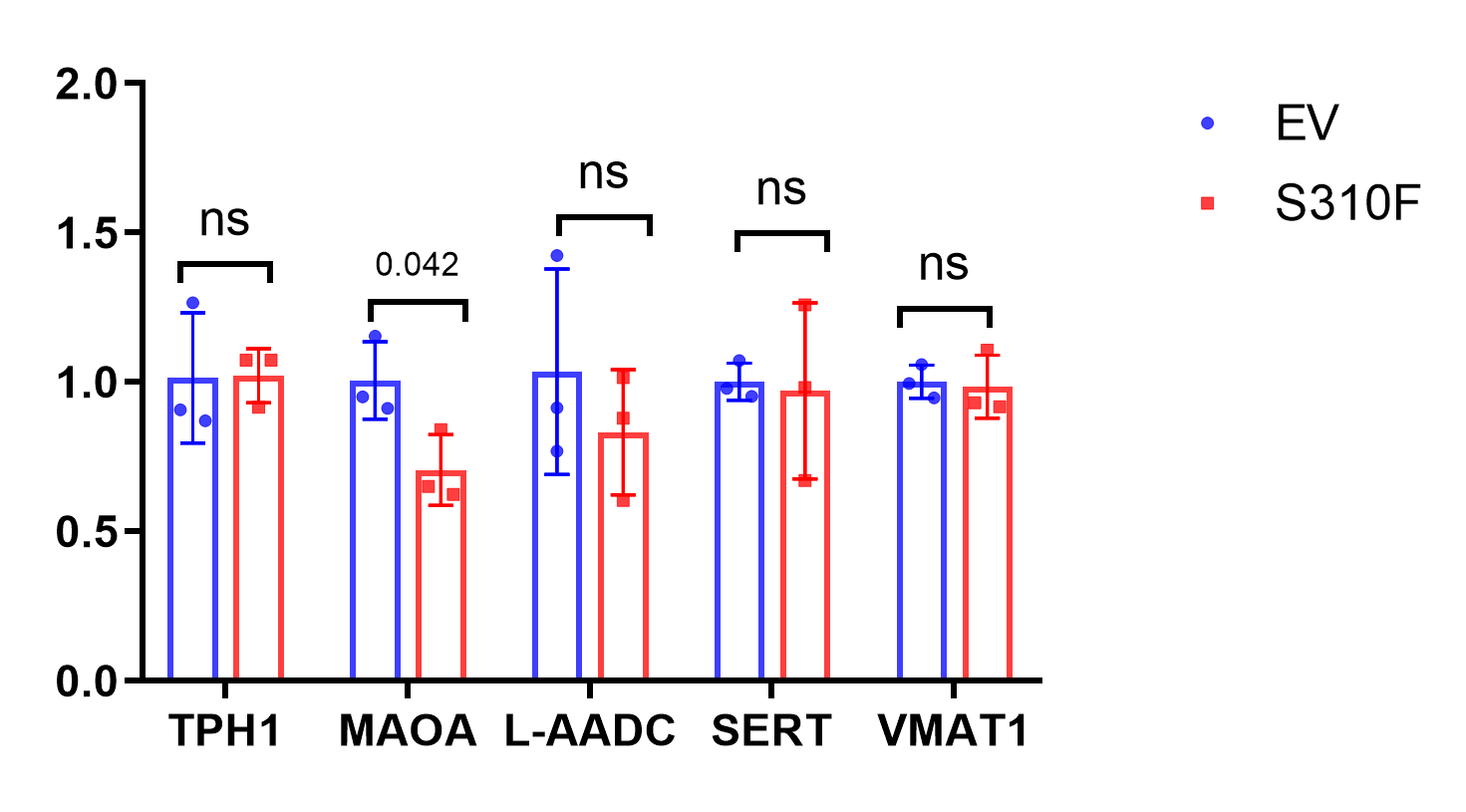

Supplement: Supplementary file 7 — Source data Fig. 4 [file 44321_2025_293_MOESM7_ESM.zip › Figure 4/4C-4D/Fig 4D.tif]

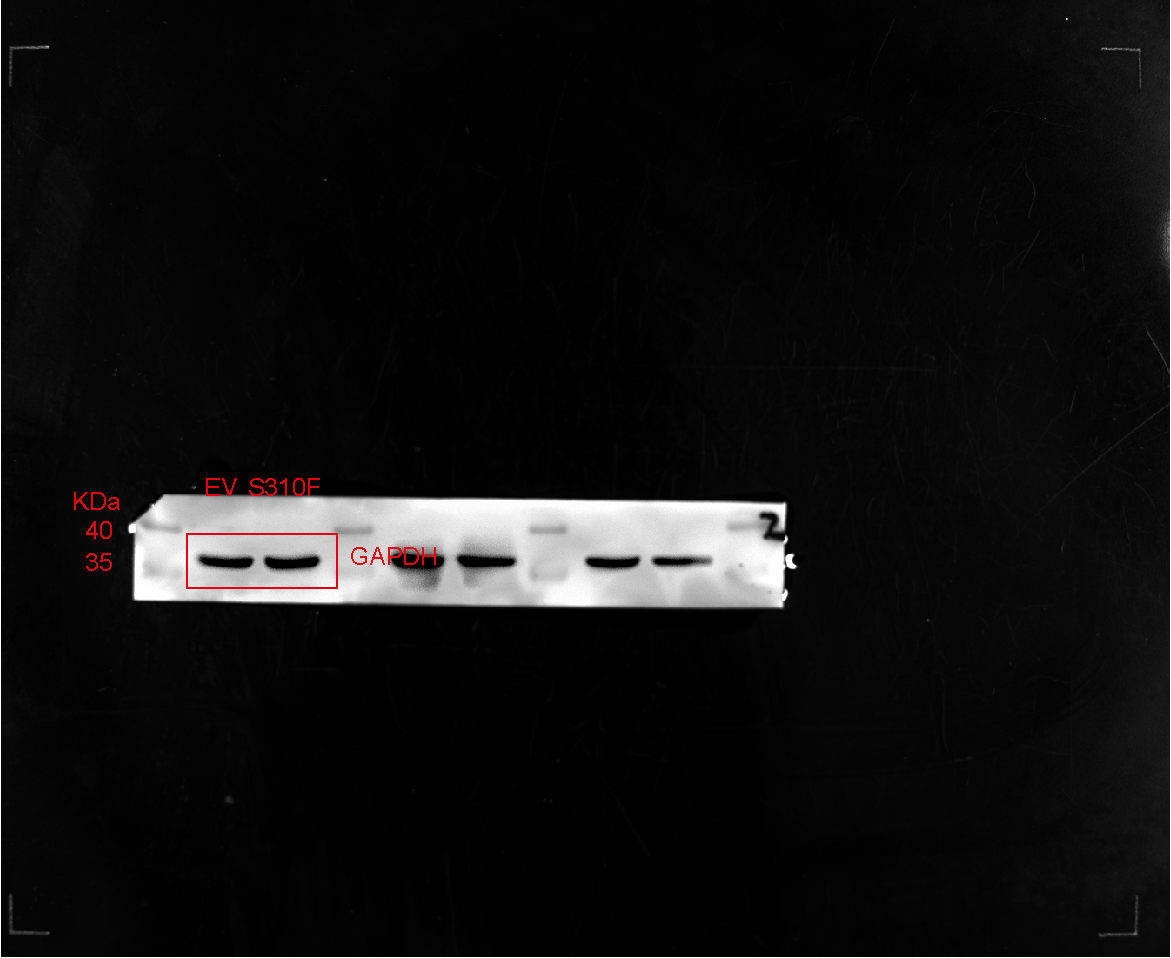

Supplement: Supplementary file 7 — Source data Fig. 4 [file 44321_2025_293_MOESM7_ESM.zip › Figure 4/4E/western H1975-GAPDH.tif]

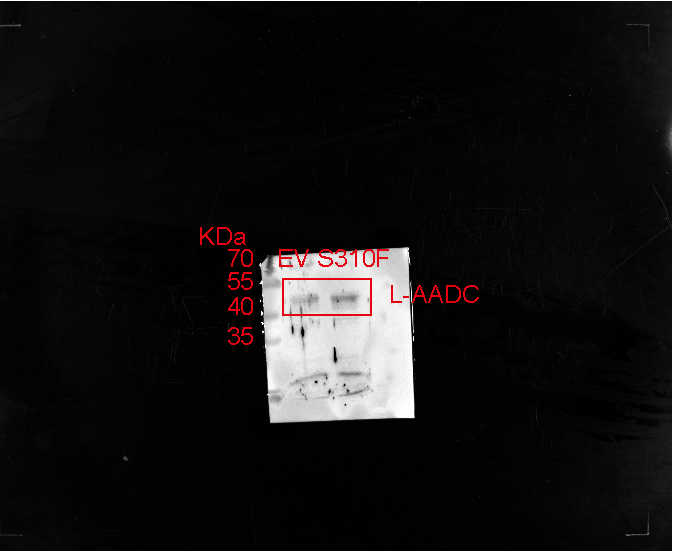

Supplement: Supplementary file 7 — Source data Fig. 4 [file 44321_2025_293_MOESM7_ESM.zip › Figure 4/4E/western H1975-L-AADC.tif]

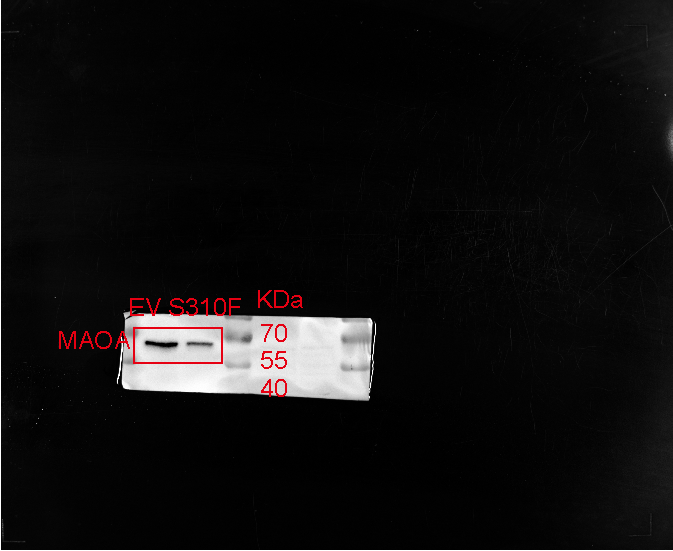

Supplement: Supplementary file 7 — Source data Fig. 4 [file 44321_2025_293_MOESM7_ESM.zip › Figure 4/4E/western H1975-MAOA.tif]

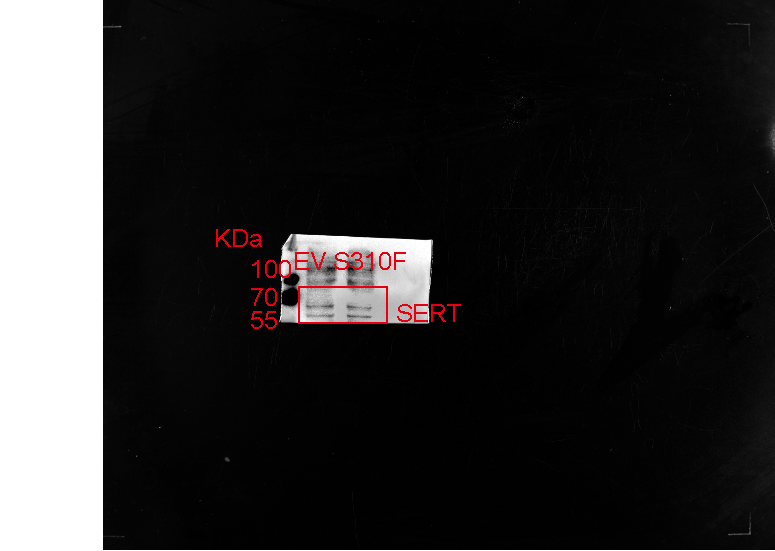

Supplement: Supplementary file 7 — Source data Fig. 4 [file 44321_2025_293_MOESM7_ESM.zip › Figure 4/4E/western H1975-SERT.tif]

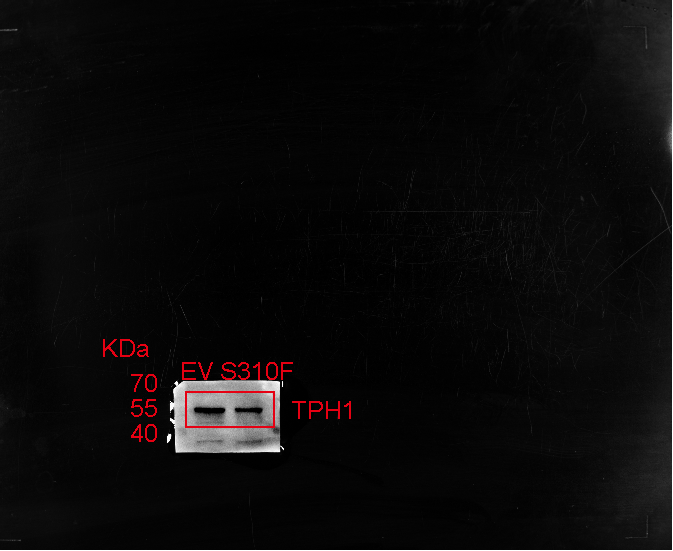

Supplement: Supplementary file 7 — Source data Fig. 4 [file 44321_2025_293_MOESM7_ESM.zip › Figure 4/4E/western H1975-TPH1.tif]

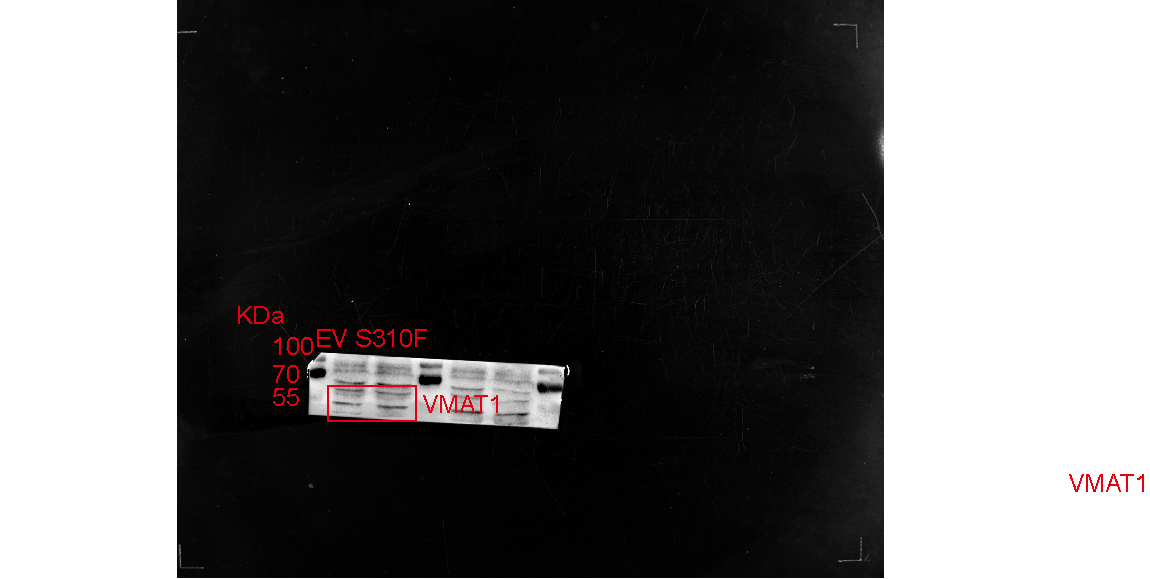

Supplement: Supplementary file 7 — Source data Fig. 4 [file 44321_2025_293_MOESM7_ESM.zip › Figure 4/4E/western H1975-VMAT1.tif]

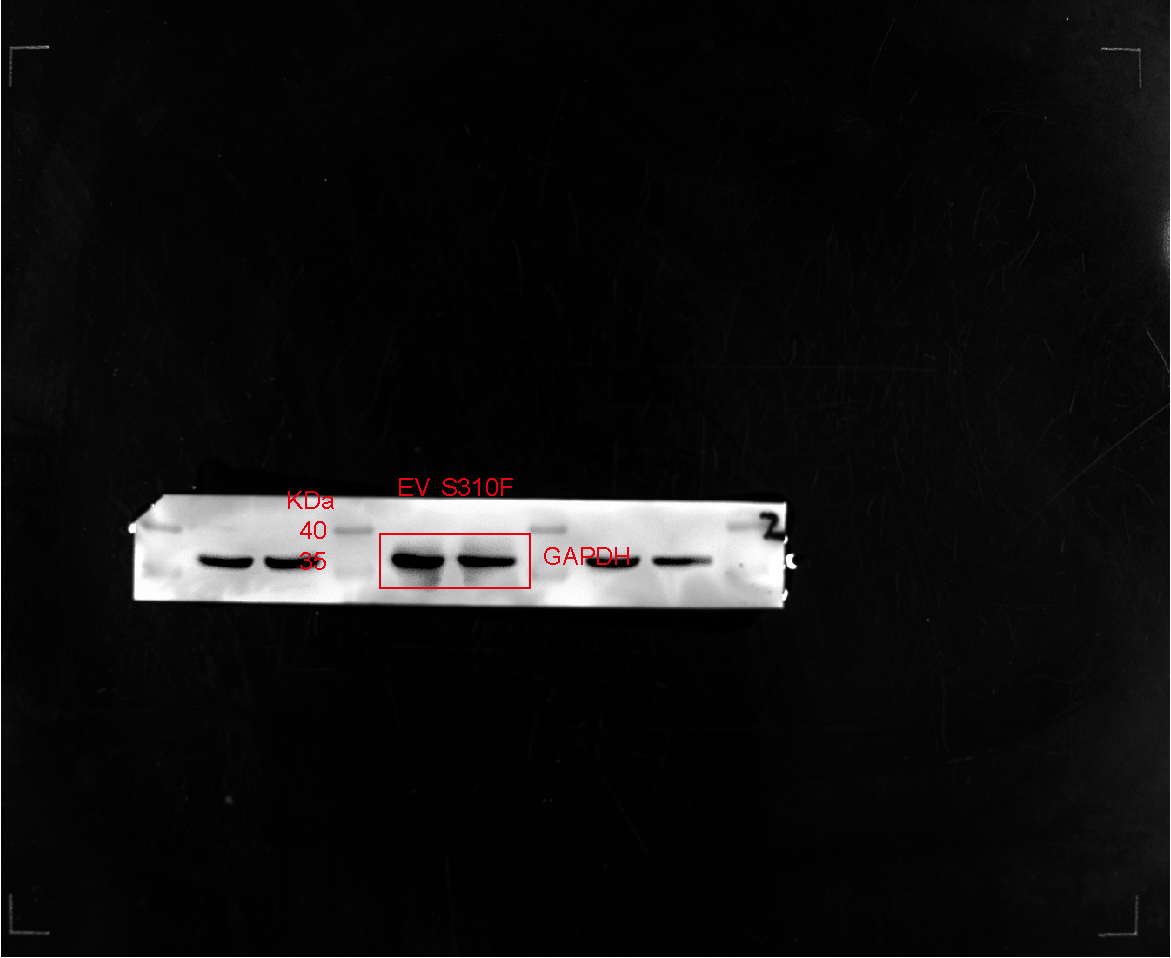

Supplement: Supplementary file 7 — Source data Fig. 4 [file 44321_2025_293_MOESM7_ESM.zip › Figure 4/4E/western PC9-GAPDH.tif]

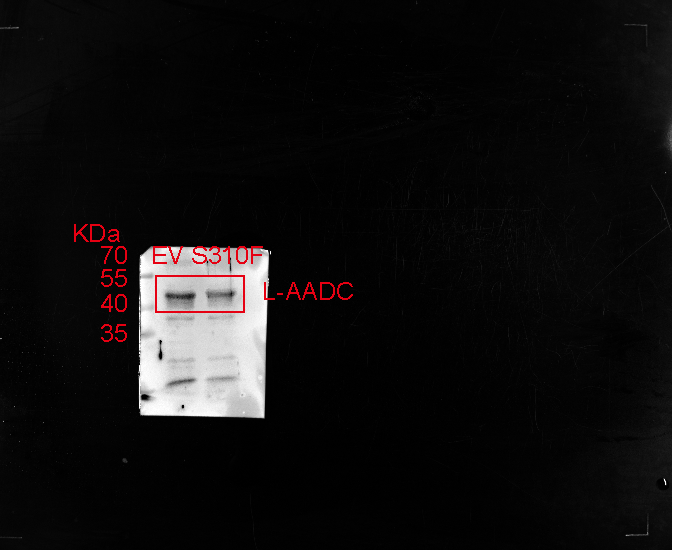

Supplement: Supplementary file 7 — Source data Fig. 4 [file 44321_2025_293_MOESM7_ESM.zip › Figure 4/4E/western PC9-L-AADC.tif]

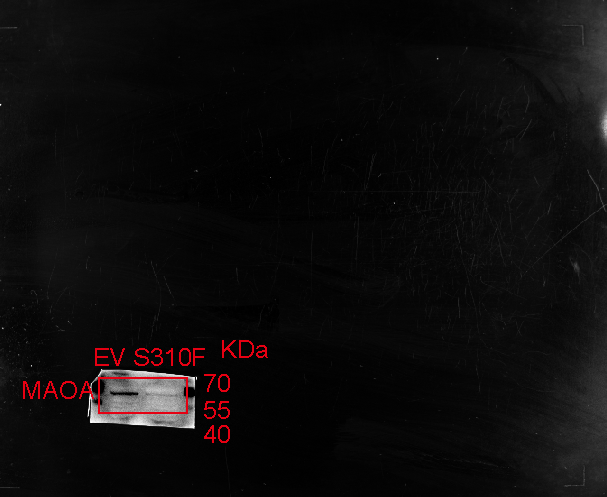

Supplement: Supplementary file 7 — Source data Fig. 4 [file 44321_2025_293_MOESM7_ESM.zip › Figure 4/4E/western PC9-MAOA.tif]

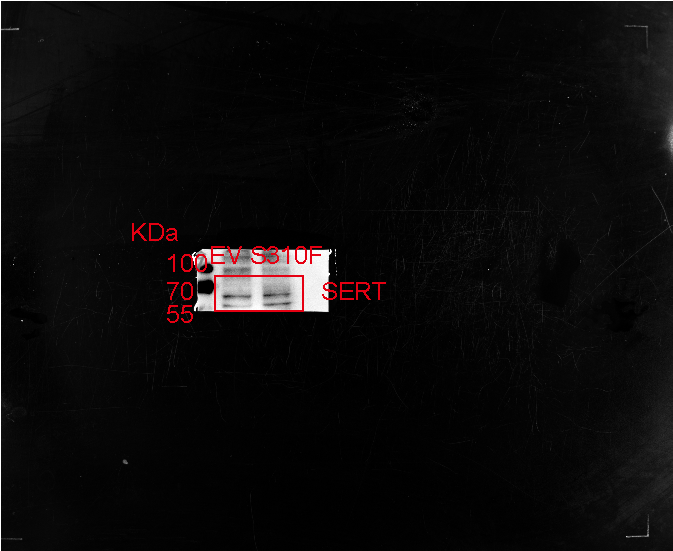

Supplement: Supplementary file 7 — Source data Fig. 4 [file 44321_2025_293_MOESM7_ESM.zip › Figure 4/4E/western PC9-SERT.tif]

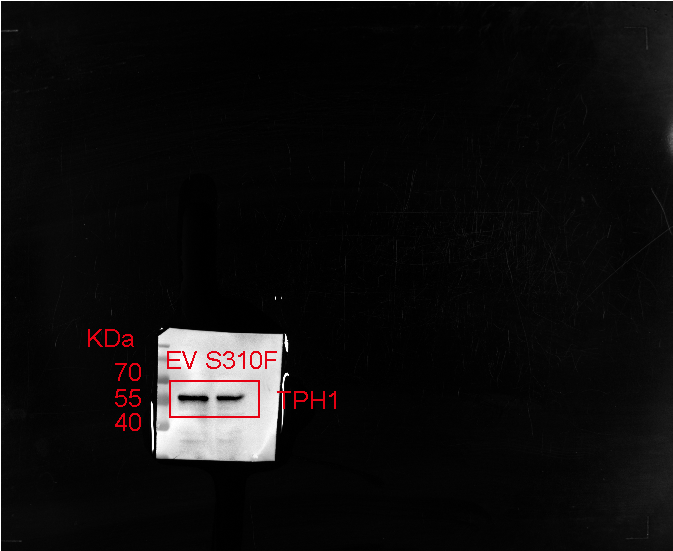

Supplement: Supplementary file 7 — Source data Fig. 4 [file 44321_2025_293_MOESM7_ESM.zip › Figure 4/4E/western PC9-TPH1.tif]

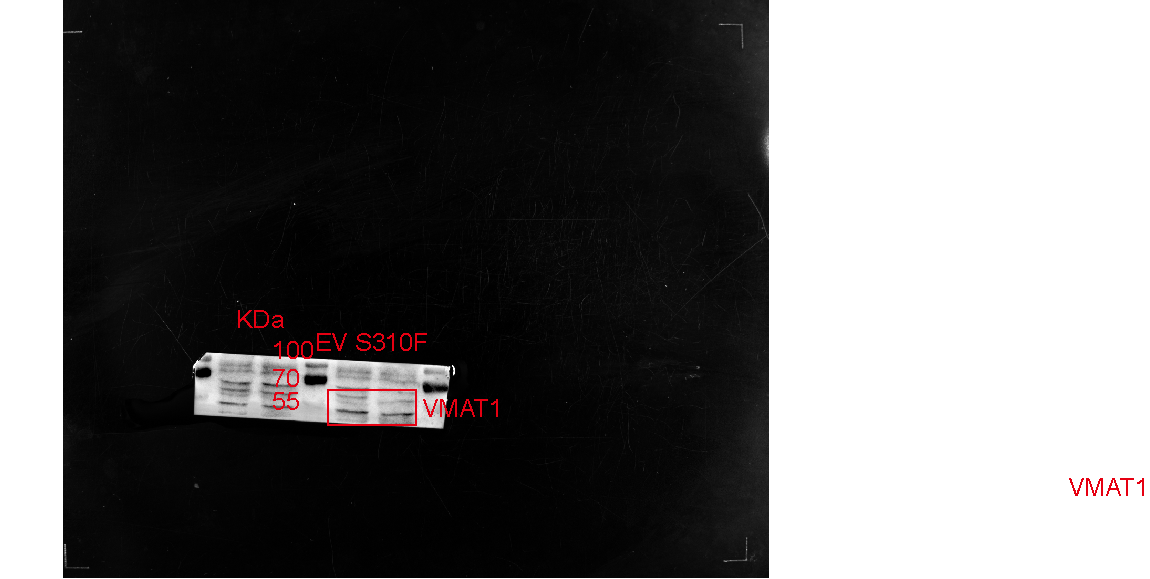

Supplement: Supplementary file 7 — Source data Fig. 4 [file 44321_2025_293_MOESM7_ESM.zip › Figure 4/4E/western PC9-VMAT1.tif]

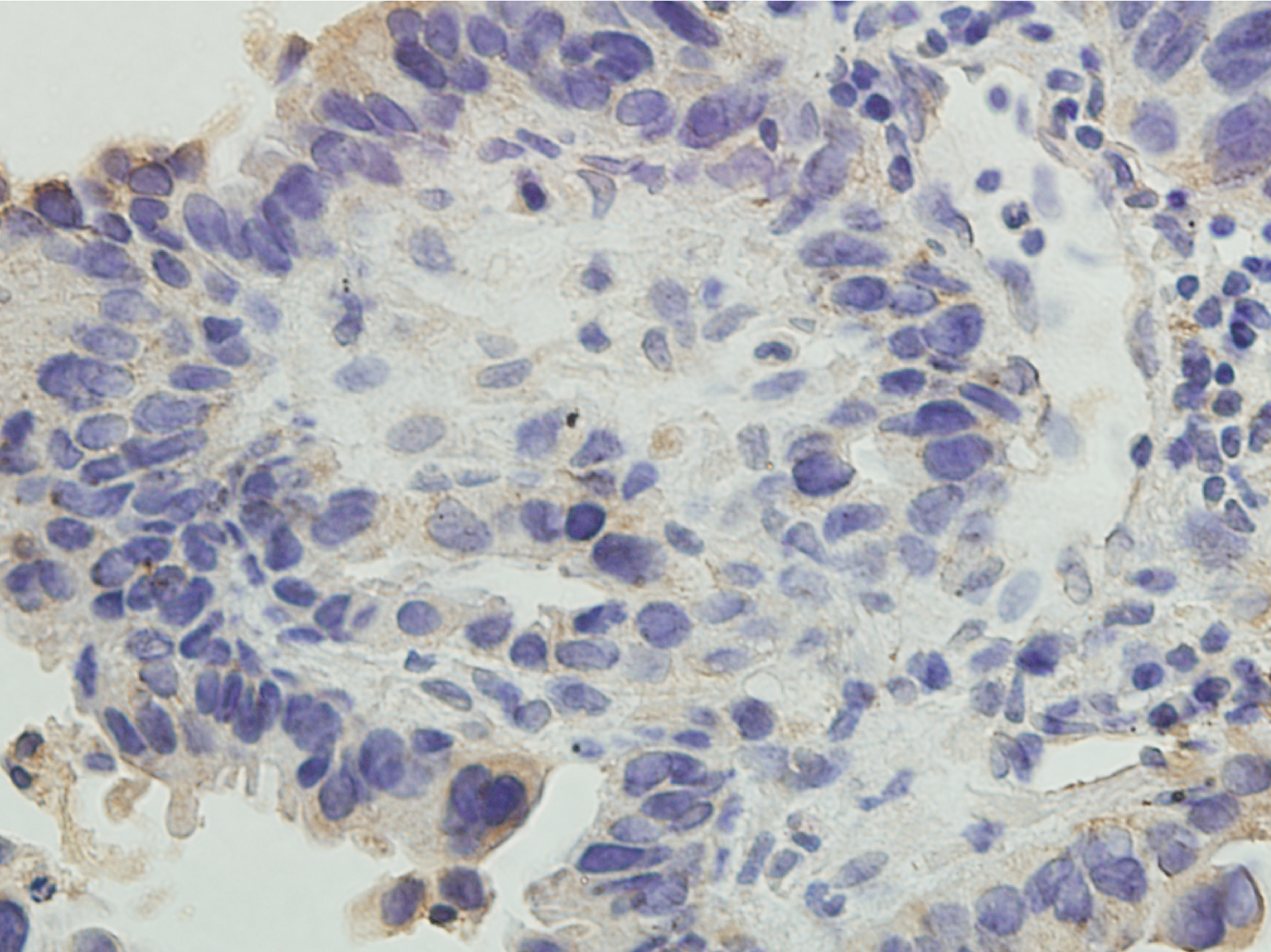

Supplement: Supplementary file 7 — Source data Fig. 4 [file 44321_2025_293_MOESM7_ESM.zip › Figure 4/4F/case1-EGFR&HER2.tif]

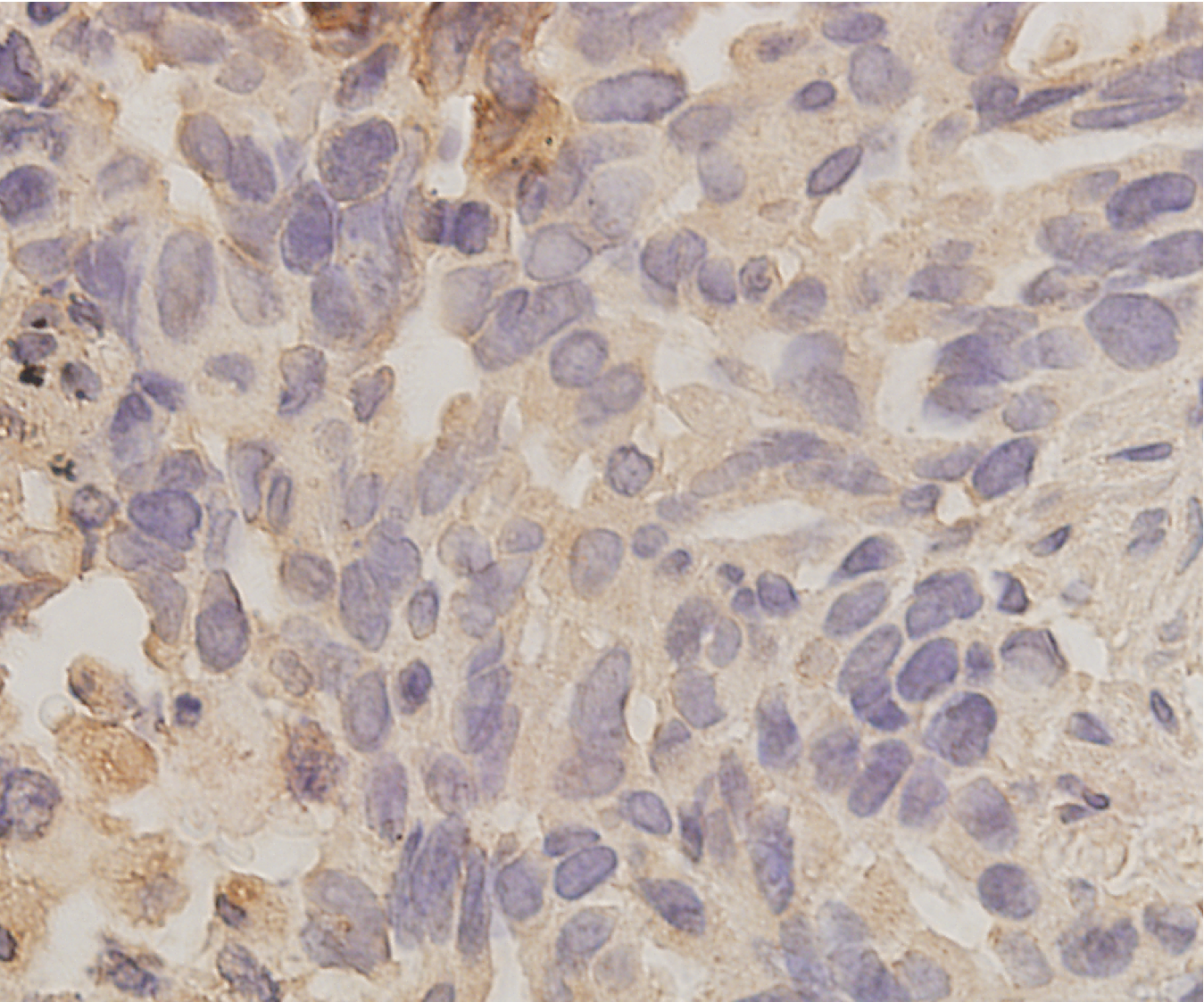

Supplement: Supplementary file 7 — Source data Fig. 4 [file 44321_2025_293_MOESM7_ESM.zip › Figure 4/4F/case1-EGFR.tif]

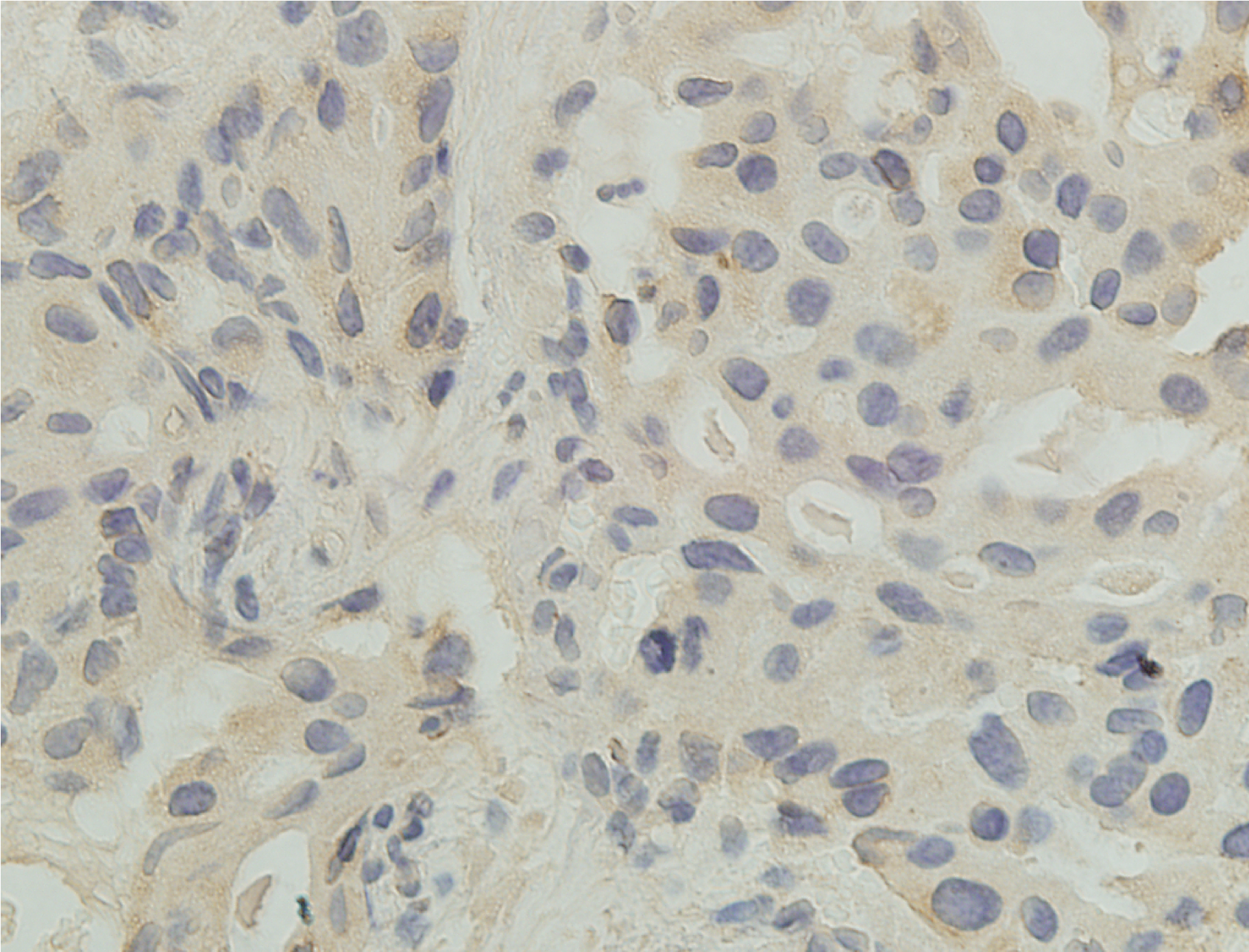

Supplement: Supplementary file 7 — Source data Fig. 4 [file 44321_2025_293_MOESM7_ESM.zip › Figure 4/4F/case2-EGFR&HER2.tif]

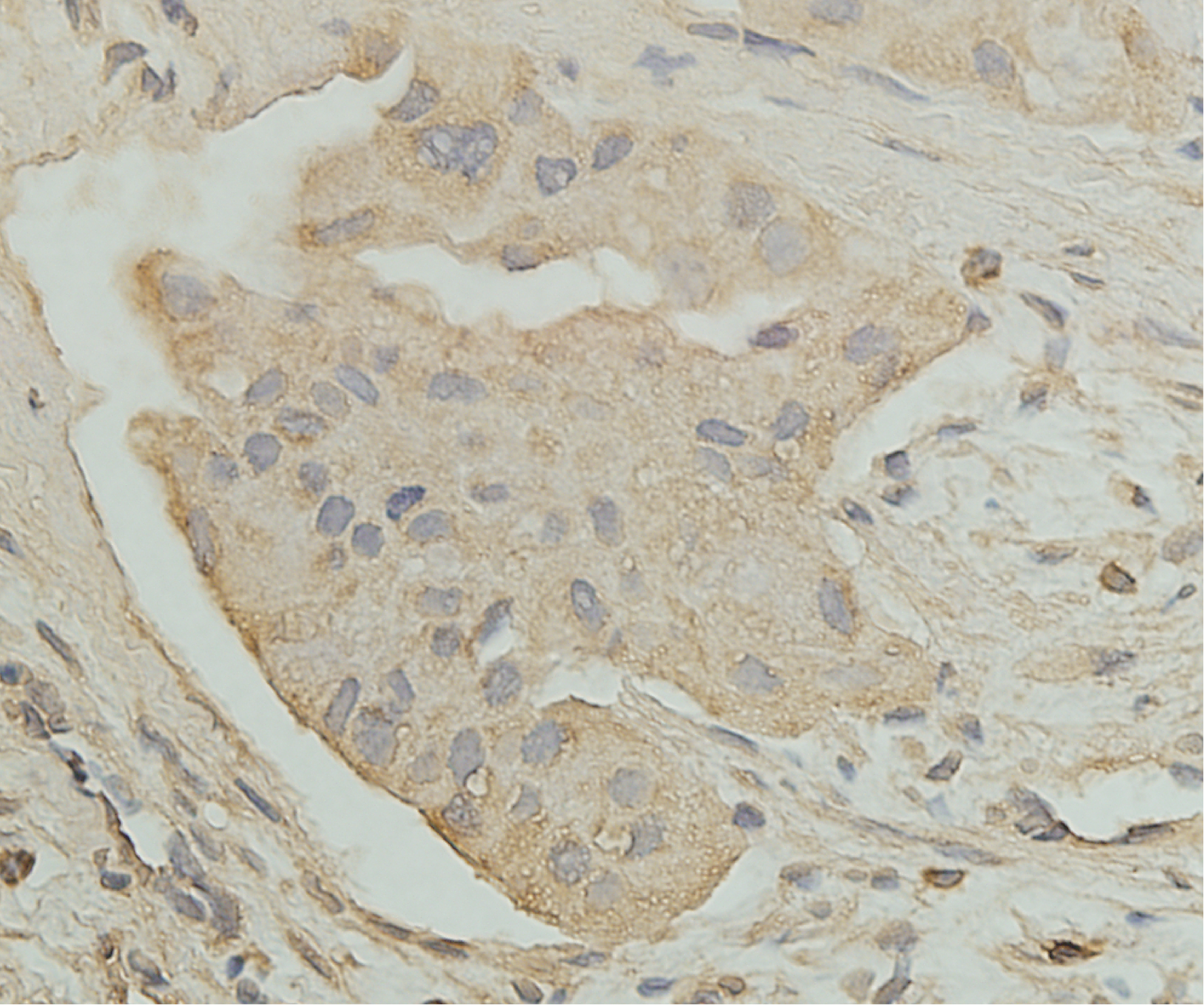

Supplement: Supplementary file 7 — Source data Fig. 4 [file 44321_2025_293_MOESM7_ESM.zip › Figure 4/4F/case2-EGFR.tif]

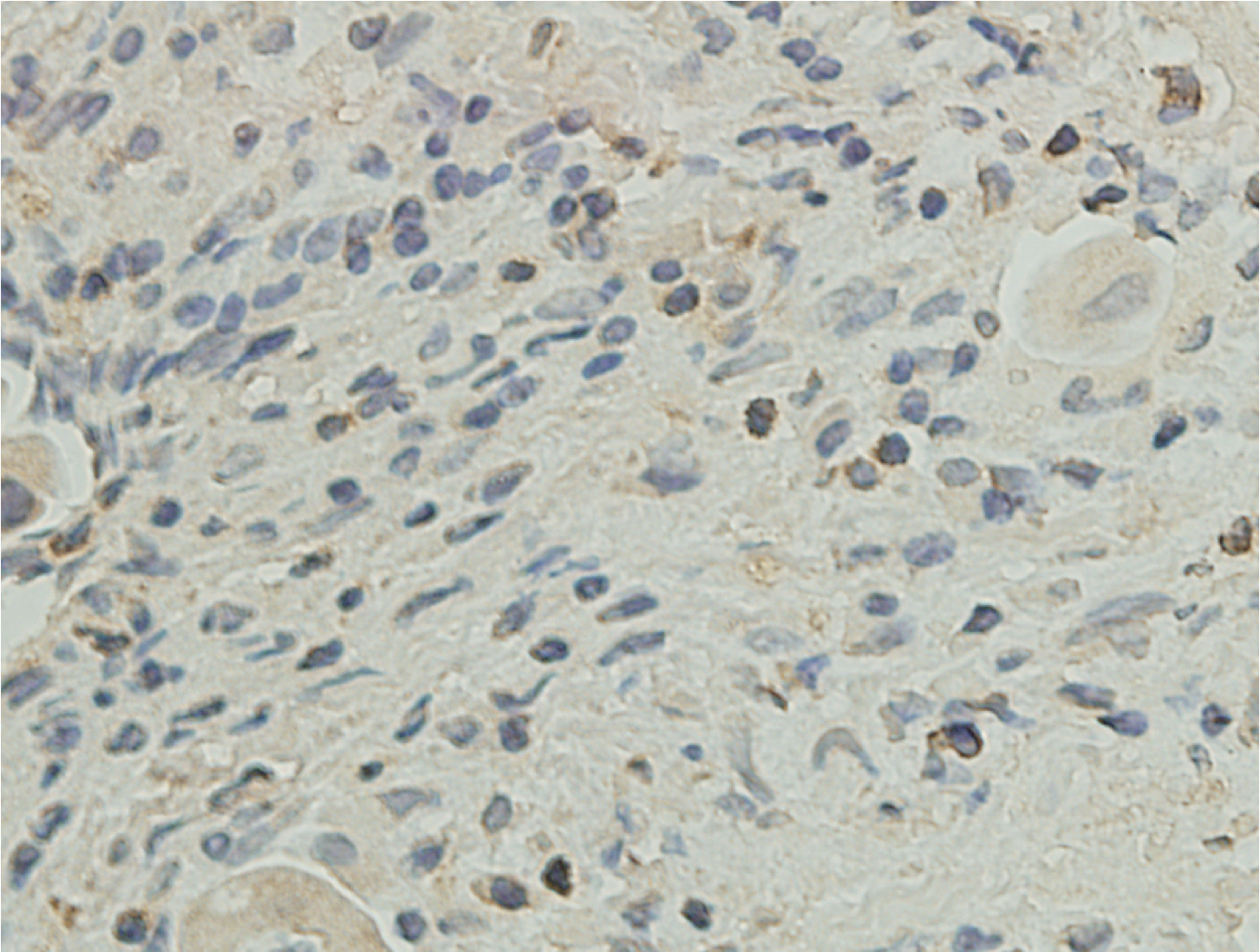

Supplement: Supplementary file 7 — Source data Fig. 4 [file 44321_2025_293_MOESM7_ESM.zip › Figure 4/4F/case3-EGFR&HER2.tif]

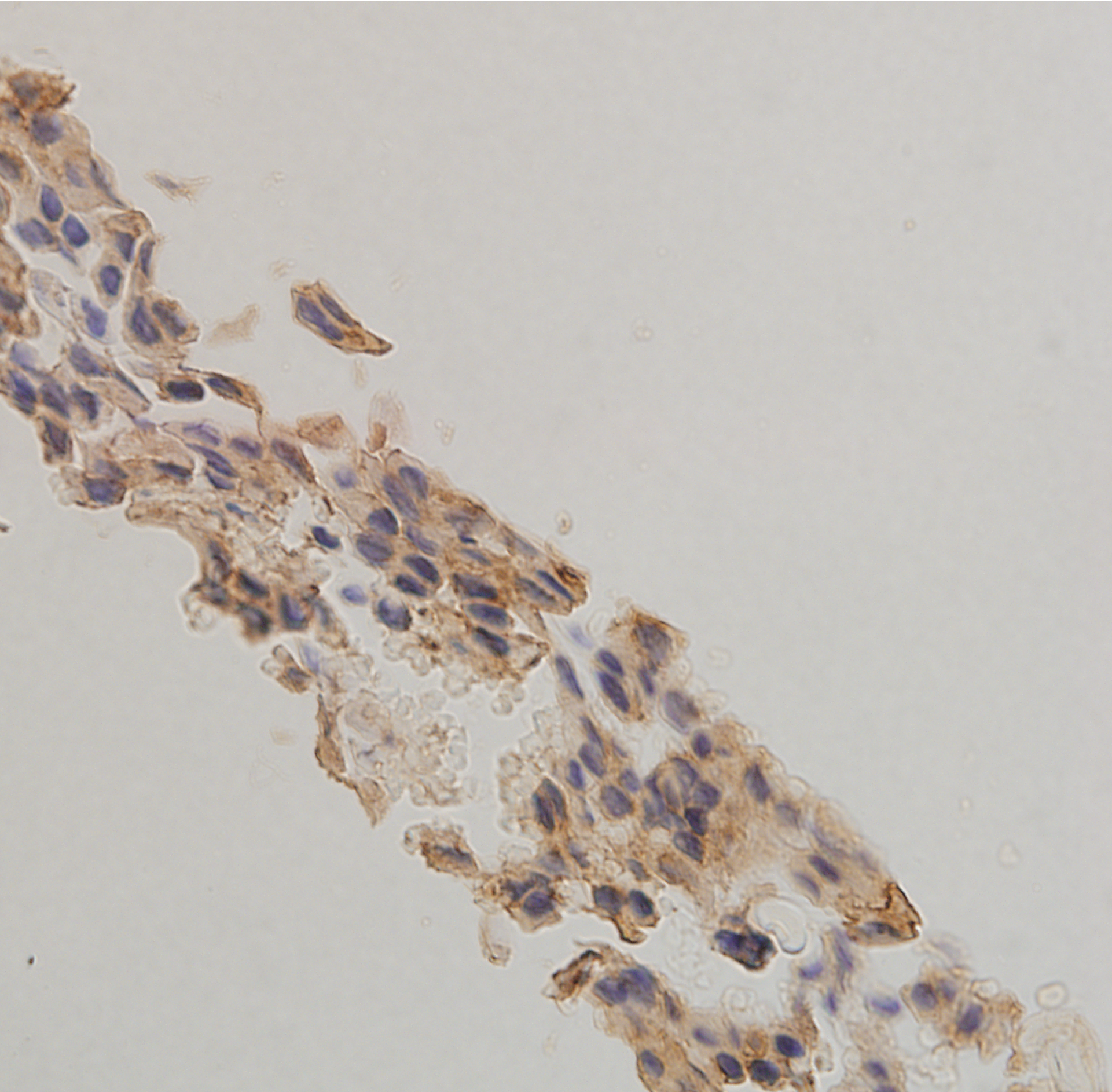

Supplement: Supplementary file 7 — Source data Fig. 4 [file 44321_2025_293_MOESM7_ESM.zip › Figure 4/4F/case3-EGFR.tif]

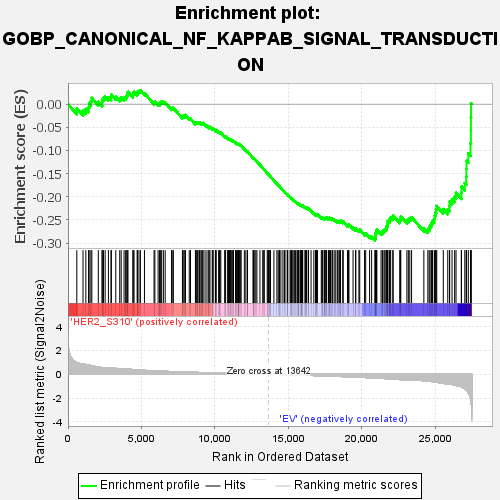

Supplement: Supplementary file 8 — Source data Fig. 5 [file 44321_2025_293_MOESM8_ESM.zip › Figure 5/5A/NF-KB.png]

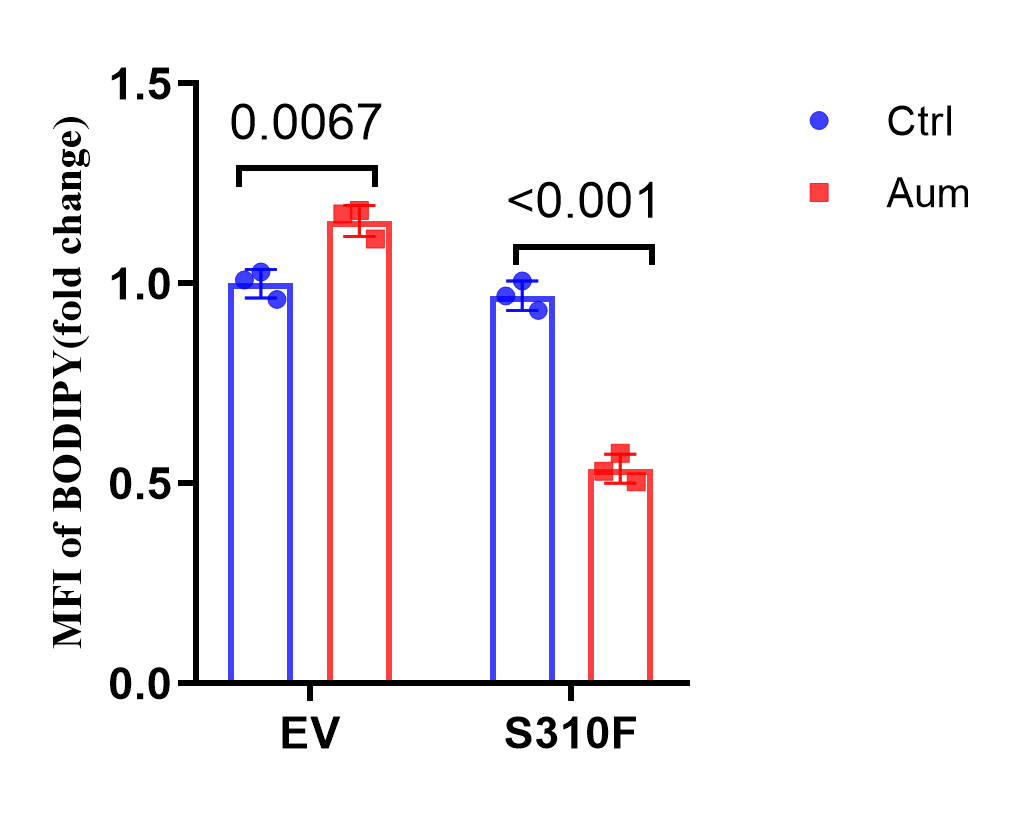

Supplement: Supplementary file 8 — Source data Fig. 5 [file 44321_2025_293_MOESM8_ESM.zip › Figure 5/5B/Fig 5B H1975.tif]

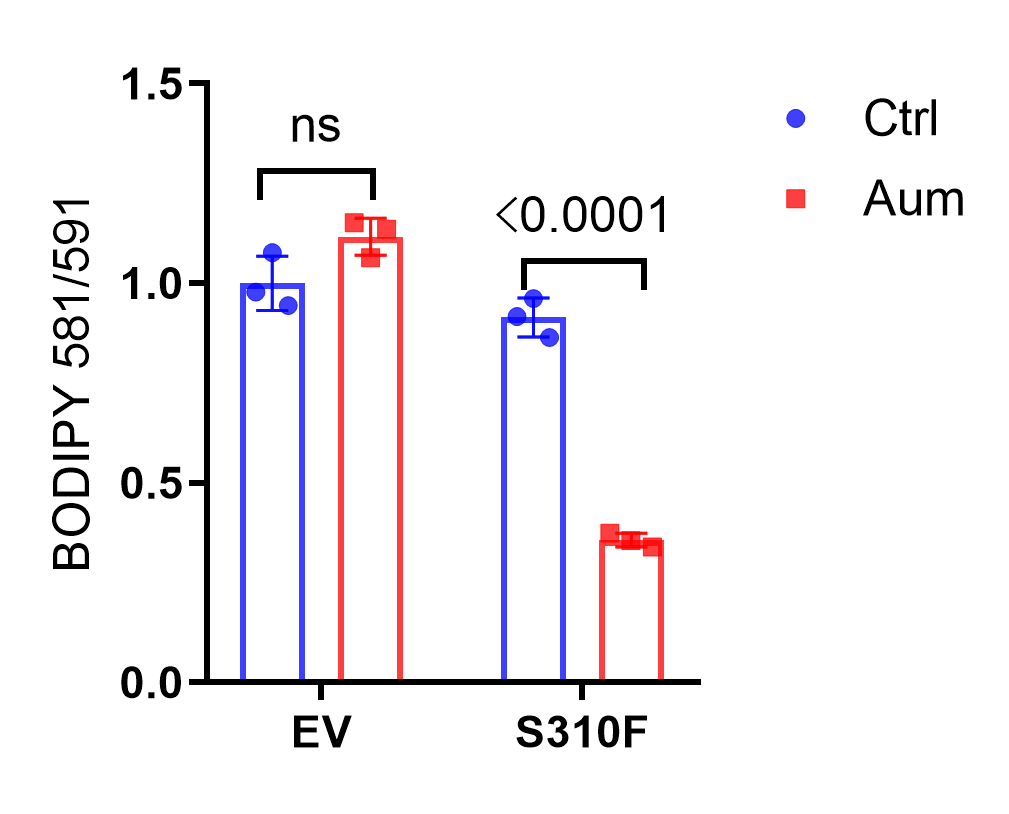

Supplement: Supplementary file 8 — Source data Fig. 5 [file 44321_2025_293_MOESM8_ESM.zip › Figure 5/5B/Fig 5B PC9.tif]

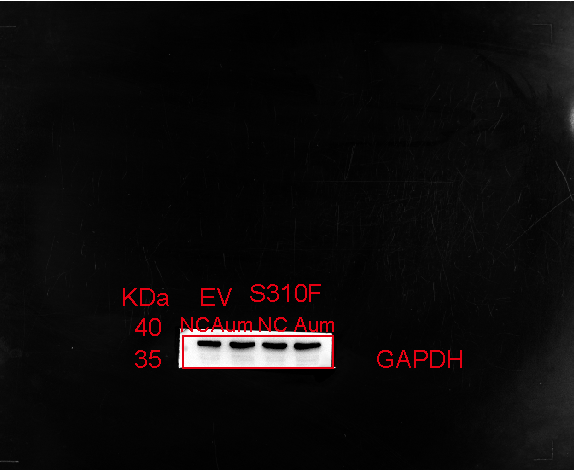

Supplement: Supplementary file 8 — Source data Fig. 5 [file 44321_2025_293_MOESM8_ESM.zip › Figure 5/5C/western GAPDH.tif]
