# Supplementary figures and images for: 5-HT regulates resistance to aumolertinib by attenuating ferroptosis in lung adenocarcinoma (part 2 of 2)
Source: EMBO Mol Med. 2025 Sep 2;17(10):2586–611. doi: 10.1038/s44321-025-00293-5 (PMC12514003; doi:10.1038/s44321-025-00293-5)

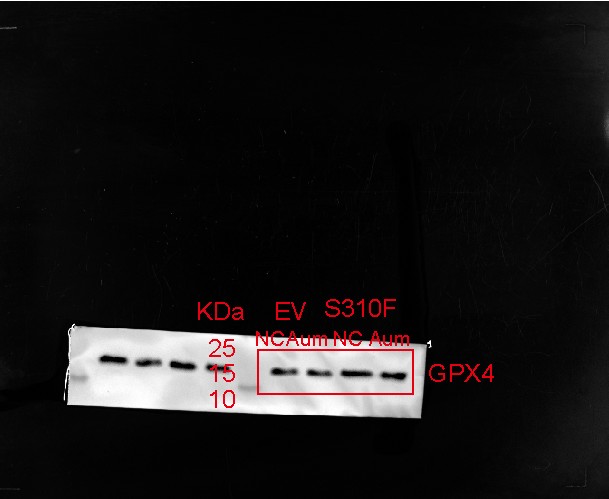

Supplement: Supplementary file 8 — Source data Fig. 5 [file 44321_2025_293_MOESM8_ESM.zip › Figure 5/5C/western GPX4.tif]

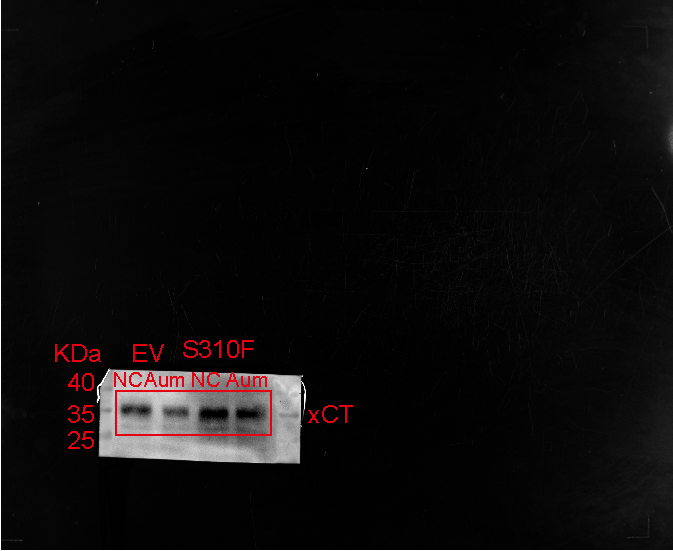

Supplement: Supplementary file 8 — Source data Fig. 5 [file 44321_2025_293_MOESM8_ESM.zip › Figure 5/5C/western xCT.tif]

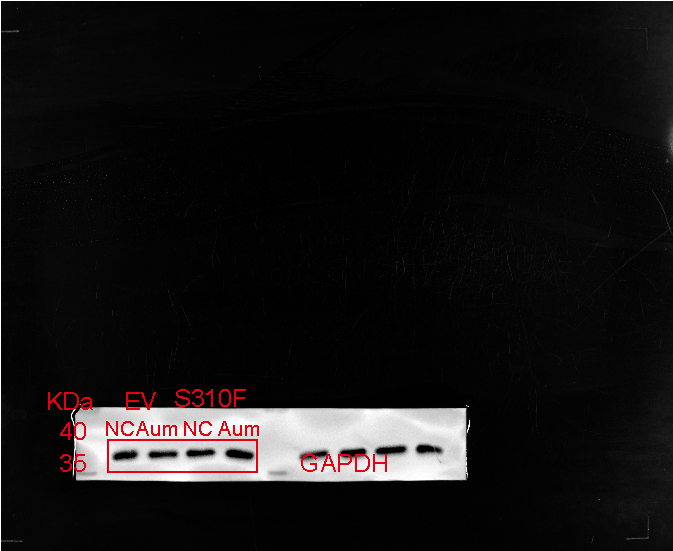

Supplement: Supplementary file 8 — Source data Fig. 5 [file 44321_2025_293_MOESM8_ESM.zip › Figure 5/5D/western GAPDH.tif]

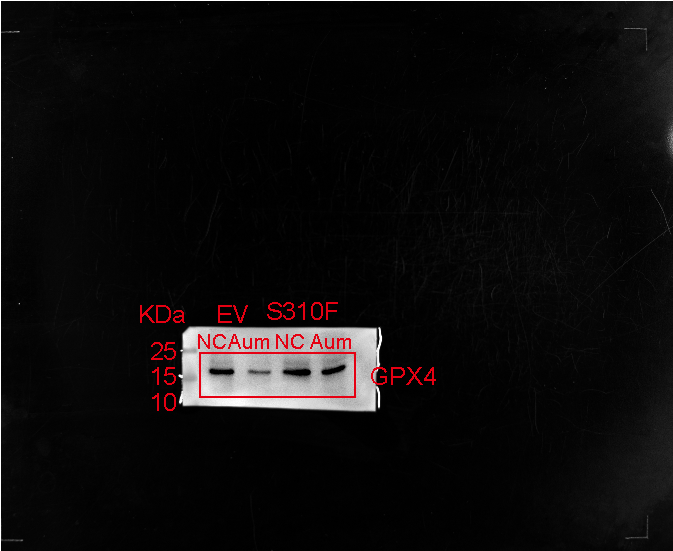

Supplement: Supplementary file 8 — Source data Fig. 5 [file 44321_2025_293_MOESM8_ESM.zip › Figure 5/5D/western GPX4.tif]

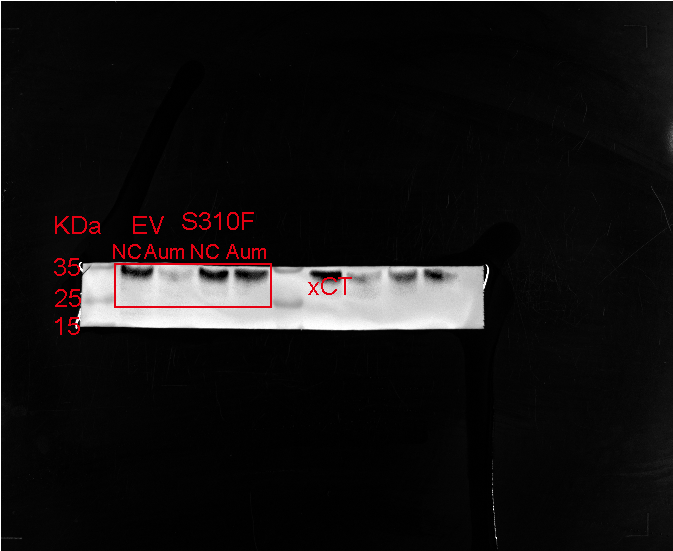

Supplement: Supplementary file 8 — Source data Fig. 5 [file 44321_2025_293_MOESM8_ESM.zip › Figure 5/5D/western xCT.tif]

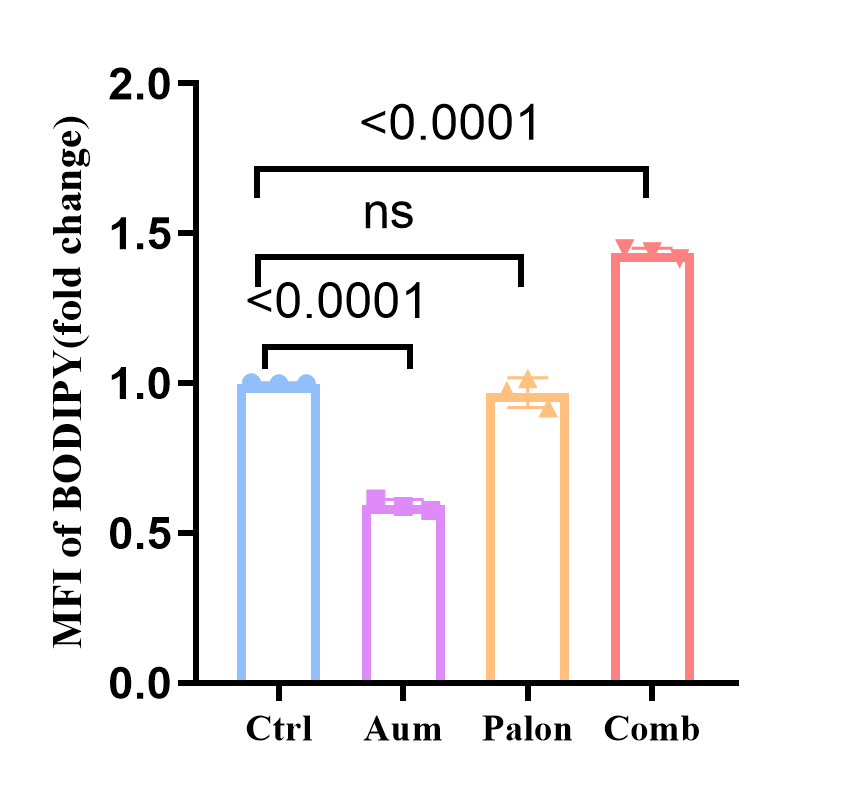

Supplement: Supplementary file 8 — Source data Fig. 5 [file 44321_2025_293_MOESM8_ESM.zip › Figure 5/5E/Fig 5E H1975.tif]

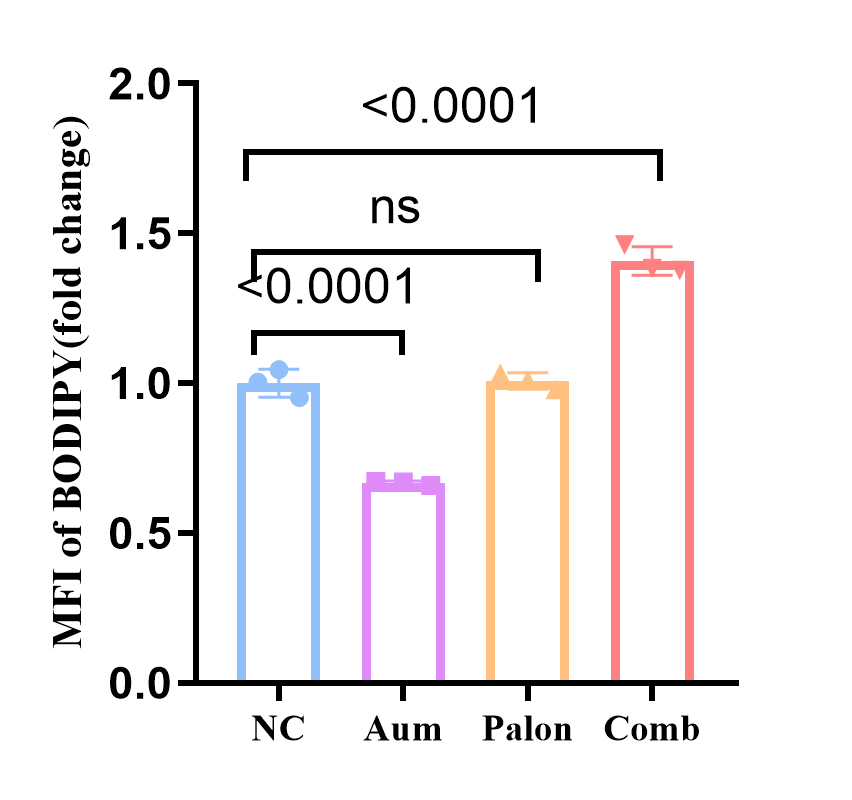

Supplement: Supplementary file 8 — Source data Fig. 5 [file 44321_2025_293_MOESM8_ESM.zip › Figure 5/5E/Fig 5E PC9.tif]

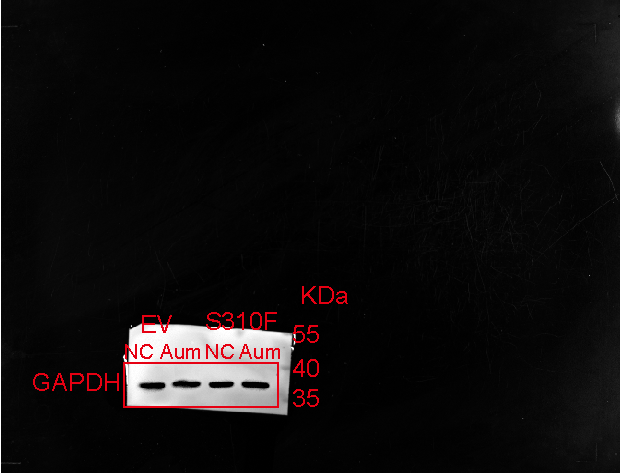

Supplement: Supplementary file 8 — Source data Fig. 5 [file 44321_2025_293_MOESM8_ESM.zip › Figure 5/5F/western H1975-GAPDH.tif]

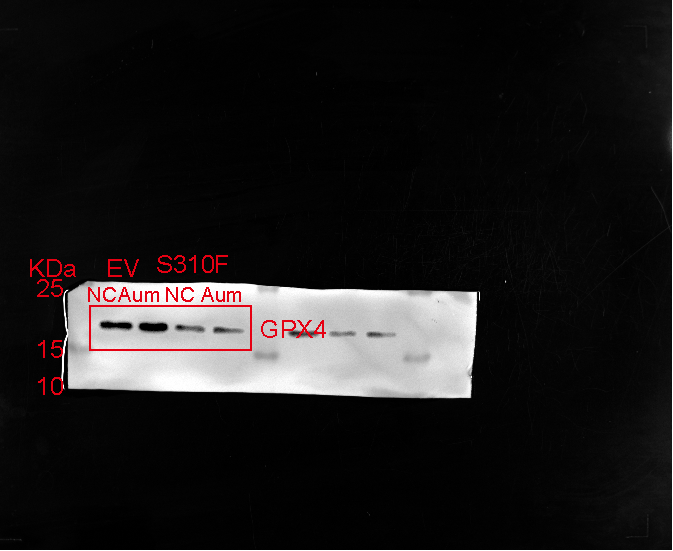

Supplement: Supplementary file 8 — Source data Fig. 5 [file 44321_2025_293_MOESM8_ESM.zip › Figure 5/5F/western H1975-GPX4.tif]

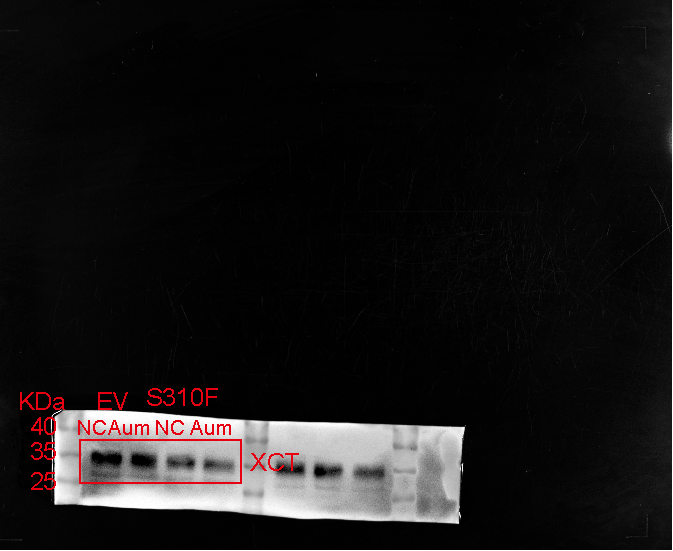

Supplement: Supplementary file 8 — Source data Fig. 5 [file 44321_2025_293_MOESM8_ESM.zip › Figure 5/5F/western H1975-xCT.tif]

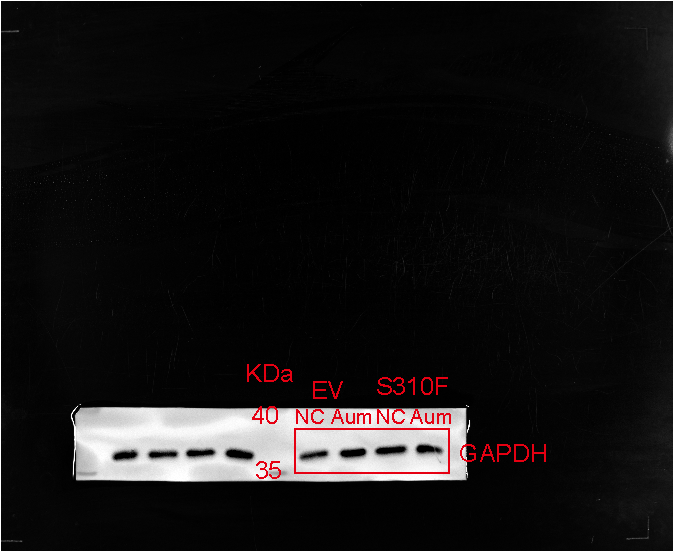

Supplement: Supplementary file 8 — Source data Fig. 5 [file 44321_2025_293_MOESM8_ESM.zip › Figure 5/5F/western PC9-GAPDH.tif]

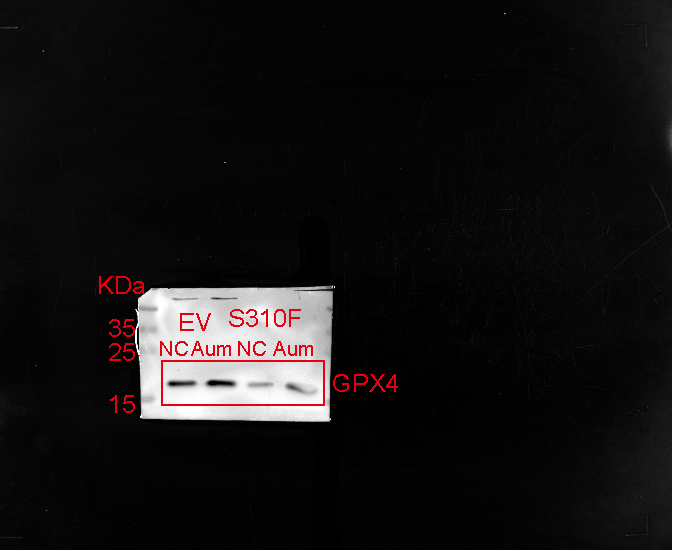

Supplement: Supplementary file 8 — Source data Fig. 5 [file 44321_2025_293_MOESM8_ESM.zip › Figure 5/5F/western PC9-GPX4.tif]

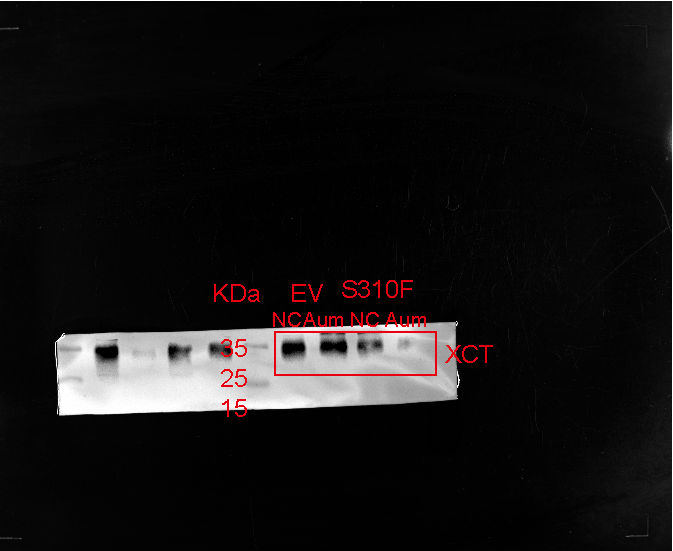

Supplement: Supplementary file 8 — Source data Fig. 5 [file 44321_2025_293_MOESM8_ESM.zip › Figure 5/5F/western PC9-xCT.tif]

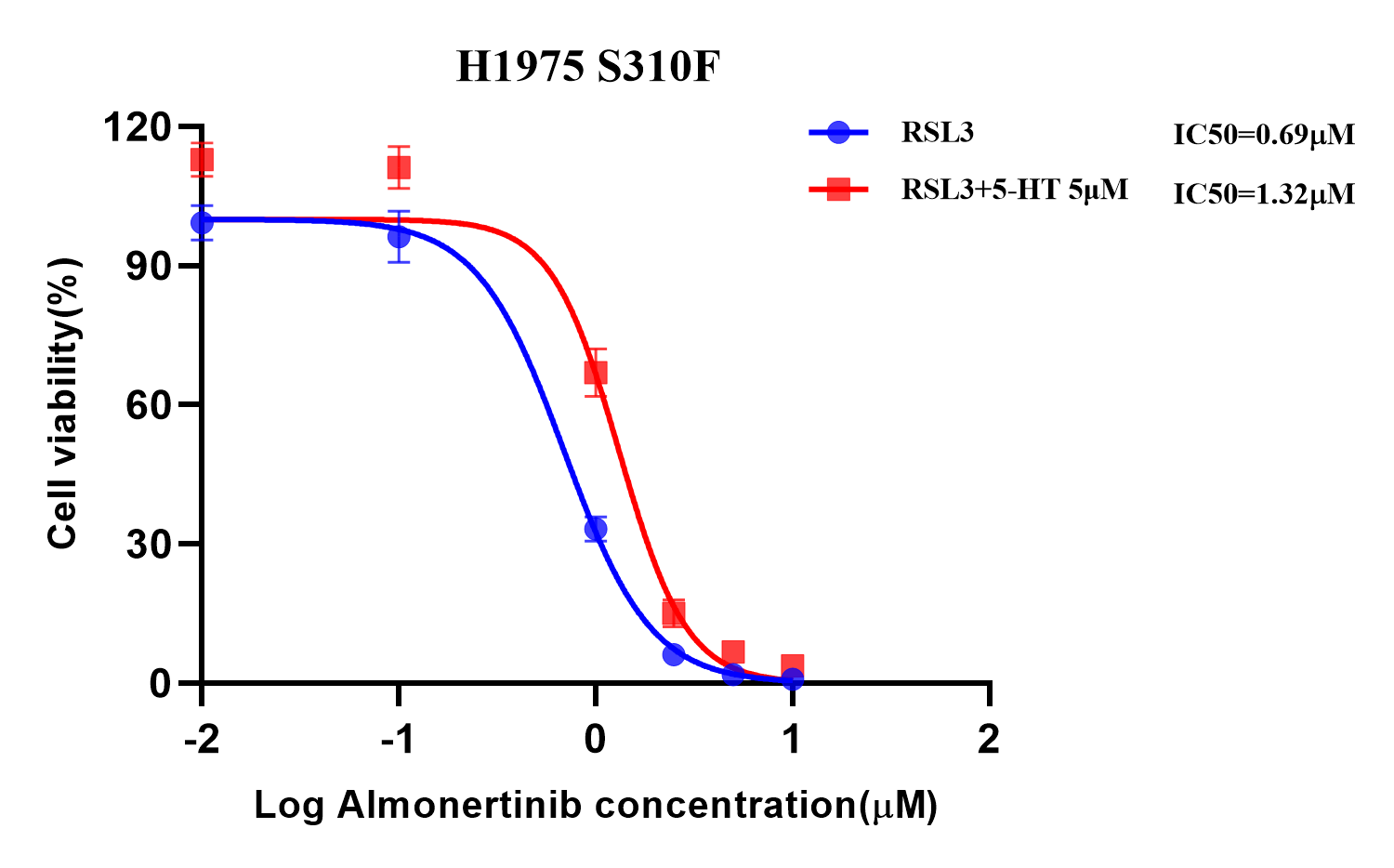

Supplement: Supplementary file 8 — Source data Fig. 5 [file 44321_2025_293_MOESM8_ESM.zip › Figure 5/5G/H1975 RSL3.tif]

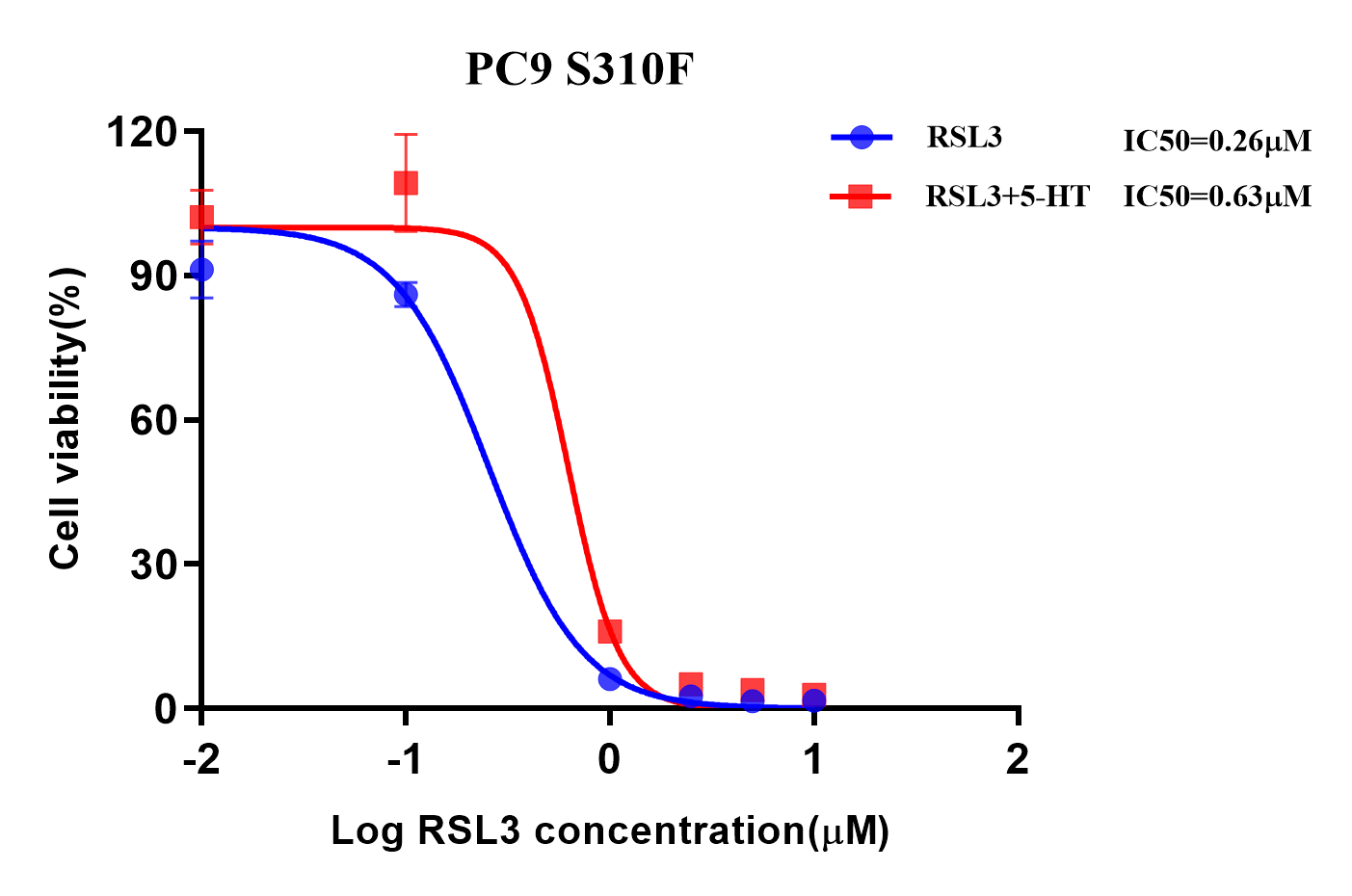

Supplement: Supplementary file 8 — Source data Fig. 5 [file 44321_2025_293_MOESM8_ESM.zip › Figure 5/5G/PC9 RSL3.tif]

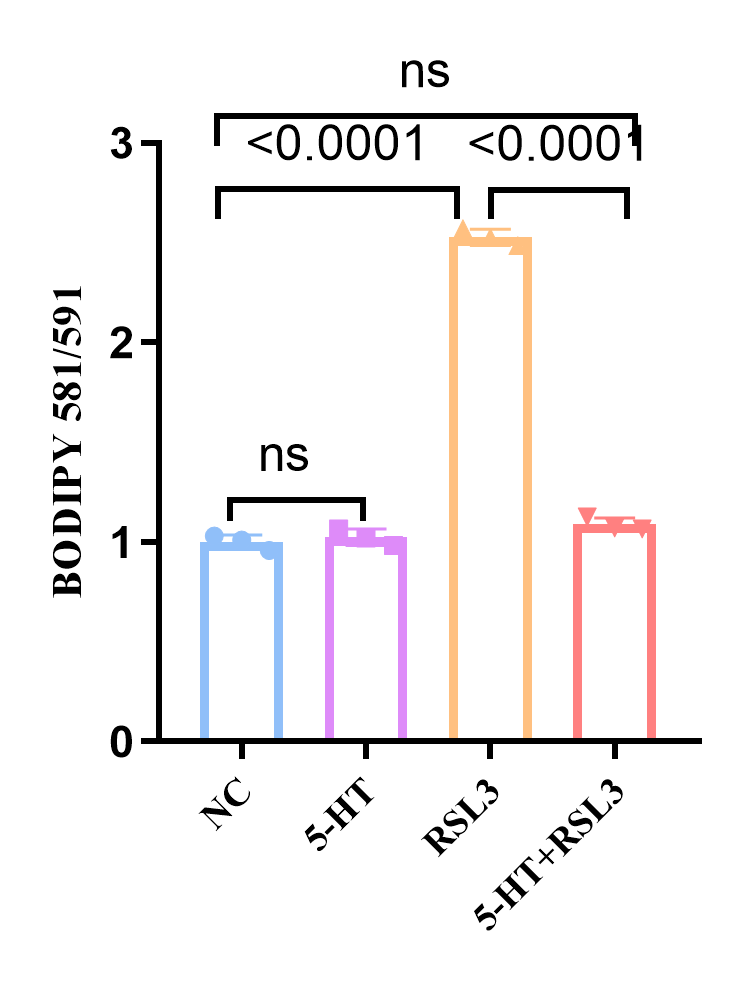

Supplement: Supplementary file 8 — Source data Fig. 5 [file 44321_2025_293_MOESM8_ESM.zip › Figure 5/5H/Fig 5H H1975.tif]

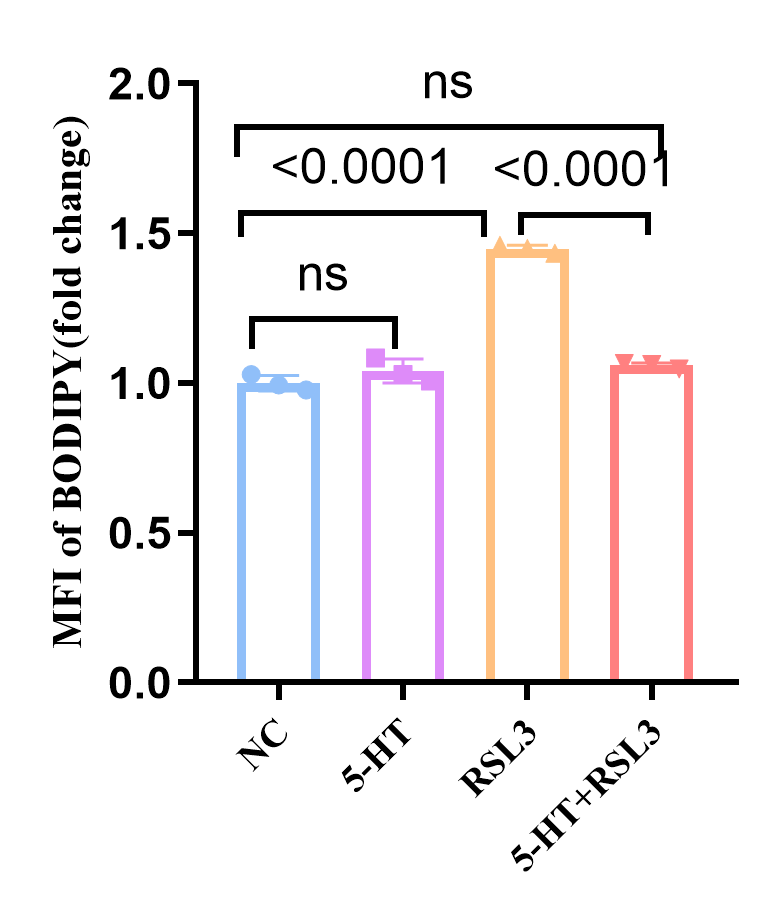

Supplement: Supplementary file 8 — Source data Fig. 5 [file 44321_2025_293_MOESM8_ESM.zip › Figure 5/5H/Fig 5H PC9.tif]

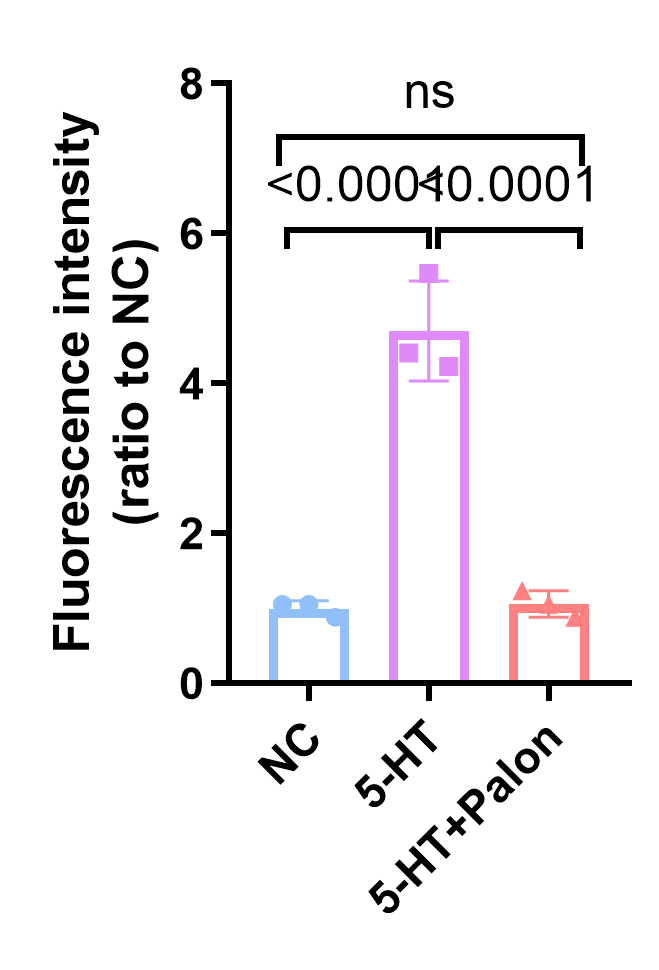

Supplement: Supplementary file 8 — Source data Fig. 5 [file 44321_2025_293_MOESM8_ESM.zip › Figure 5/5I/Fig 5I H1975.tif]

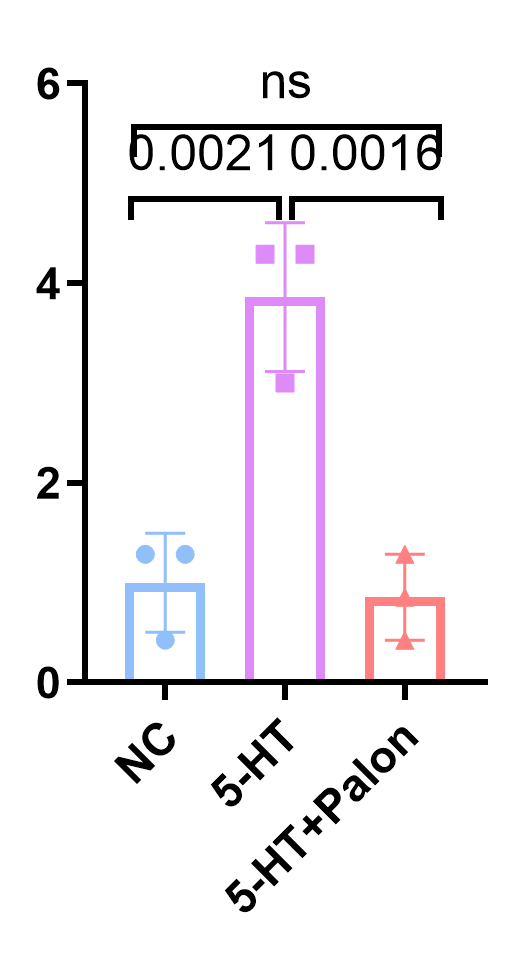

Supplement: Supplementary file 8 — Source data Fig. 5 [file 44321_2025_293_MOESM8_ESM.zip › Figure 5/5I/Fig 5I PC9.tif]

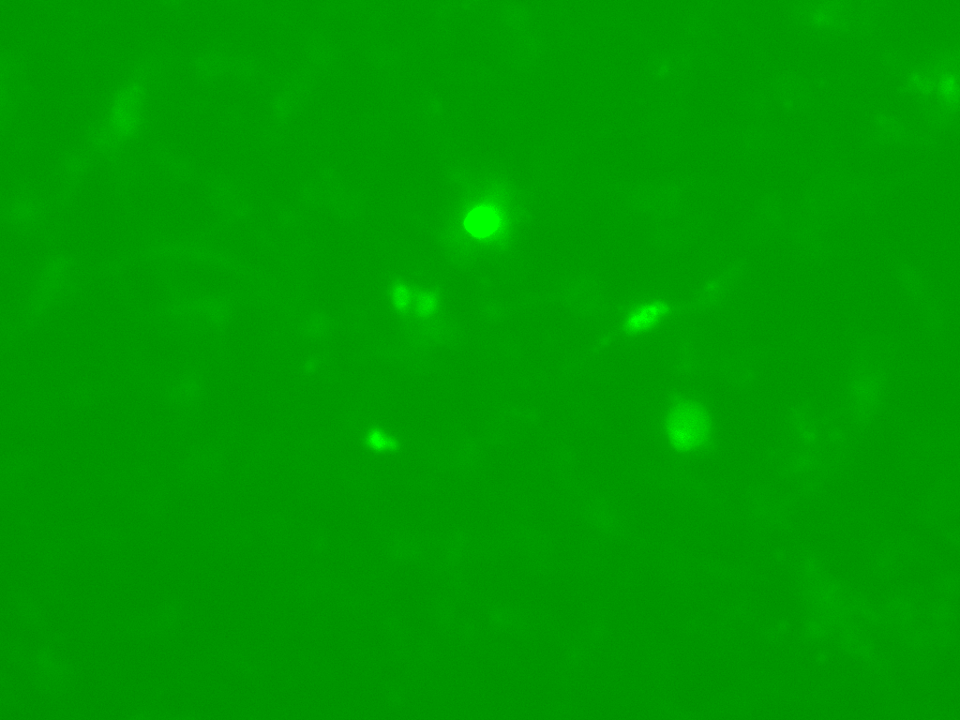

Supplement: Supplementary file 8 — Source data Fig. 5 [file 44321_2025_293_MOESM8_ESM.zip › Figure 5/5I/H1975 picture/NC/NC_0002_GFP .tif]

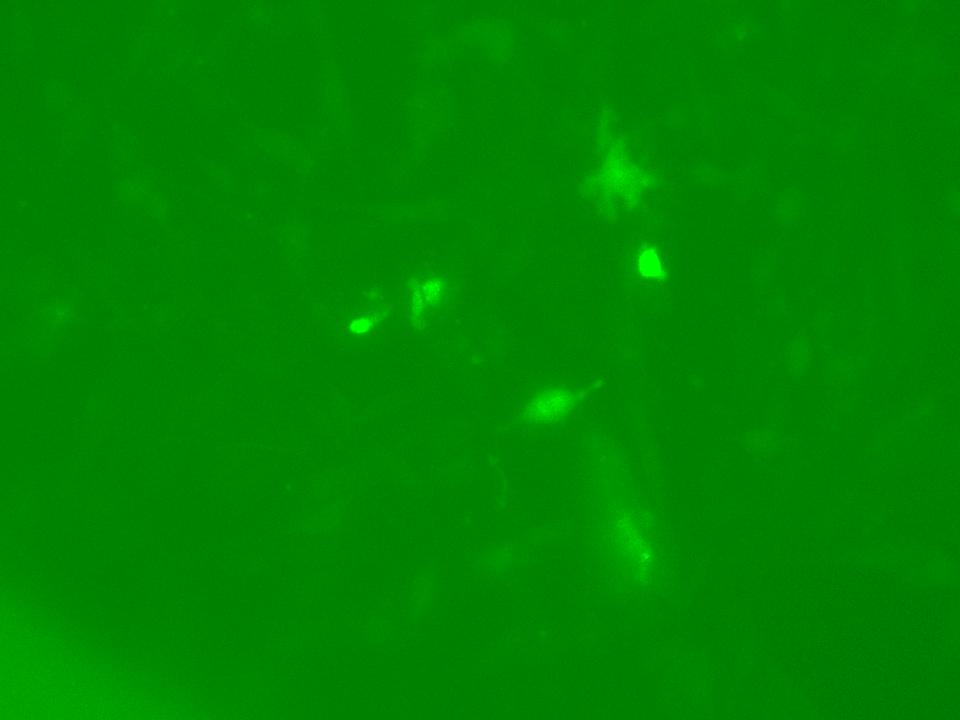

Supplement: Supplementary file 8 — Source data Fig. 5 [file 44321_2025_293_MOESM8_ESM.zip › Figure 5/5I/H1975 picture/NC/NC_0003_GFP.tif]

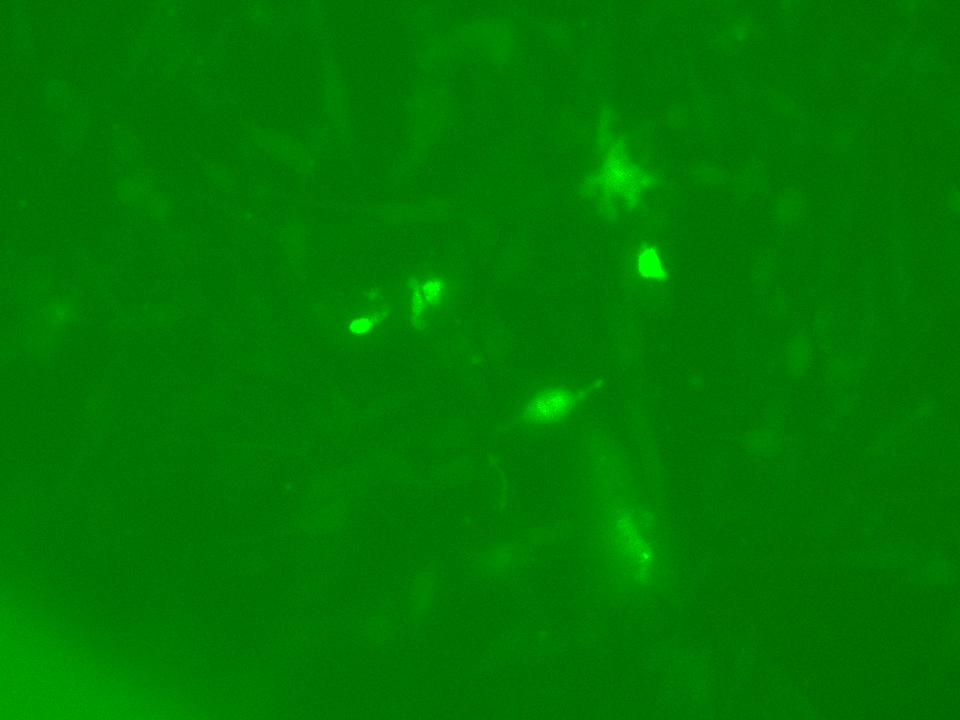

Supplement: Supplementary file 8 — Source data Fig. 5 [file 44321_2025_293_MOESM8_ESM.zip › Figure 5/5I/H1975 picture/Palon/PAL_0002_GFP.tif]

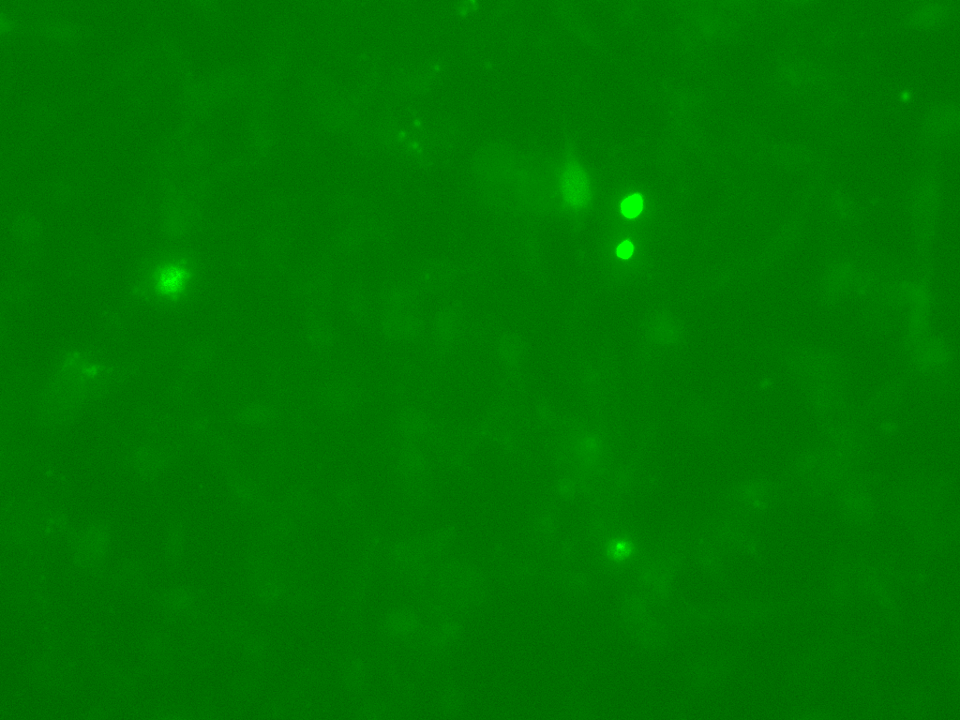

Supplement: Supplementary file 8 — Source data Fig. 5 [file 44321_2025_293_MOESM8_ESM.zip › Figure 5/5I/H1975 picture/Palon/PAL_0003_GFP.tif]

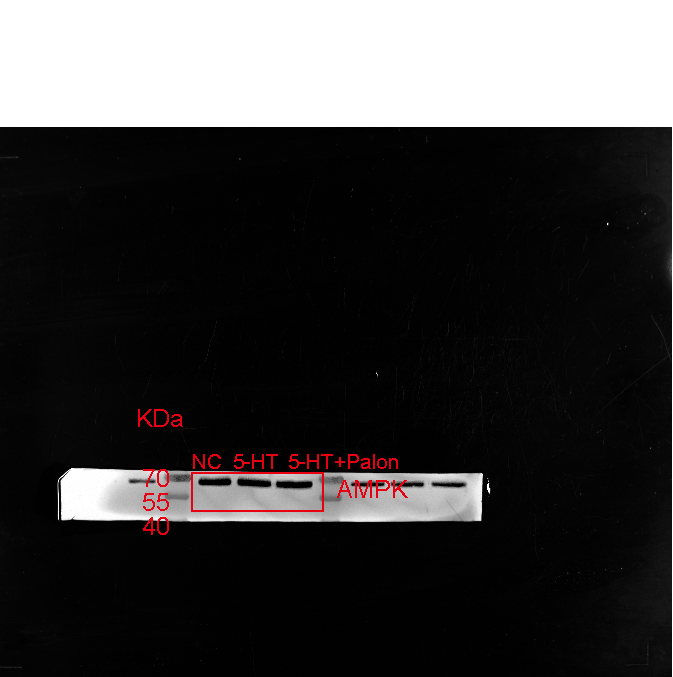

Supplement: Supplementary file 8 — Source data Fig. 5 [file 44321_2025_293_MOESM8_ESM.zip › Figure 5/5J/western H1975_AMPK.tif]

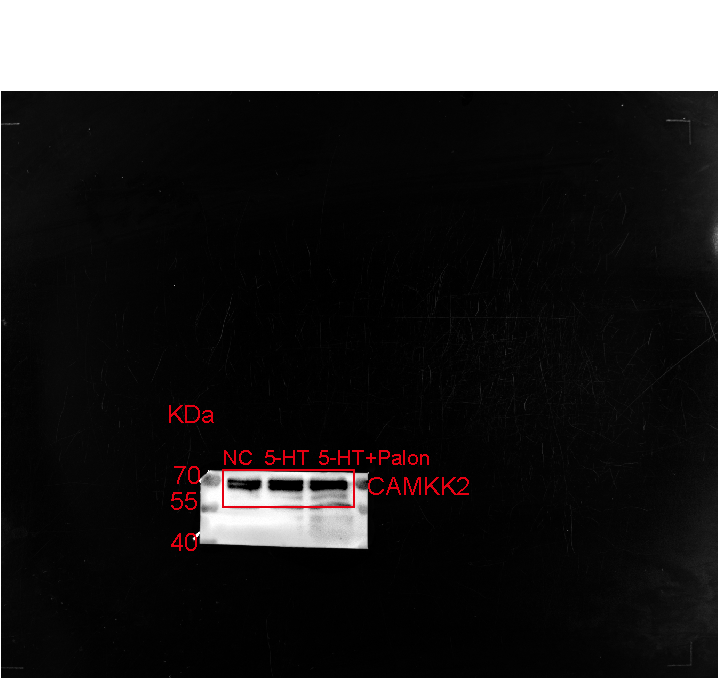

Supplement: Supplementary file 8 — Source data Fig. 5 [file 44321_2025_293_MOESM8_ESM.zip › Figure 5/5J/western H1975_CAMKK2.tif]

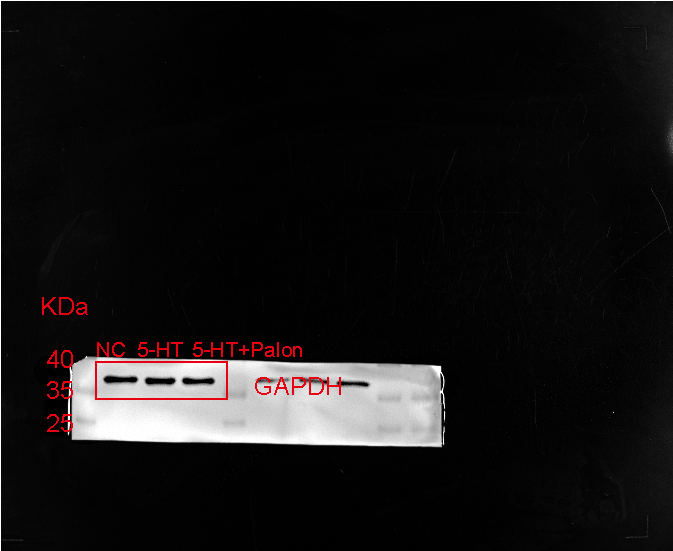

Supplement: Supplementary file 8 — Source data Fig. 5 [file 44321_2025_293_MOESM8_ESM.zip › Figure 5/5J/western H1975_GAPDH-1.tif]

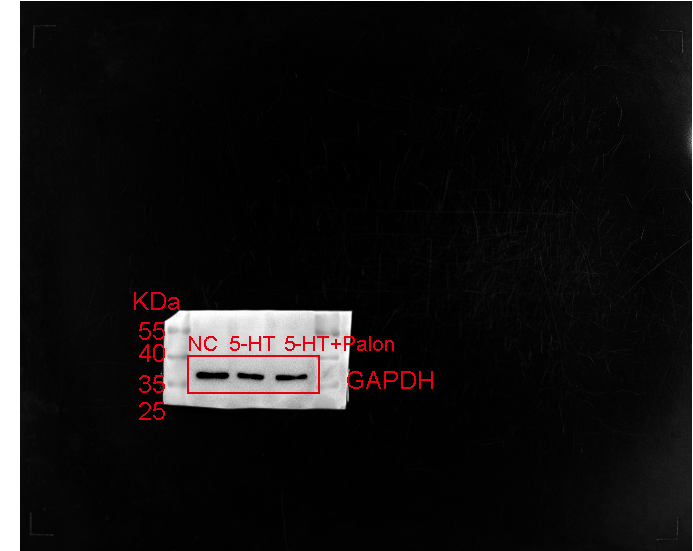

Supplement: Supplementary file 8 — Source data Fig. 5 [file 44321_2025_293_MOESM8_ESM.zip › Figure 5/5J/western H1975_GAPDH-2.tif]

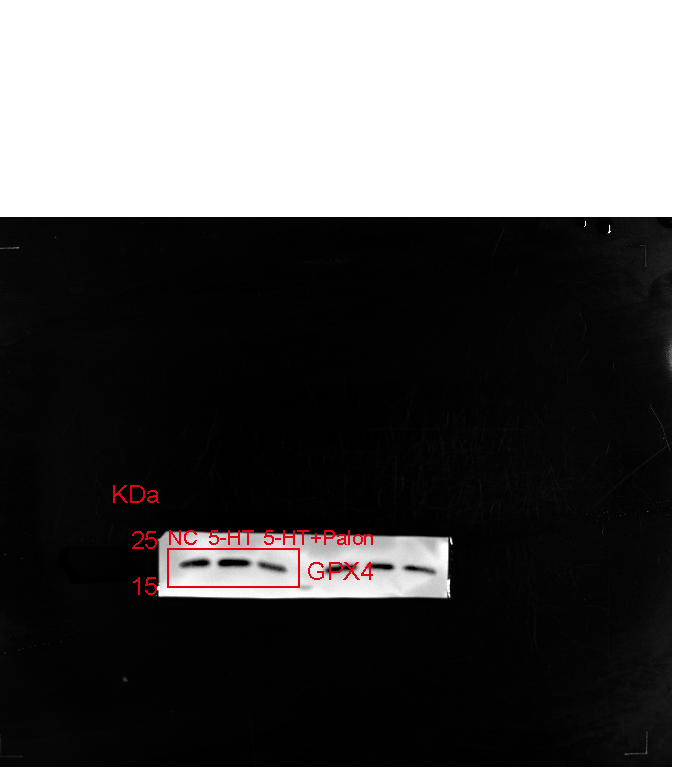

Supplement: Supplementary file 8 — Source data Fig. 5 [file 44321_2025_293_MOESM8_ESM.zip › Figure 5/5J/western H1975_GPX4.tif]

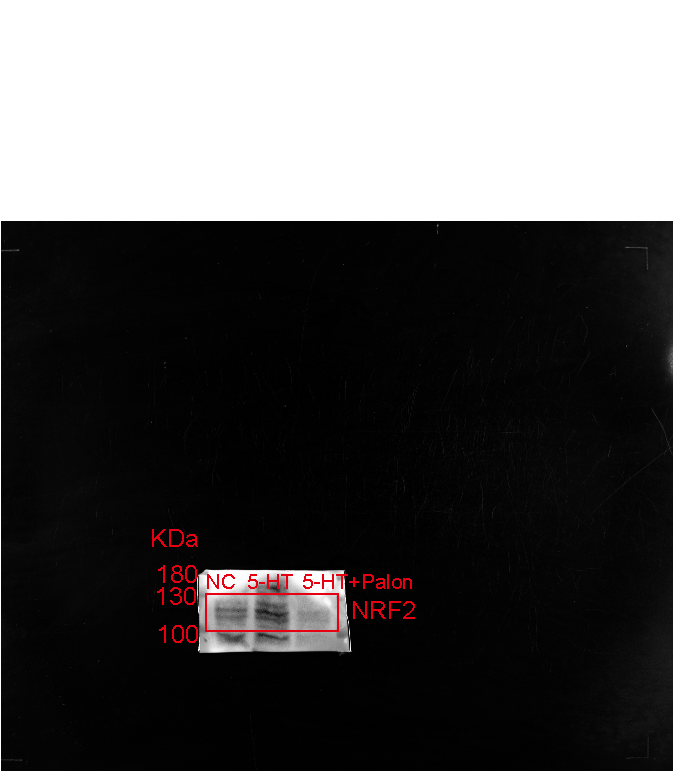

Supplement: Supplementary file 8 — Source data Fig. 5 [file 44321_2025_293_MOESM8_ESM.zip › Figure 5/5J/western H1975_NRF2.tif]

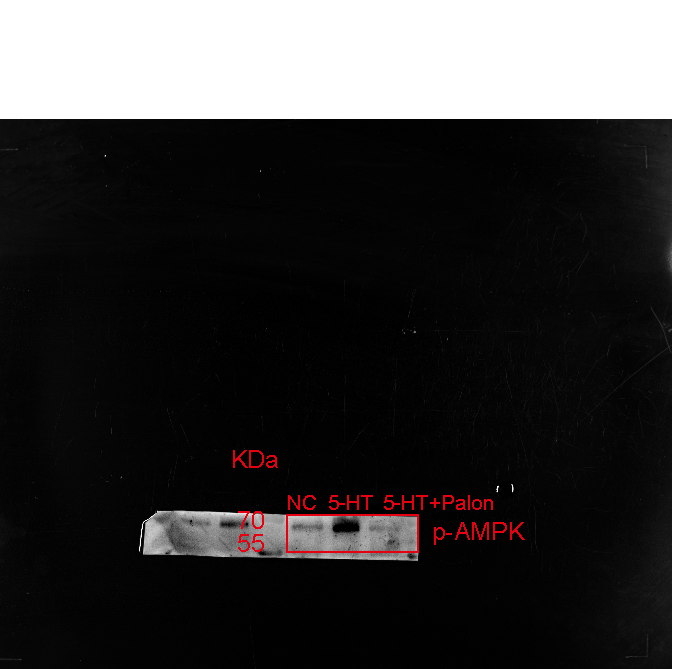

Supplement: Supplementary file 8 — Source data Fig. 5 [file 44321_2025_293_MOESM8_ESM.zip › Figure 5/5J/western H1975_p-AMPK.tif]

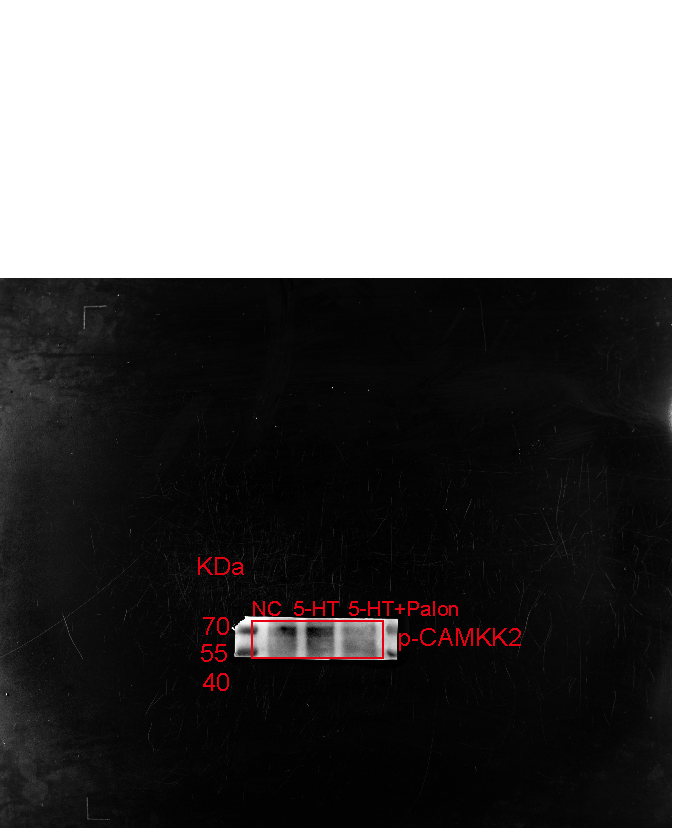

Supplement: Supplementary file 8 — Source data Fig. 5 [file 44321_2025_293_MOESM8_ESM.zip › Figure 5/5J/western H1975_p-CAMKK2.tif]

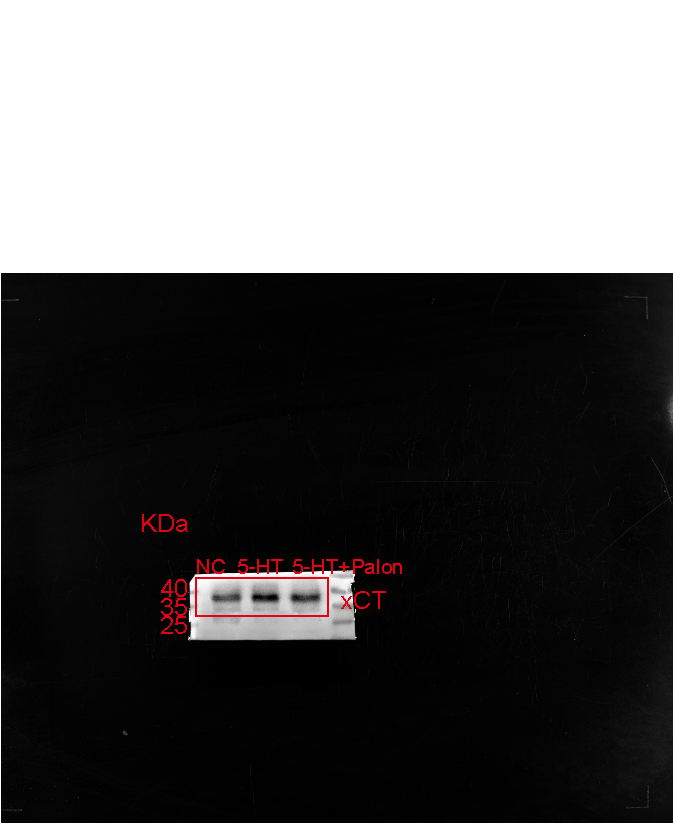

Supplement: Supplementary file 8 — Source data Fig. 5 [file 44321_2025_293_MOESM8_ESM.zip › Figure 5/5J/western H1975_xCT.tif]

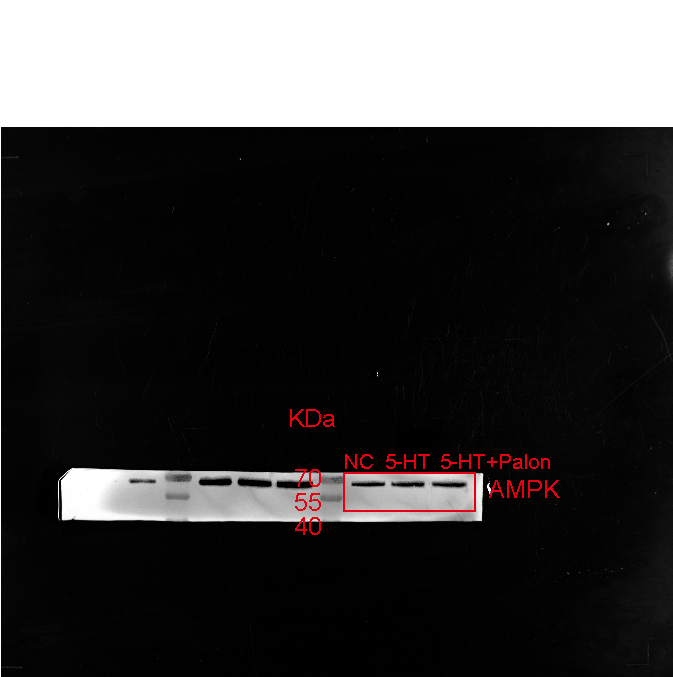

Supplement: Supplementary file 8 — Source data Fig. 5 [file 44321_2025_293_MOESM8_ESM.zip › Figure 5/5J/western PC9_AMPK.tif]

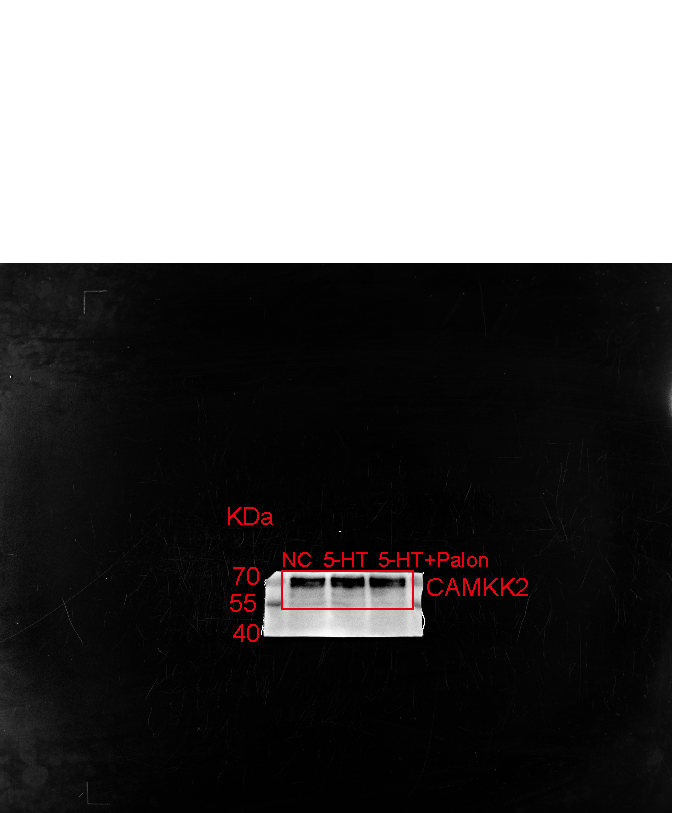

Supplement: Supplementary file 8 — Source data Fig. 5 [file 44321_2025_293_MOESM8_ESM.zip › Figure 5/5J/western PC9_CAMKK2.tif]

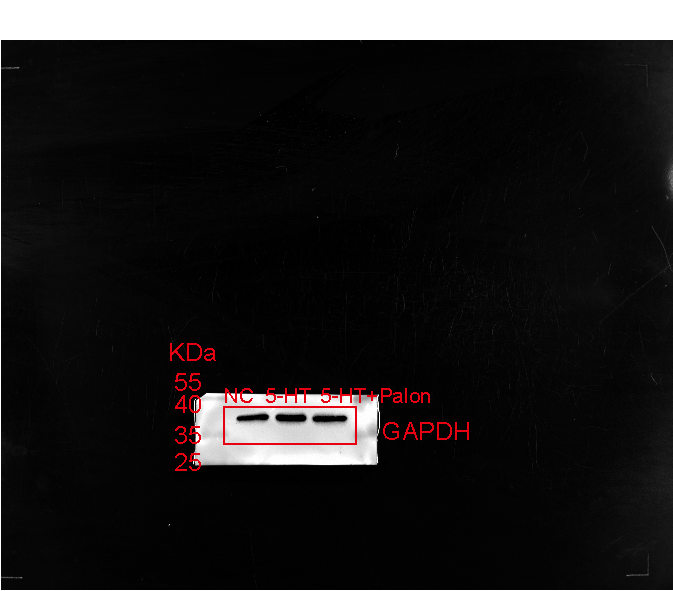

Supplement: Supplementary file 8 — Source data Fig. 5 [file 44321_2025_293_MOESM8_ESM.zip › Figure 5/5J/western PC9_GAPDH-1.tif]

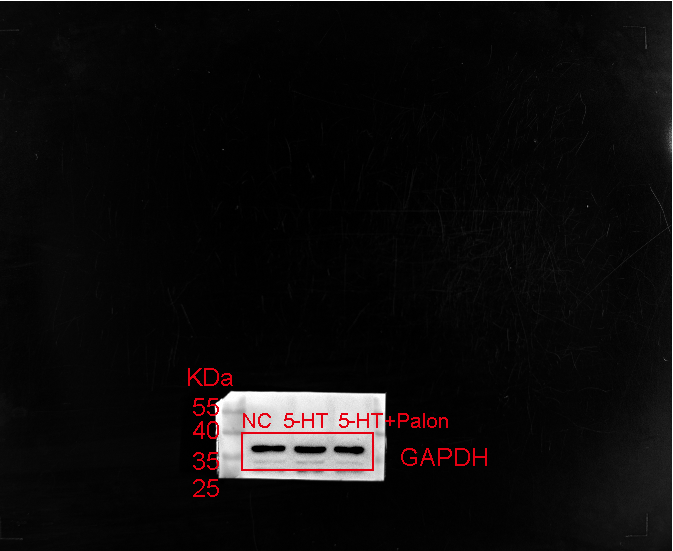

Supplement: Supplementary file 8 — Source data Fig. 5 [file 44321_2025_293_MOESM8_ESM.zip › Figure 5/5J/western PC9_GAPDH-2.tif]

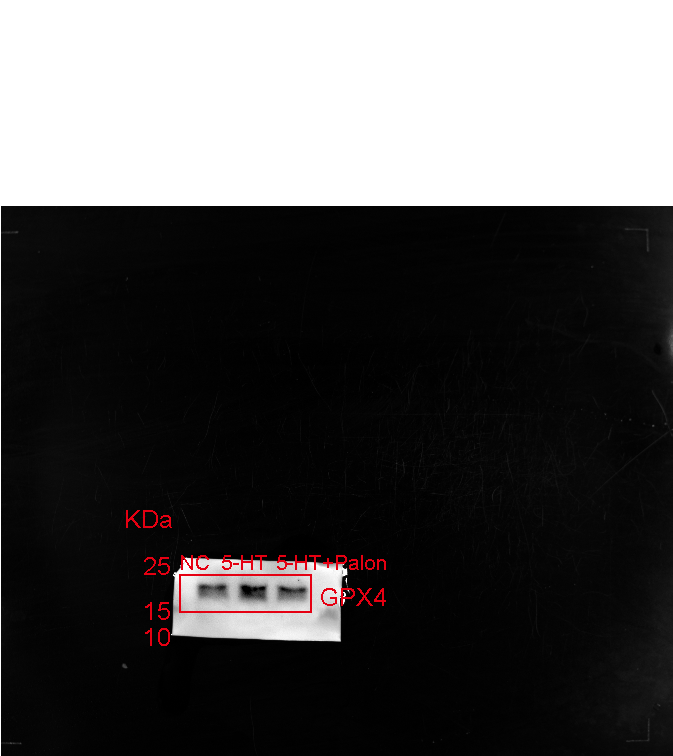

Supplement: Supplementary file 8 — Source data Fig. 5 [file 44321_2025_293_MOESM8_ESM.zip › Figure 5/5J/western PC9_GPX4.tif]

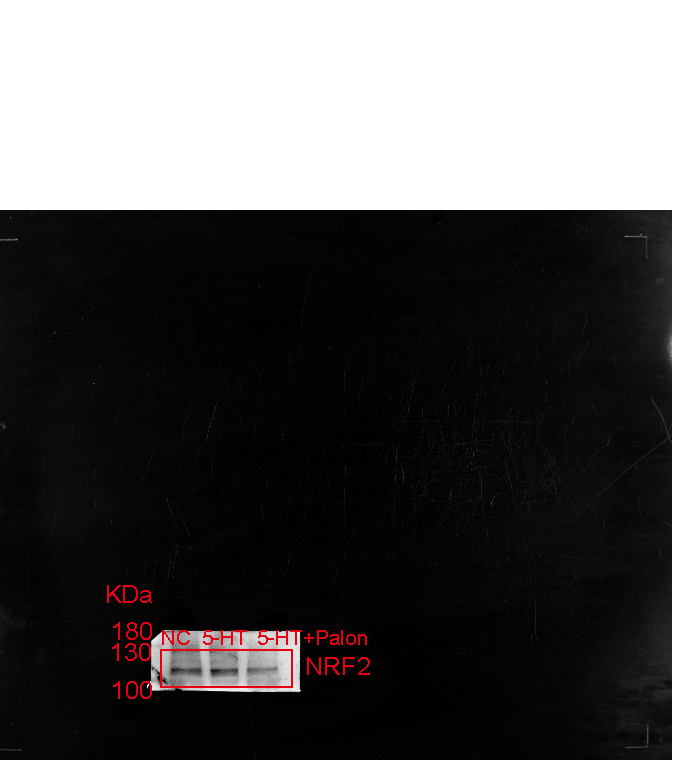

Supplement: Supplementary file 8 — Source data Fig. 5 [file 44321_2025_293_MOESM8_ESM.zip › Figure 5/5J/western PC9_NRF2.tif]

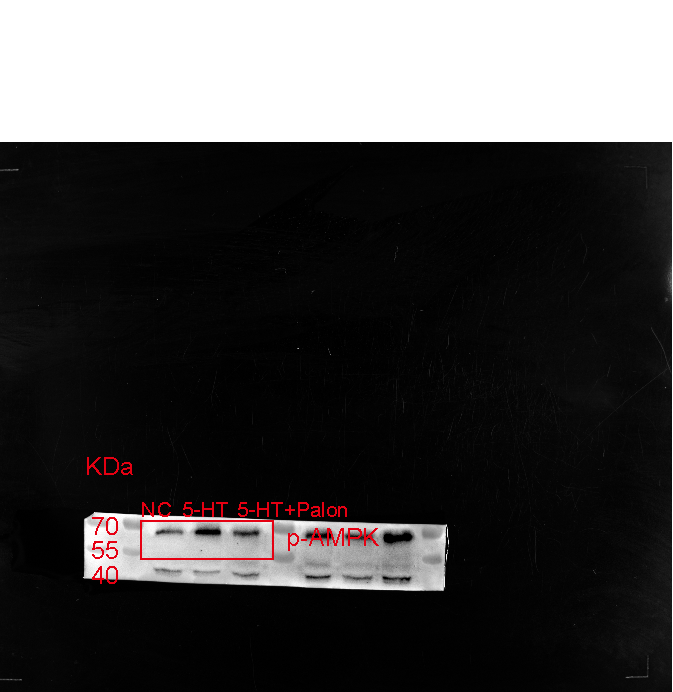

Supplement: Supplementary file 8 — Source data Fig. 5 [file 44321_2025_293_MOESM8_ESM.zip › Figure 5/5J/western PC9_p-AMPK.tif]

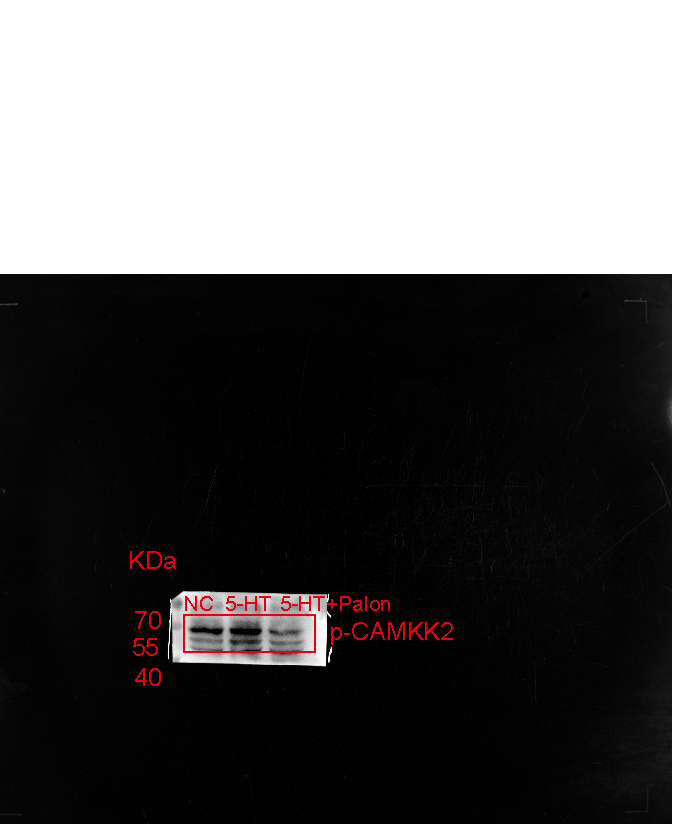

Supplement: Supplementary file 8 — Source data Fig. 5 [file 44321_2025_293_MOESM8_ESM.zip › Figure 5/5J/western PC9_p-CAMKK2.tif]

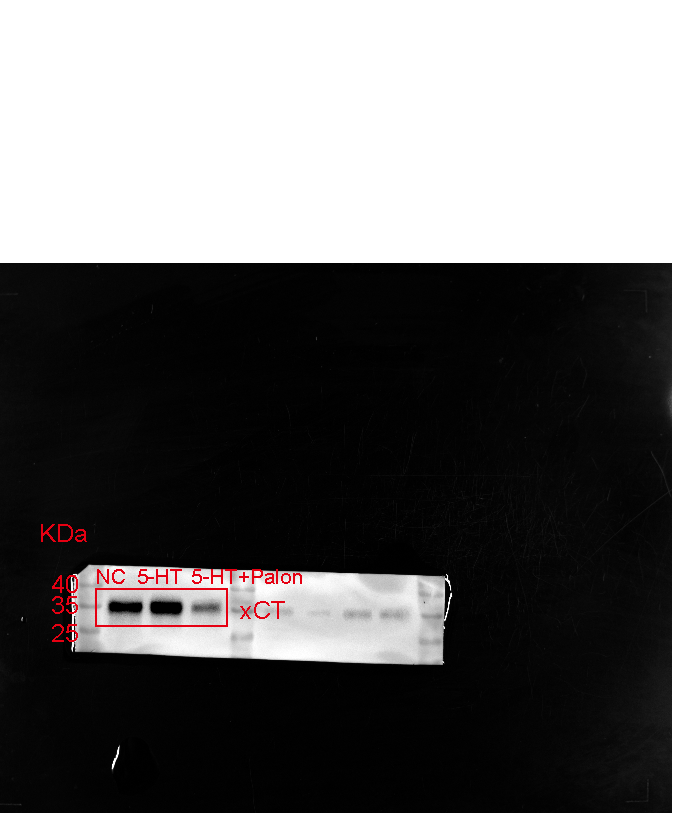

Supplement: Supplementary file 8 — Source data Fig. 5 [file 44321_2025_293_MOESM8_ESM.zip › Figure 5/5J/western PC9_xCT.tif]

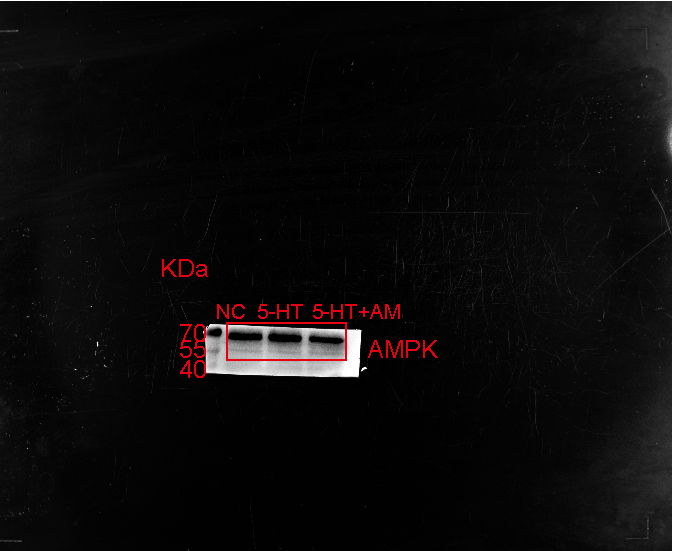

Supplement: Supplementary file 8 — Source data Fig. 5 [file 44321_2025_293_MOESM8_ESM.zip › Figure 5/5K/western H1975_AMPK.tif]

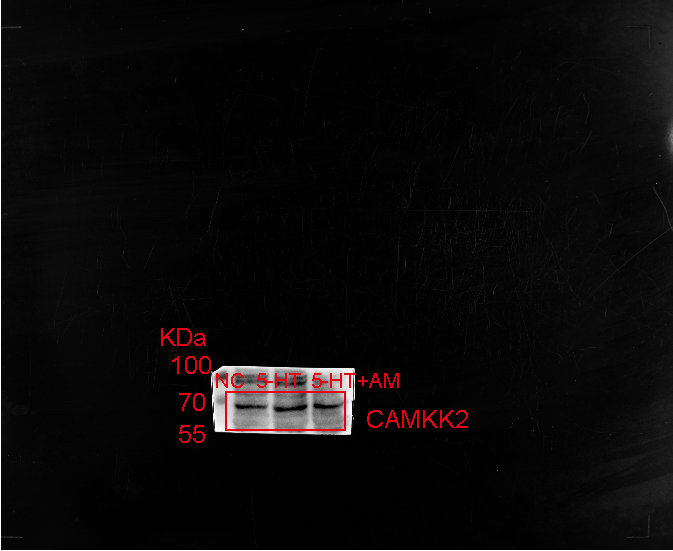

Supplement: Supplementary file 8 — Source data Fig. 5 [file 44321_2025_293_MOESM8_ESM.zip › Figure 5/5K/western H1975_CAMKK2.tif]

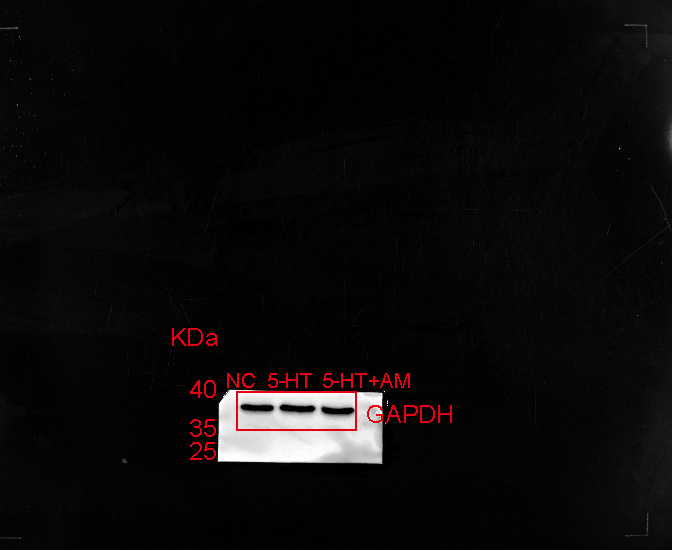

Supplement: Supplementary file 8 — Source data Fig. 5 [file 44321_2025_293_MOESM8_ESM.zip › Figure 5/5K/western H1975_GAPDH-1.tif]

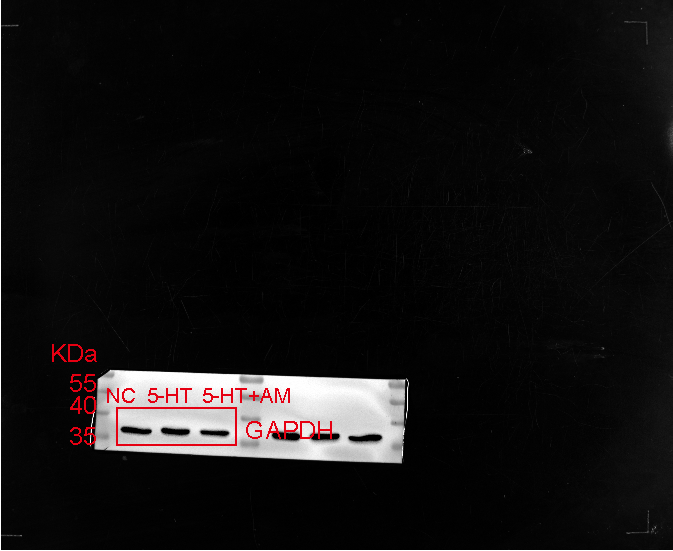

Supplement: Supplementary file 8 — Source data Fig. 5 [file 44321_2025_293_MOESM8_ESM.zip › Figure 5/5K/western H1975_GAPDH-2.tif]

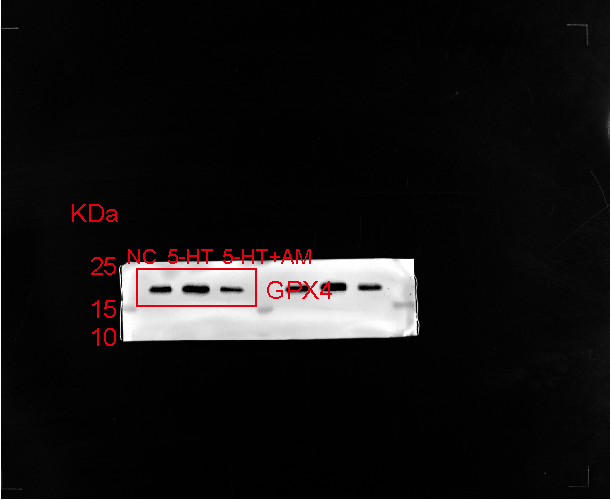

Supplement: Supplementary file 8 — Source data Fig. 5 [file 44321_2025_293_MOESM8_ESM.zip › Figure 5/5K/western H1975_GPX4.tif]

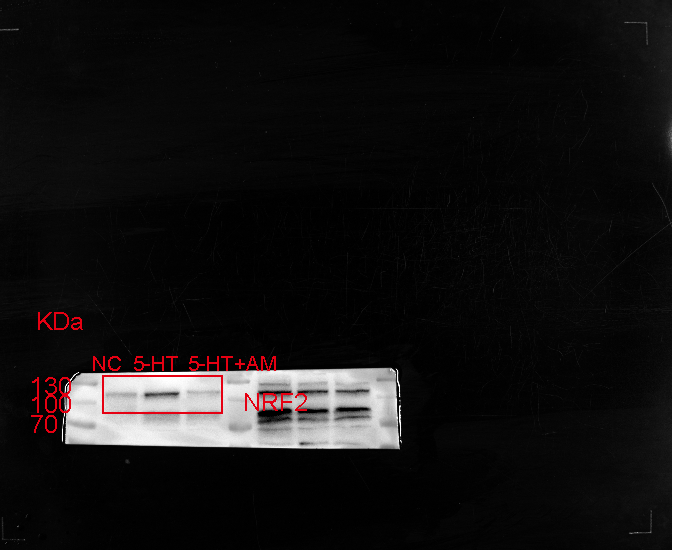

Supplement: Supplementary file 8 — Source data Fig. 5 [file 44321_2025_293_MOESM8_ESM.zip › Figure 5/5K/western H1975_NRF2.tif]

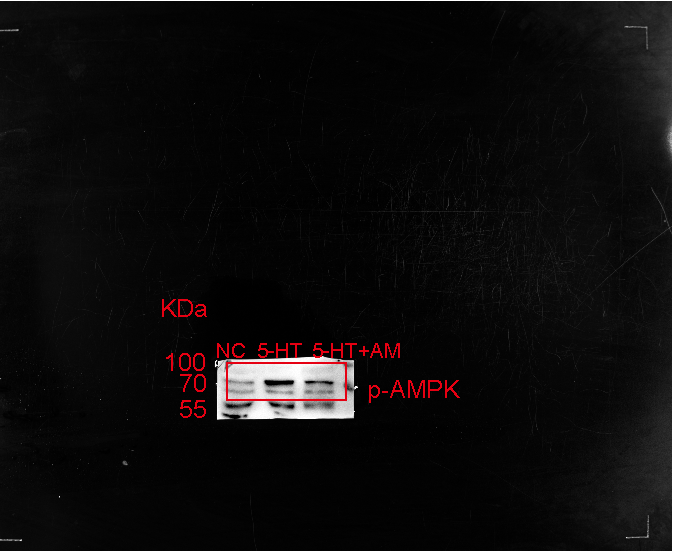

Supplement: Supplementary file 8 — Source data Fig. 5 [file 44321_2025_293_MOESM8_ESM.zip › Figure 5/5K/western H1975_p-AMPK.tif]

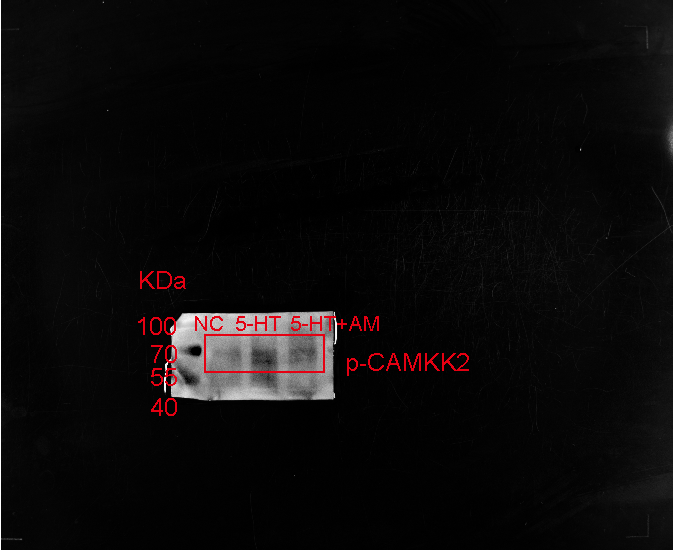

Supplement: Supplementary file 8 — Source data Fig. 5 [file 44321_2025_293_MOESM8_ESM.zip › Figure 5/5K/western H1975_p-CAMKK2.tif]

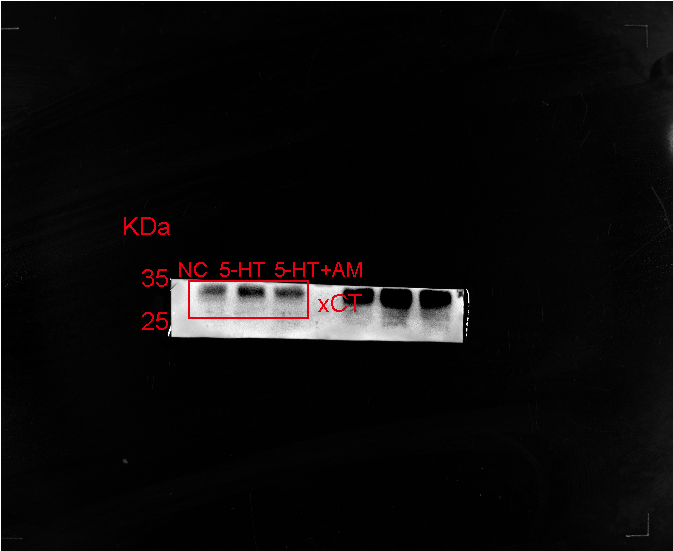

Supplement: Supplementary file 8 — Source data Fig. 5 [file 44321_2025_293_MOESM8_ESM.zip › Figure 5/5K/western H1975_xCT.tif]

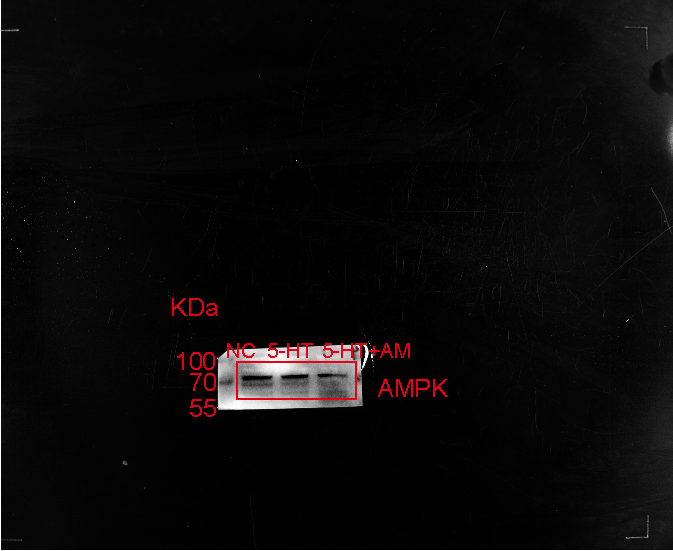

Supplement: Supplementary file 8 — Source data Fig. 5 [file 44321_2025_293_MOESM8_ESM.zip › Figure 5/5K/western PC9_AMPK.tif]

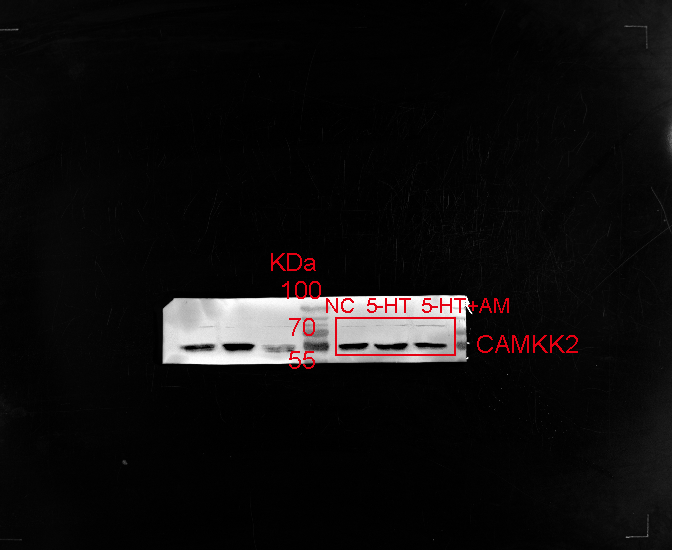

Supplement: Supplementary file 8 — Source data Fig. 5 [file 44321_2025_293_MOESM8_ESM.zip › Figure 5/5K/western PC9_CAMKK2.tif]

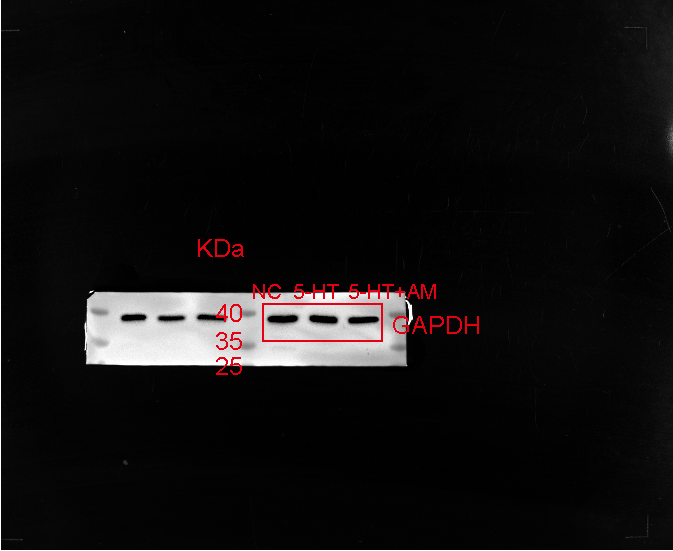

Supplement: Supplementary file 8 — Source data Fig. 5 [file 44321_2025_293_MOESM8_ESM.zip › Figure 5/5K/western PC9_GAPDH-1.tif]

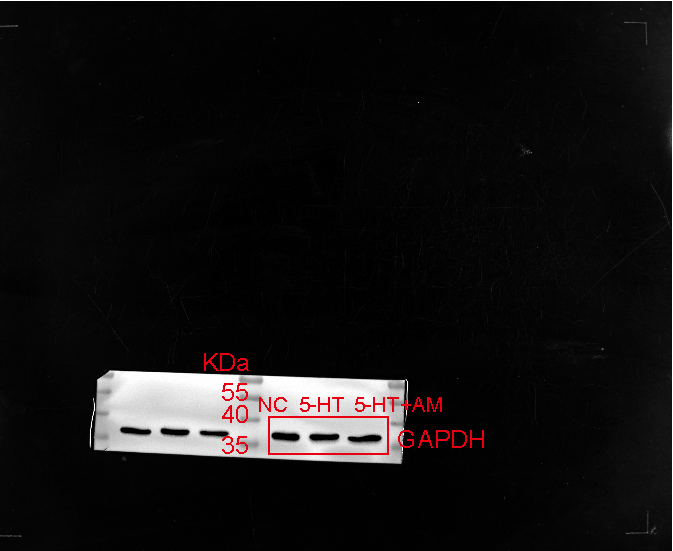

Supplement: Supplementary file 8 — Source data Fig. 5 [file 44321_2025_293_MOESM8_ESM.zip › Figure 5/5K/western PC9_GAPDH-2.tif]

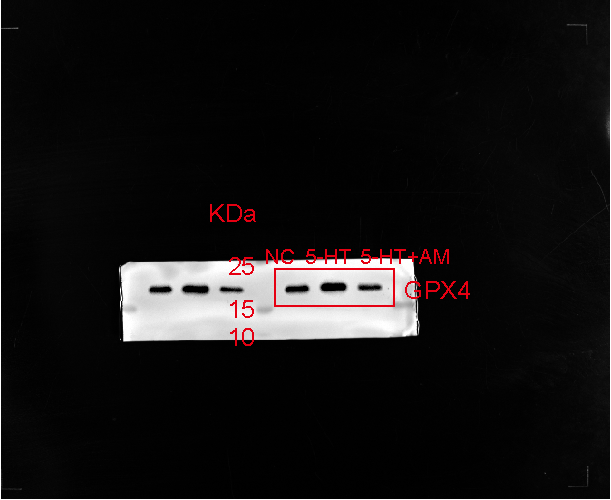

Supplement: Supplementary file 8 — Source data Fig. 5 [file 44321_2025_293_MOESM8_ESM.zip › Figure 5/5K/western PC9_GPX4.tif]

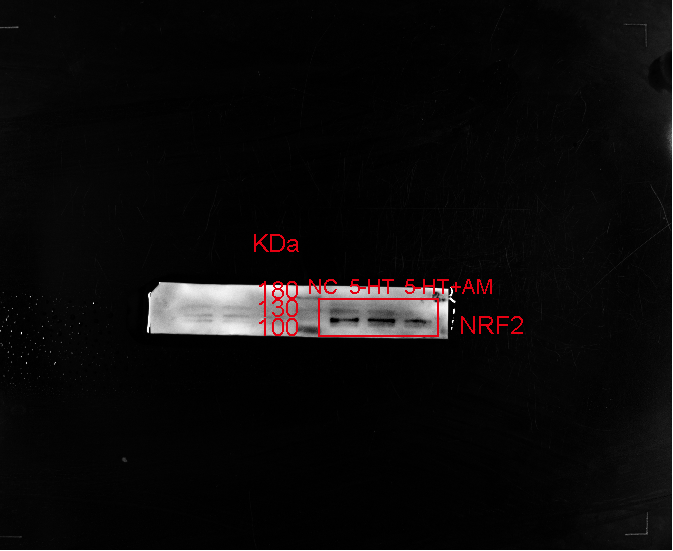

Supplement: Supplementary file 8 — Source data Fig. 5 [file 44321_2025_293_MOESM8_ESM.zip › Figure 5/5K/western PC9_NRF2.tif]

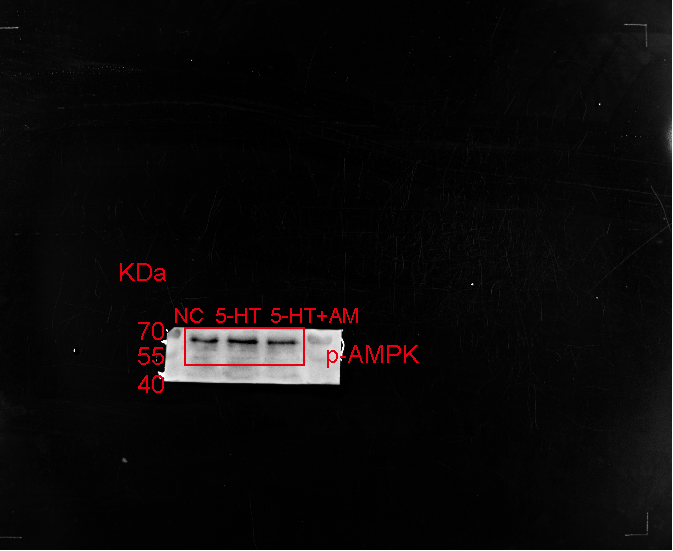

Supplement: Supplementary file 8 — Source data Fig. 5 [file 44321_2025_293_MOESM8_ESM.zip › Figure 5/5K/western PC9_p-AMPK.tif]

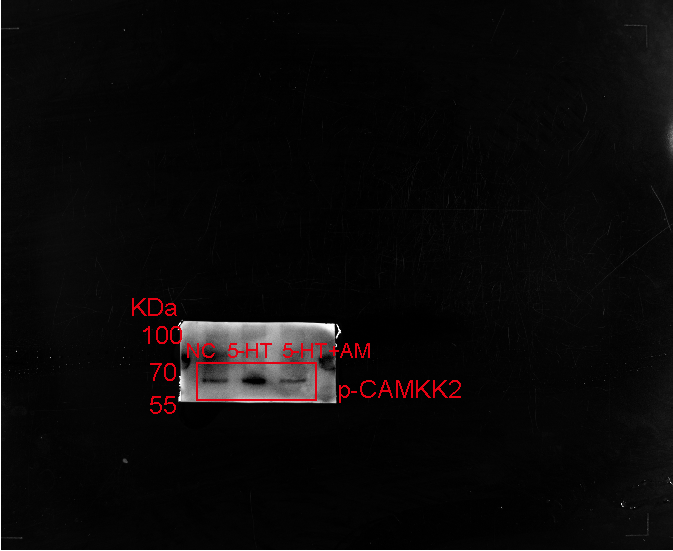

Supplement: Supplementary file 8 — Source data Fig. 5 [file 44321_2025_293_MOESM8_ESM.zip › Figure 5/5K/western PC9_p-CAMKK2.tif]

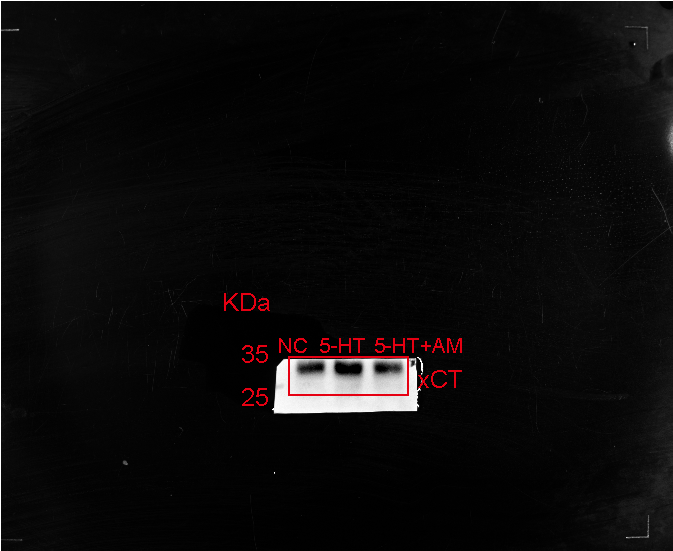

Supplement: Supplementary file 8 — Source data Fig. 5 [file 44321_2025_293_MOESM8_ESM.zip › Figure 5/5K/western PC9_xCT.tif]

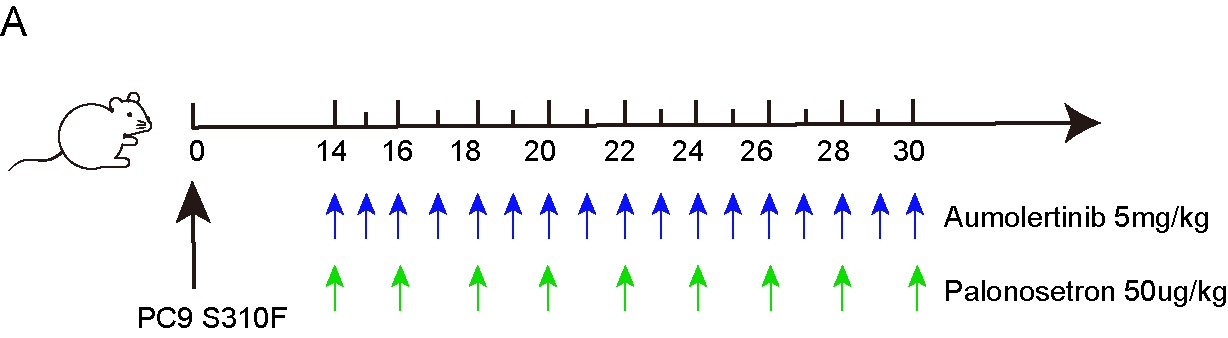

Supplement: Supplementary file 9 — Source data Fig. 6 [file 44321_2025_293_MOESM9_ESM.zip › Figure 6/6A/6A.tif]

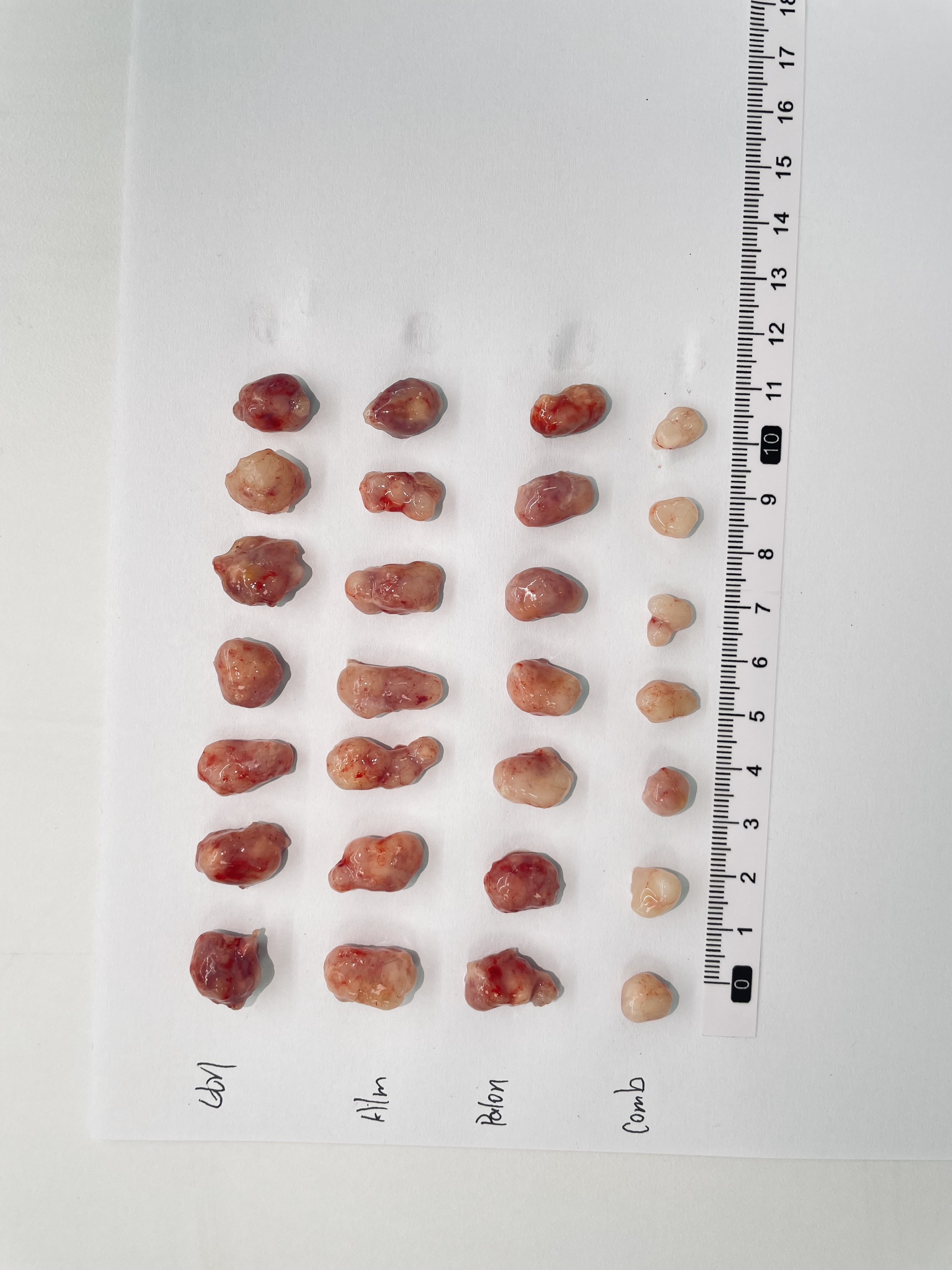

Supplement: Supplementary file 9 — Source data Fig. 6 [file 44321_2025_293_MOESM9_ESM.zip › Figure 6/6B/Tumor.jpg]

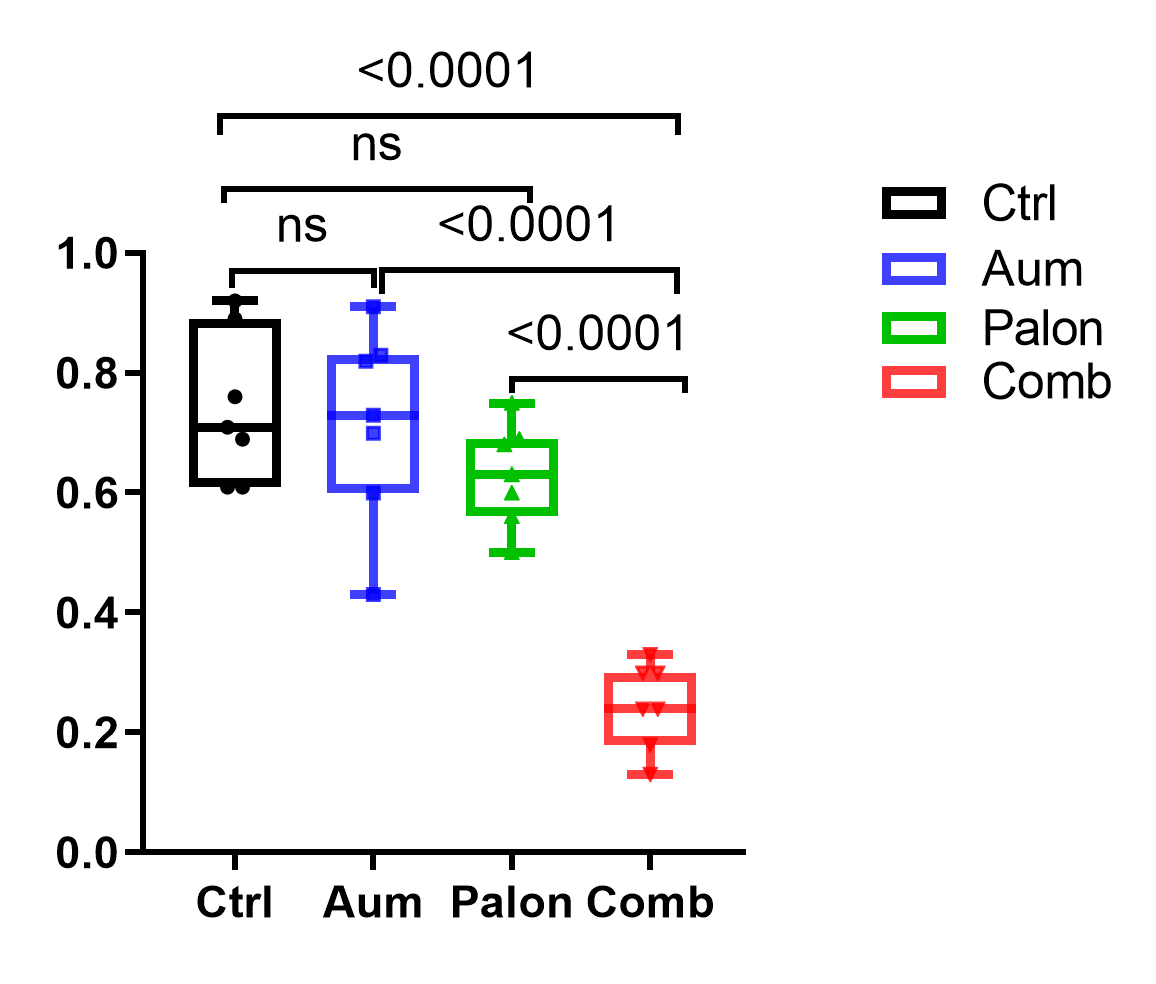

Supplement: Supplementary file 9 — Source data Fig. 6 [file 44321_2025_293_MOESM9_ESM.zip › Figure 6/6C/Tumor weight Fig 6C.tif]

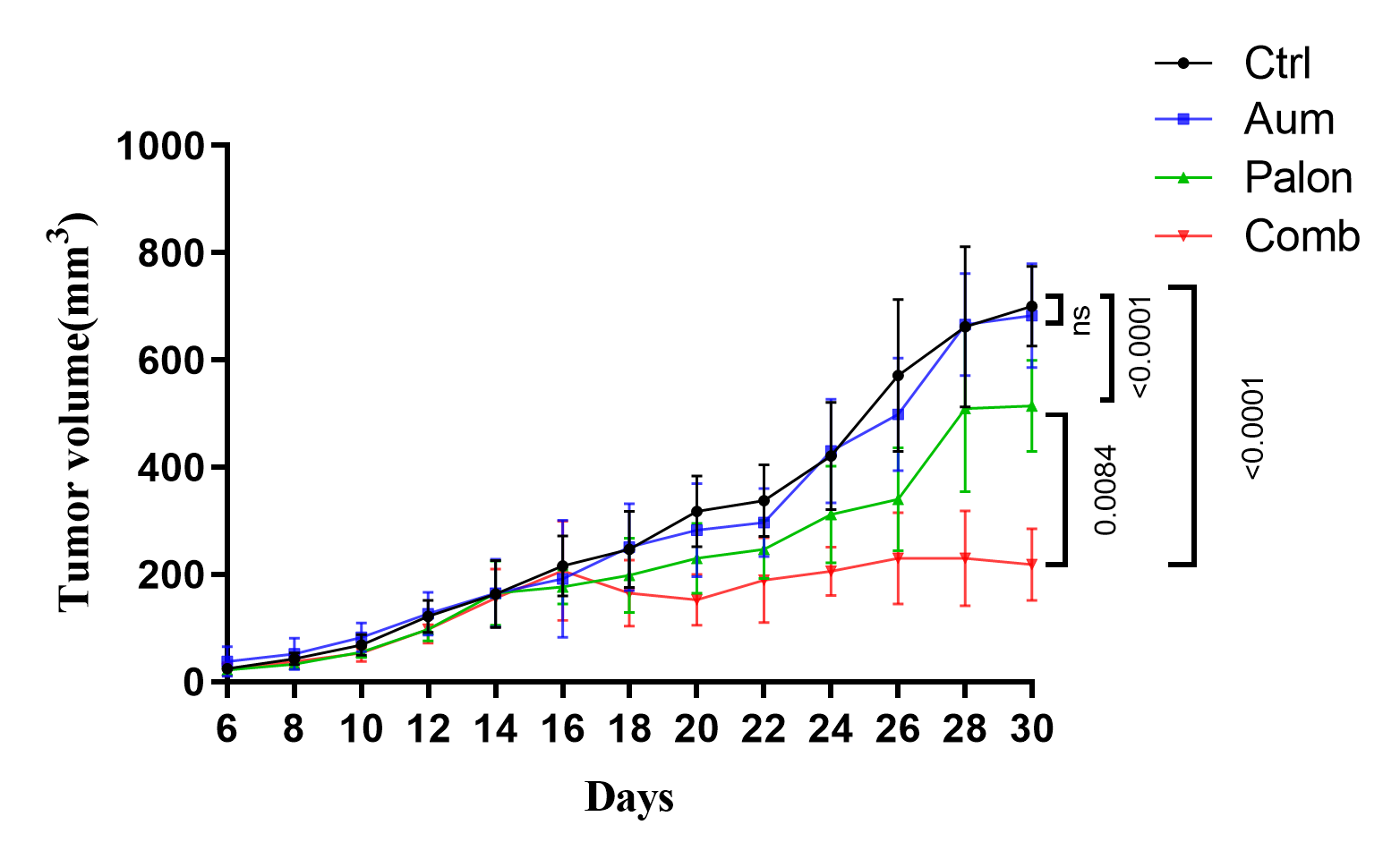

Supplement: Supplementary file 9 — Source data Fig. 6 [file 44321_2025_293_MOESM9_ESM.zip › Figure 6/6D/Fig 6D.tif]

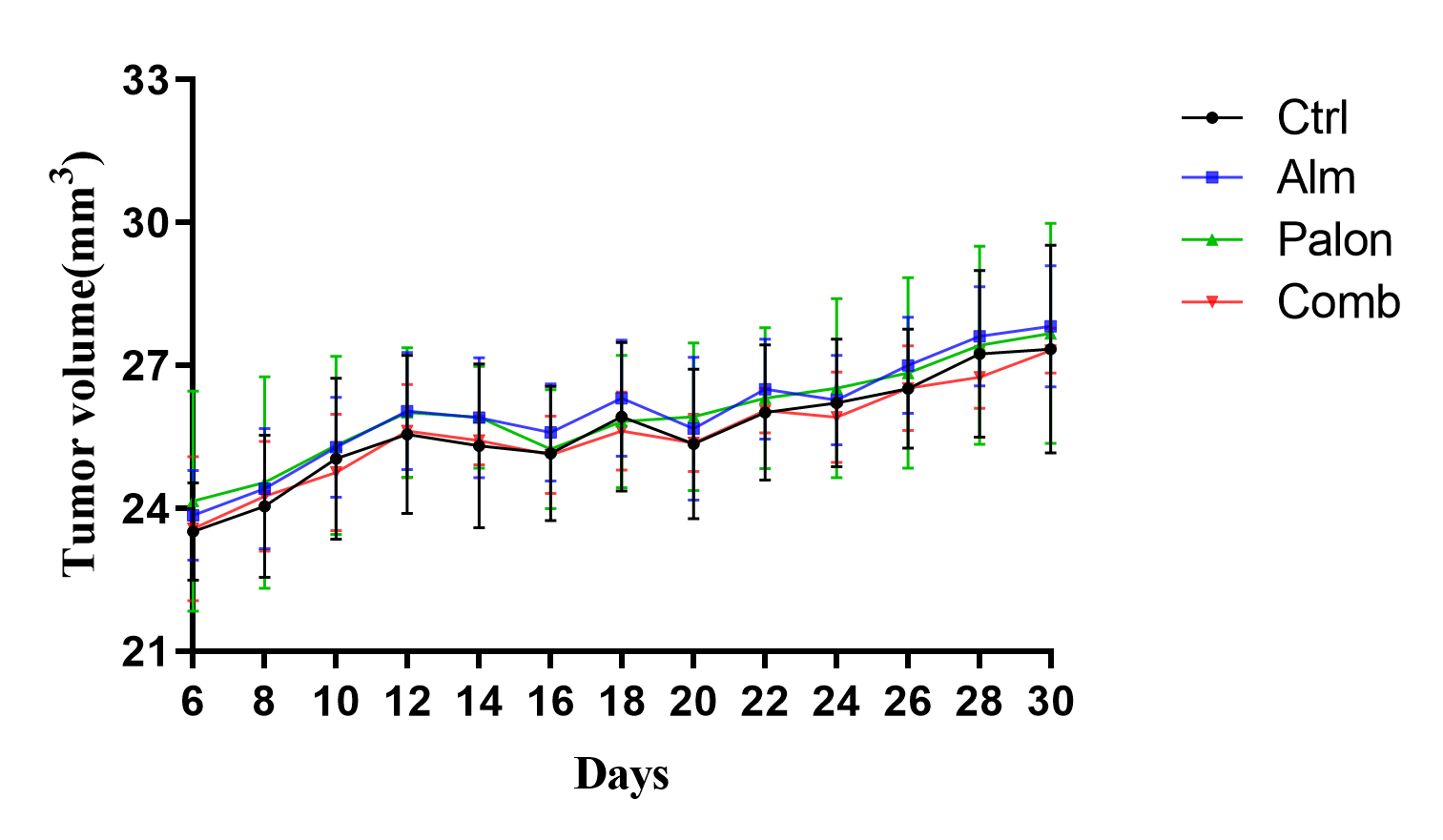

Supplement: Supplementary file 9 — Source data Fig. 6 [file 44321_2025_293_MOESM9_ESM.zip › Figure 6/6E/Body weight.tif]

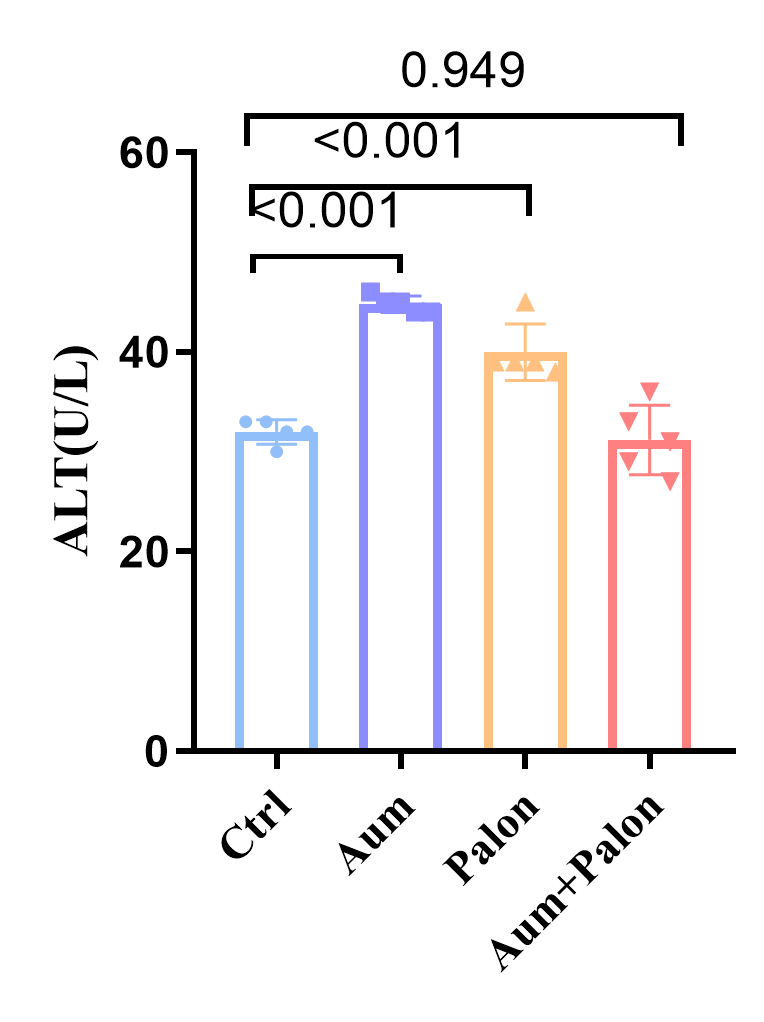

Supplement: Supplementary file 9 — Source data Fig. 6 [file 44321_2025_293_MOESM9_ESM.zip › Figure 6/6F-6I/ALT.tif]

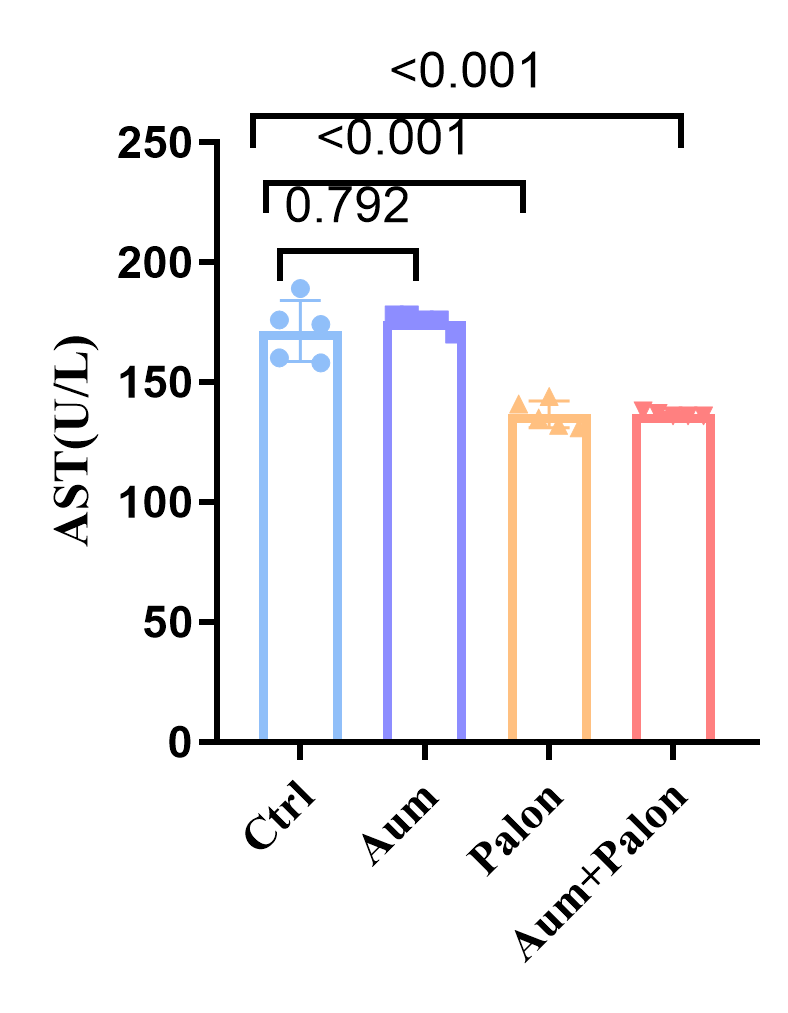

Supplement: Supplementary file 9 — Source data Fig. 6 [file 44321_2025_293_MOESM9_ESM.zip › Figure 6/6F-6I/AST.tif]

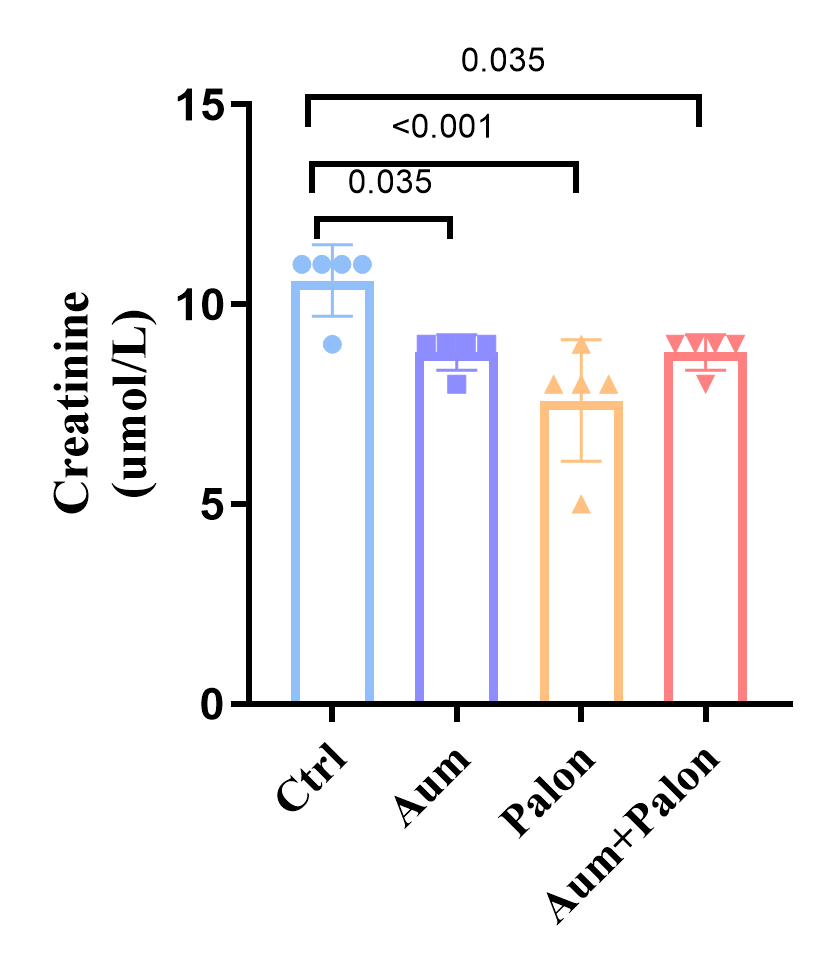

Supplement: Supplementary file 9 — Source data Fig. 6 [file 44321_2025_293_MOESM9_ESM.zip › Figure 6/6F-6I/Crea.tif]

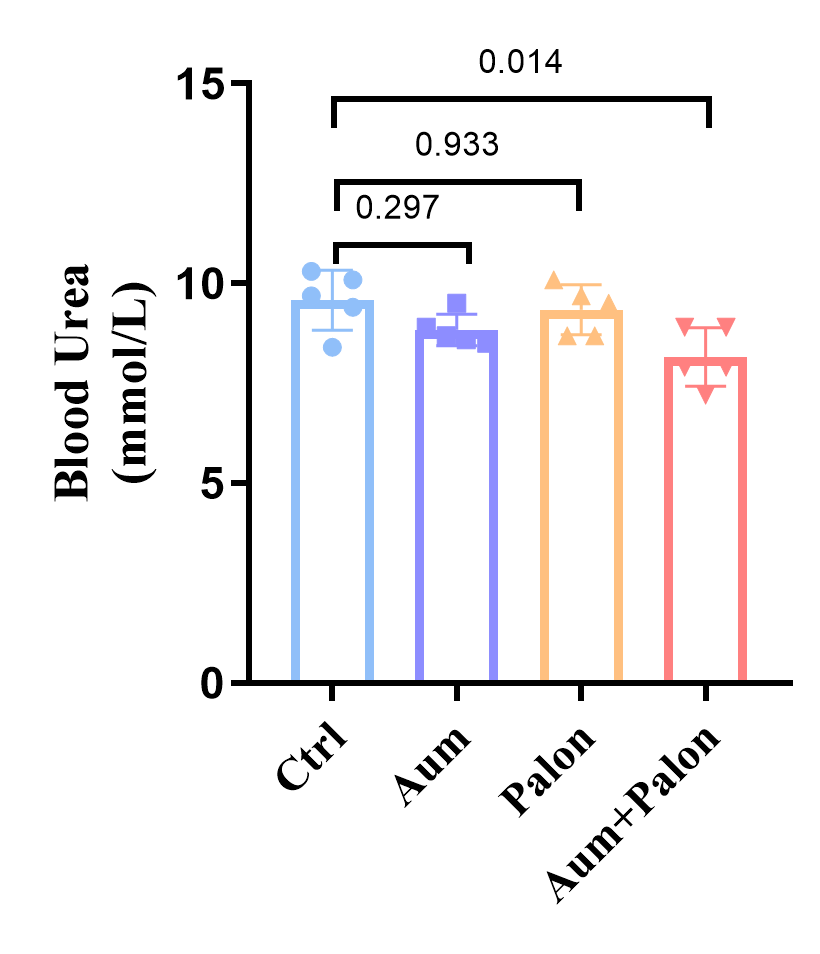

Supplement: Supplementary file 9 — Source data Fig. 6 [file 44321_2025_293_MOESM9_ESM.zip › Figure 6/6F-6I/Urea.tif]

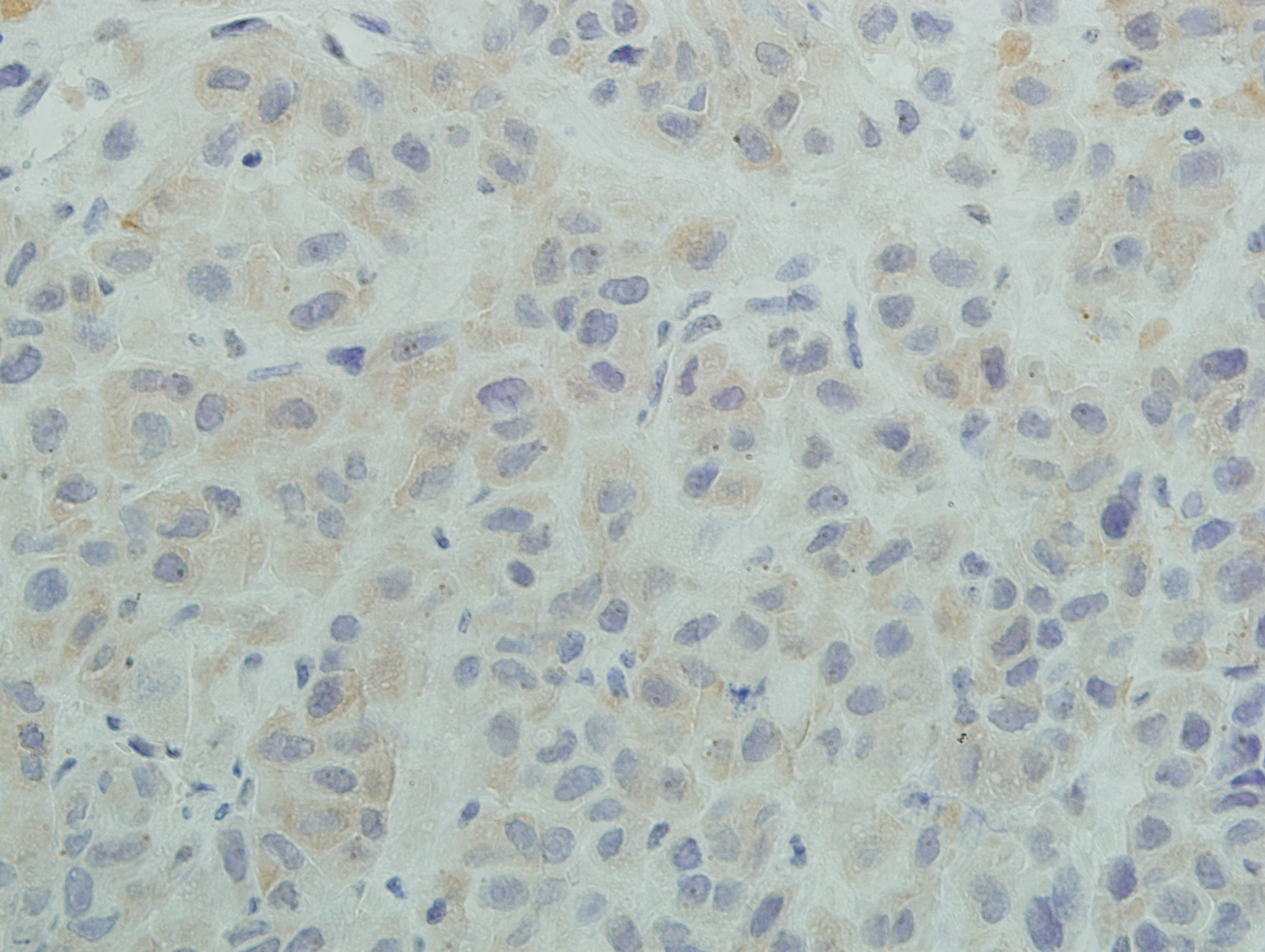

Supplement: Supplementary file 9 — Source data Fig. 6 [file 44321_2025_293_MOESM9_ESM.zip › Figure 6/6J/p-CAMKK2_Aum.tif]

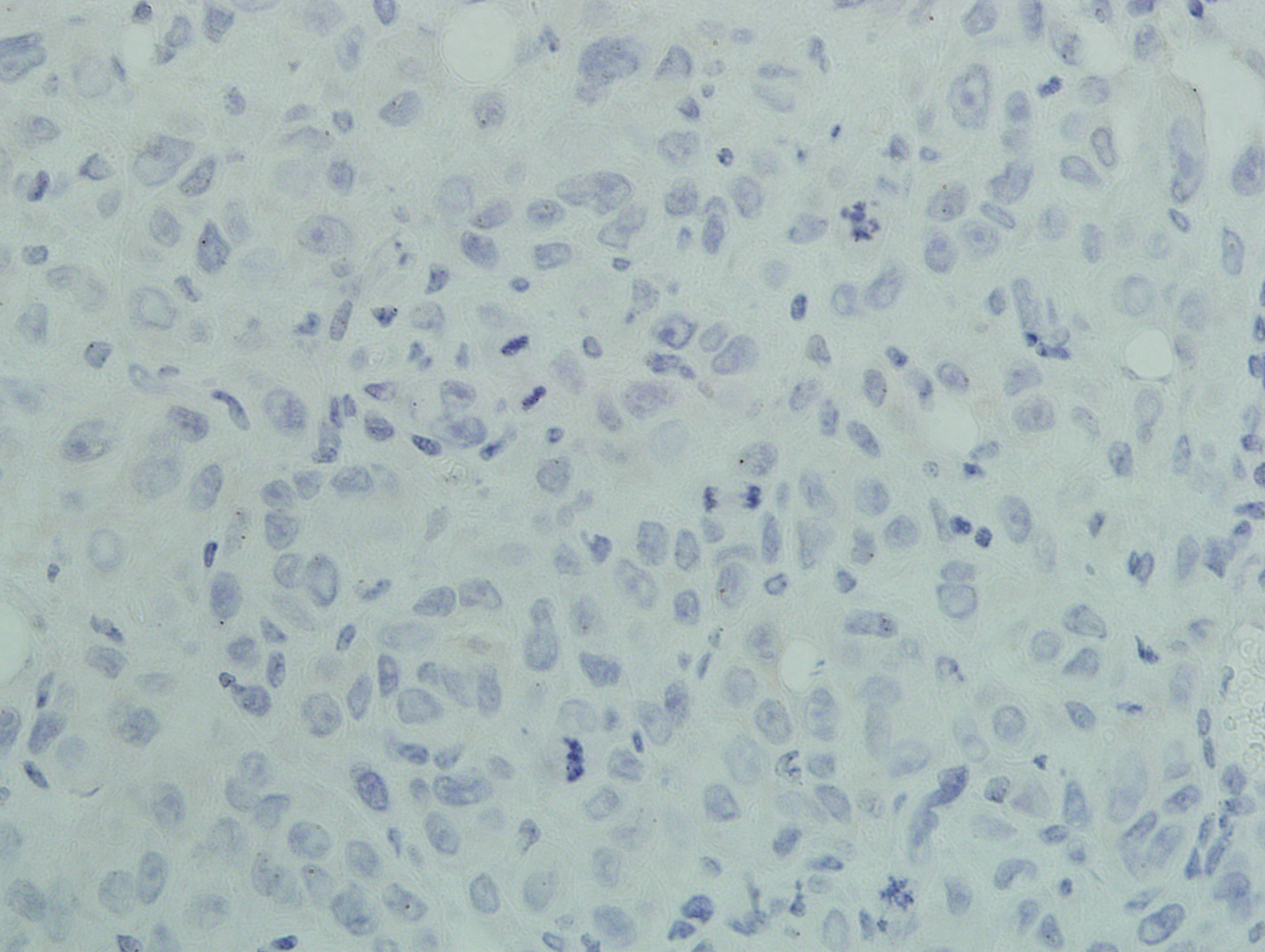

Supplement: Supplementary file 9 — Source data Fig. 6 [file 44321_2025_293_MOESM9_ESM.zip › Figure 6/6J/p-CAMKK2_Comb.tif]

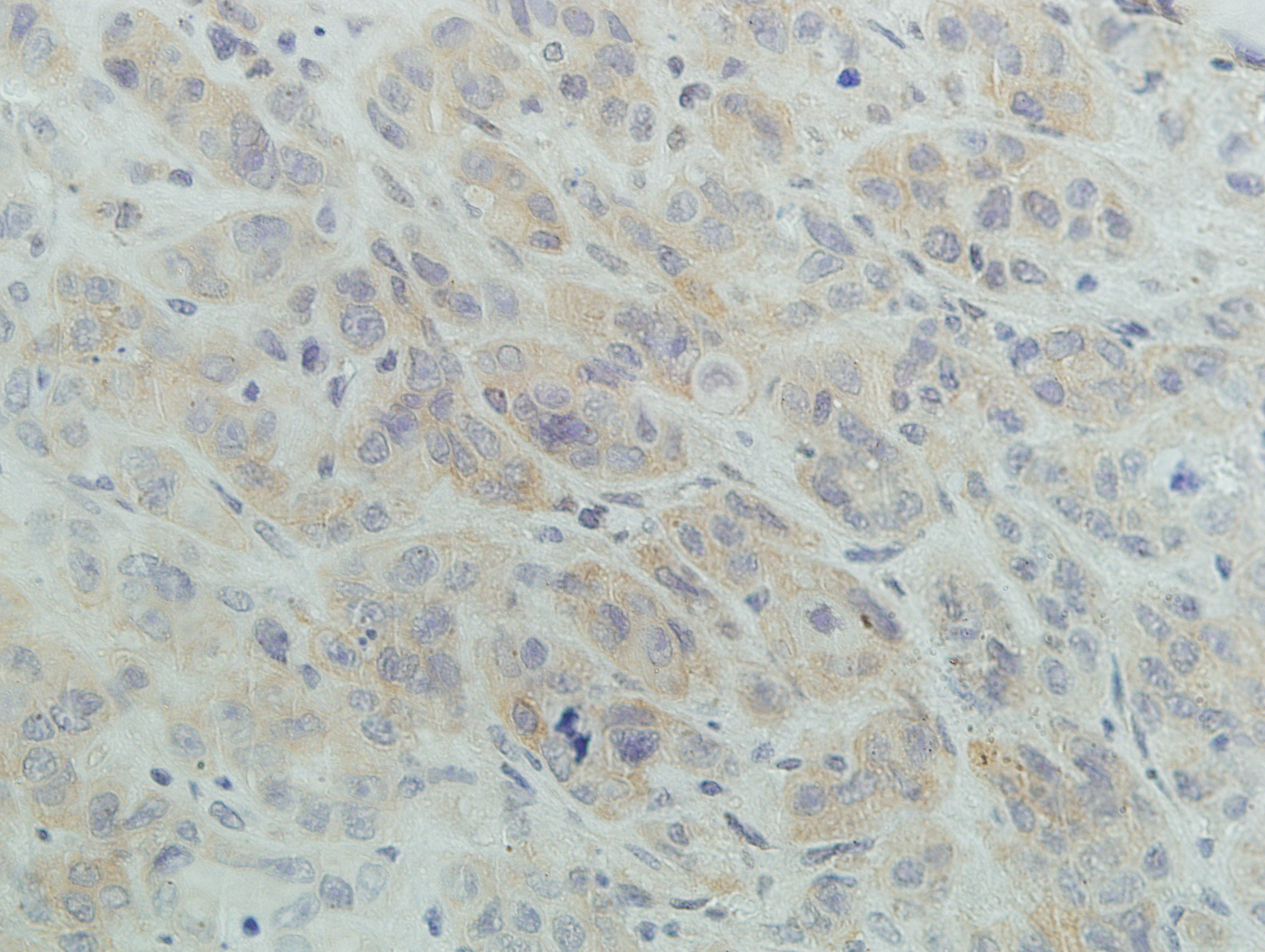

Supplement: Supplementary file 9 — Source data Fig. 6 [file 44321_2025_293_MOESM9_ESM.zip › Figure 6/6J/p-CAMKK2_Ctrl.tif]

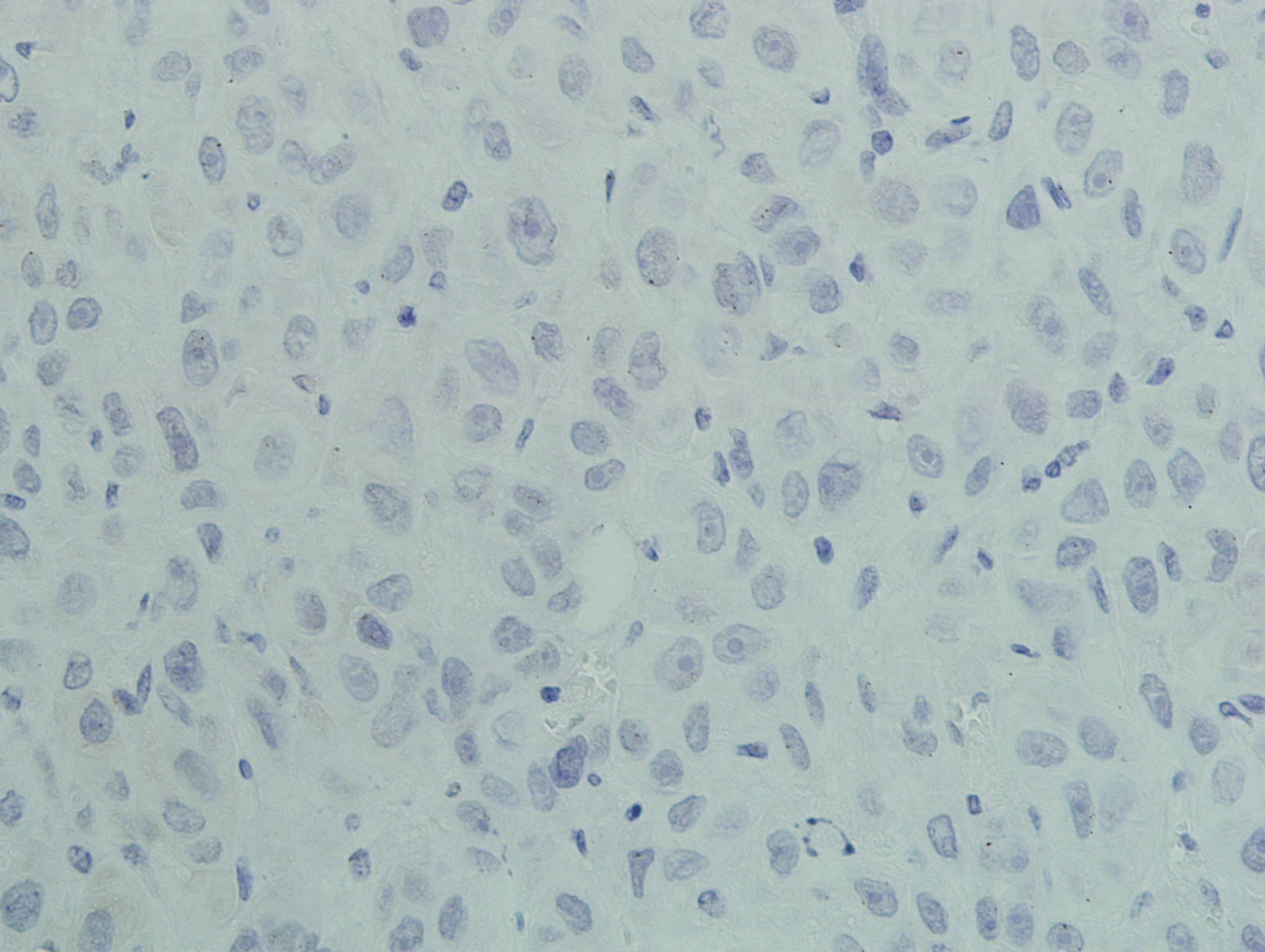

Supplement: Supplementary file 9 — Source data Fig. 6 [file 44321_2025_293_MOESM9_ESM.zip › Figure 6/6J/p-CAMKK2_Palon.tif]

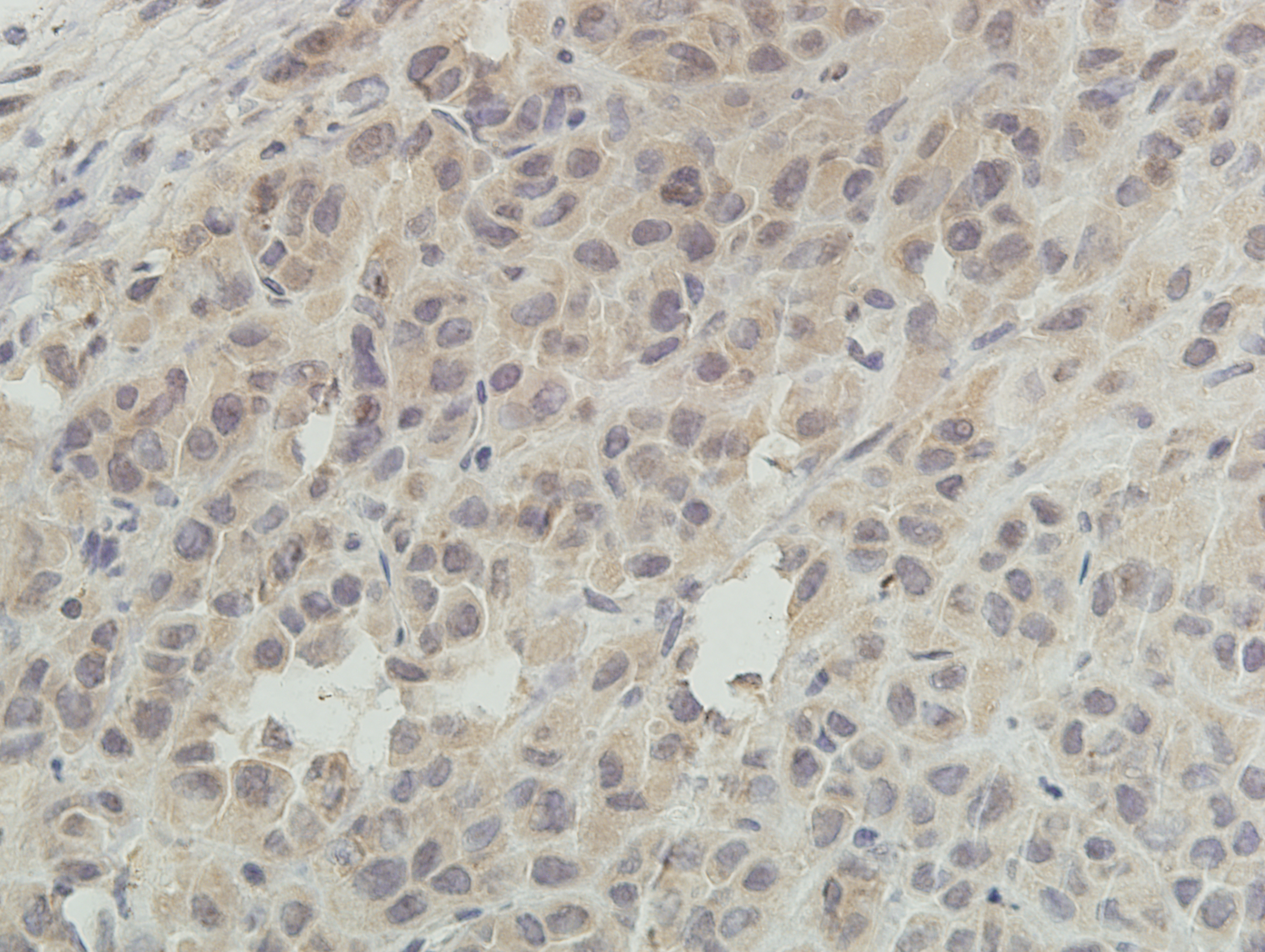

Supplement: Supplementary file 9 — Source data Fig. 6 [file 44321_2025_293_MOESM9_ESM.zip › Figure 6/6J/xCT-Aum.tif]

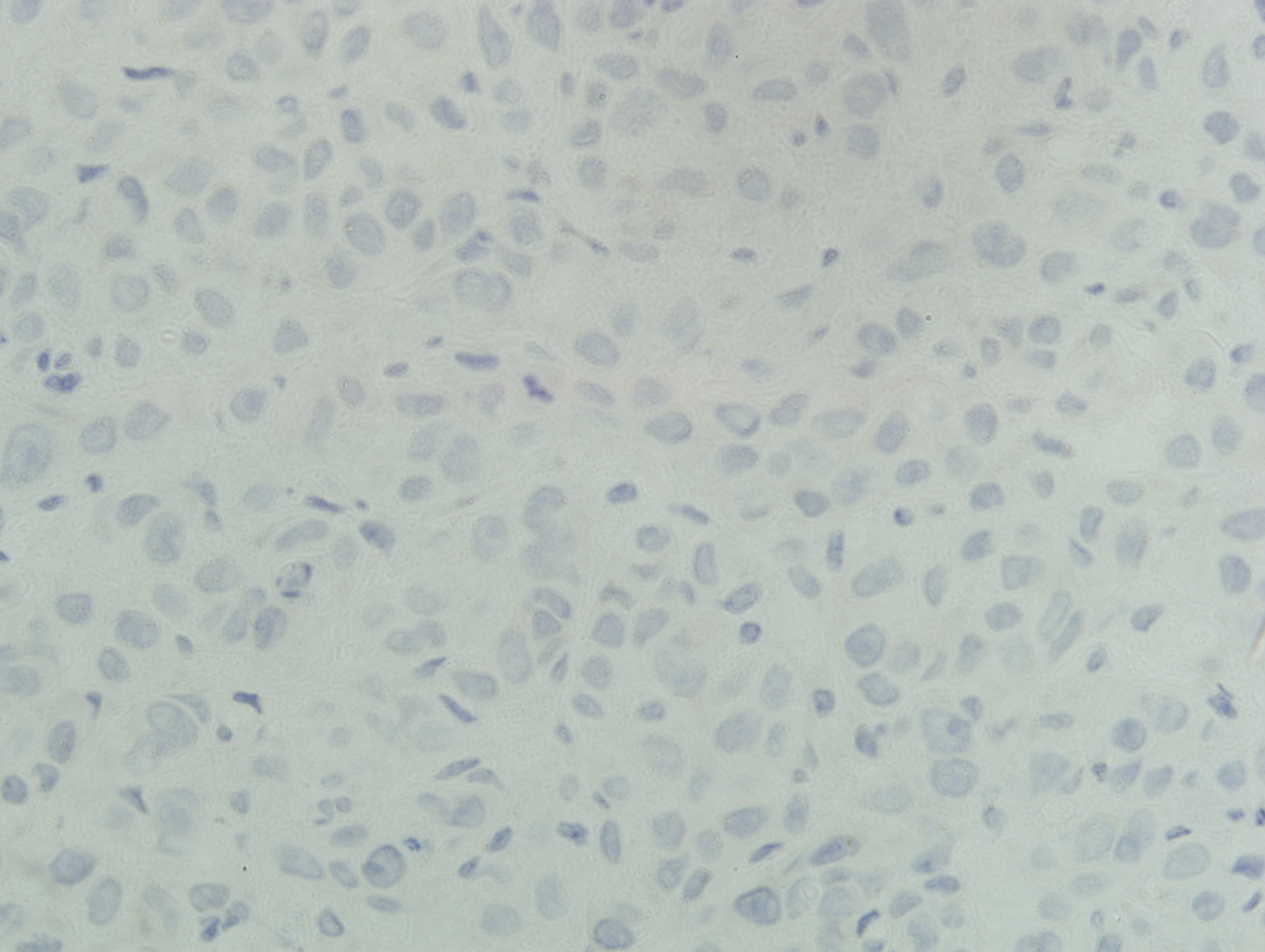

Supplement: Supplementary file 9 — Source data Fig. 6 [file 44321_2025_293_MOESM9_ESM.zip › Figure 6/6J/xCT_Comb.tif]

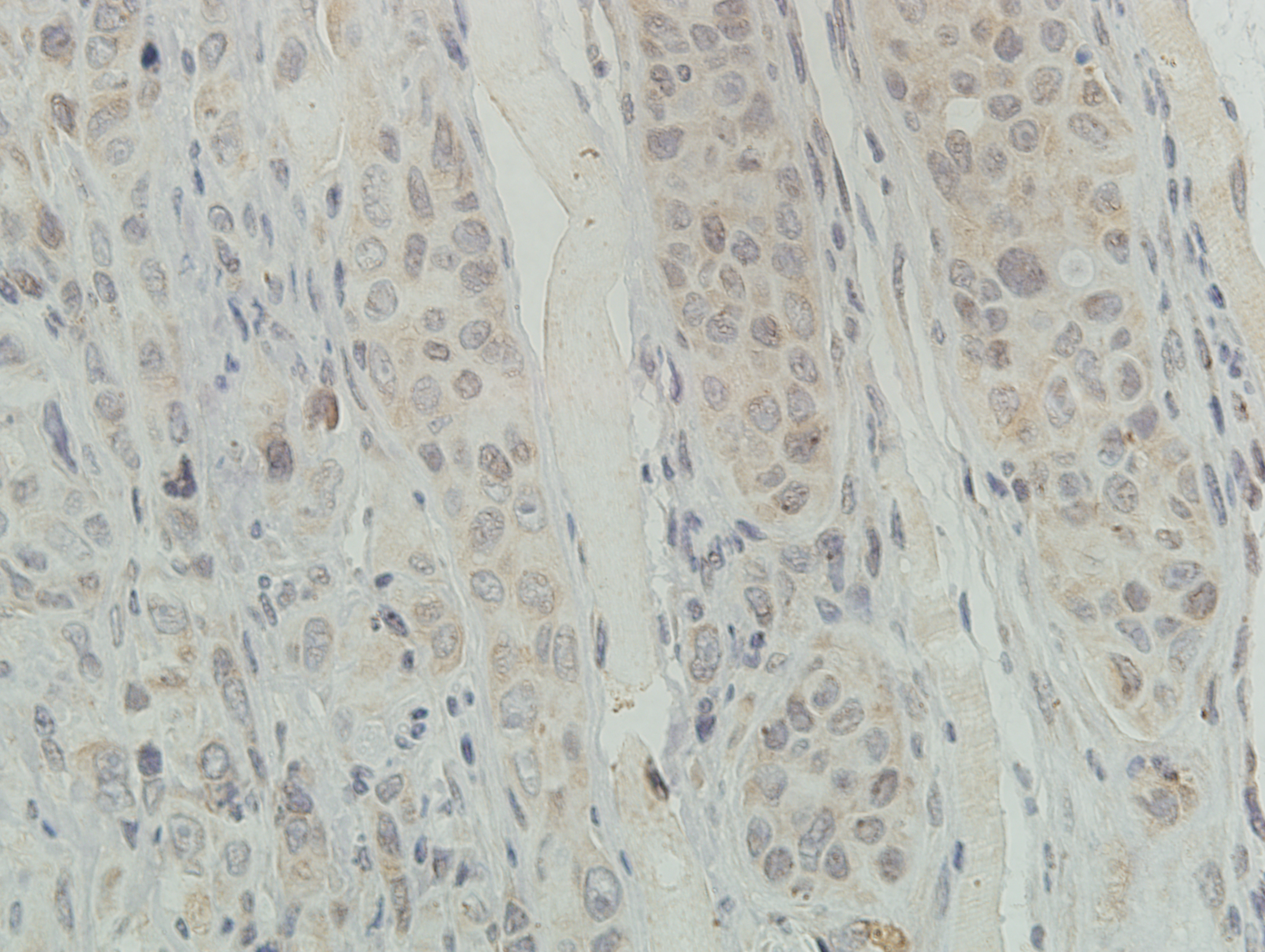

Supplement: Supplementary file 9 — Source data Fig. 6 [file 44321_2025_293_MOESM9_ESM.zip › Figure 6/6J/xCT_Ctrl.tif]

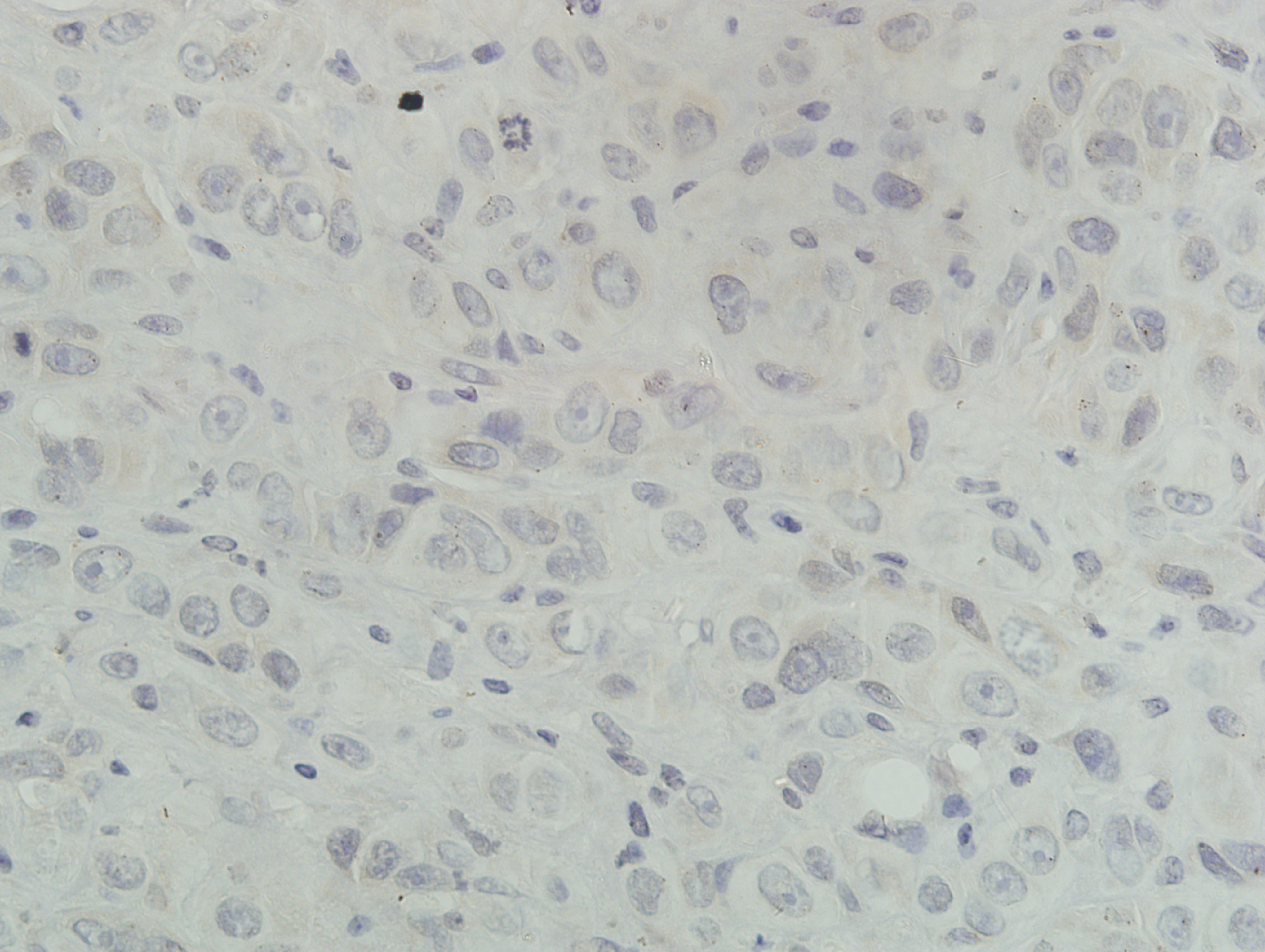

Supplement: Supplementary file 9 — Source data Fig. 6 [file 44321_2025_293_MOESM9_ESM.zip › Figure 6/6J/xCT_Palon.tif]
