# Supplementary material for: 5-HT regulates resistance to aumolertinib by attenuating ferroptosis in lung adenocarcinoma
Source: EMBO Mol Med. 2025 Sep 2;17(10):2586–611. doi: 10.1038/s44321-025-00293-5 (PMC12514003; doi:10.1038/s44321-025-00293-5)
Supplement: Supplementary file 10 — Expanded View Figures [file 44321_2025_293_MOESM10_ESM.pdf]

Expanded View Figures

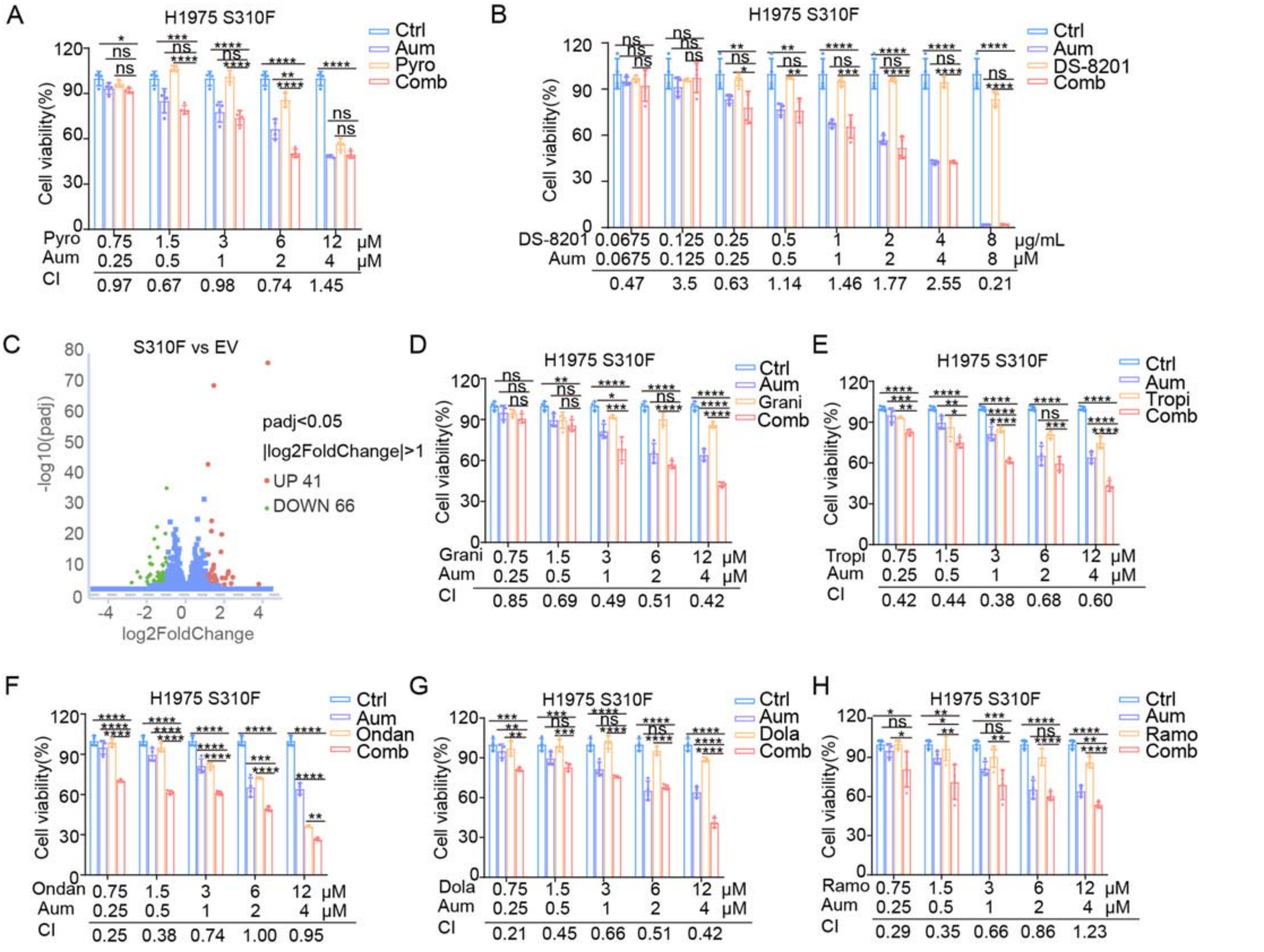

 © The Author(s)

Figure EV1. Therapeutic efficacy of aumolertinib combined with HER2-targeted agents and HTR3 antagonists.

(**A**) Cell viability and CI analysis in H1975 S310F cells treated with indicated concentrations of aumolertinib and pyrotinib alone or in combination for 72 h ($n = 4$). P value (Aum 0.25 µM, Pyro 0.75 µM): Ctrl vs Comb, $P = 0.0204$; P value (Aum 0.5 µM, Pyro 1.5 µM): Ctrl vs Comb, $P = 0.0004$, Pyro vs Comb, $P < 0.0001$; P value (Aum 1 µM, Pyro 3 µM): Ctrl vs Comb, $P < 0.0001$, Pyro vs Comb, $P < 0.0001$; P value (Aum 2 µM, Pyro 6 µM): Ctrl vs Comb, $P < 0.0001$, Aum vs Comb, $P = 0.0037$, Pyro vs Comb, $P = 0.0001$; P value (Aum 4 µM, Pyro 12 µM): Ctrl vs Comb, $P < 0.0001$. (**B**) Cell viability and CI analysis in H1975 S310F cells treated with indicated concentrations of aumolertinib and DS-8201 alone or in combination for 72 h ($n = 4$). P value (Aum 0.25 µM, DS-8201 0.25 µg/ml): Ctrl vs Comb, $P = 0.007$, DS-8201 vs Comb, $P = 0.0239$; P value (Aum 0.5 µM, DS-8201 0.5 µg/ml): Ctrl vs Comb, $P = 0.0011$, DS-8201 vs Comb, $P = 0.003$; P value (Aum 1 µM, DS-8201 1 µg/ml): Ctrl vs Comb, $P < 0.0001$, DS-8201 vs Comb, $P = 0.0002$; P value (Aum 2 µM, DS-8201 2 µg/ml): Ctrl vs Comb, $P < 0.0001$, DS-8201 vs Comb, $P < 0.0001$; P value (Aum 4 µM, DS-8201 4 µg/ml): Ctrl vs Comb, $P < 0.0001$, DS-8201 vs Comb, $P < 0.0001$; P value (Aum 8 µM, DS-8201 8 µg/ml): Ctrl vs Comb, $P < 0.0001$, DS-8201 vs Comb, $P < 0.0001$. (**C**) Differential gene expression volcano plot of H1975 EV/S310F transcriptome sequencing. (**D**) Cell viability and CI analysis in H1975 S310F cells treated with indicated concentrations of aumolertinib and granisetron alone or in combination for 72 h ($n = 4$). P value (Aum 0.5 µM, Grani 1.5 µM): Ctrl vs Comb, $P = 0.0050$; P value (Aum 1 µM, Grani 3 µM): Ctrl vs Comb, $P < 0.0001$, Aum vs Comb, $P = 0.0208$, Grani vs Comb, $P = 0.0002$; P value (Aum 2 µM, Grani 6 µM): Ctrl vs Comb, $P < 0.0001$, Grani vs Comb, $P < 0.0001$; P value (Aum 4 µM, Grani 12 µM): Ctrl vs Comb, $P < 0.0001$, Aum vs Comb, $P < 0.0001$, Grani vs Comb, $P < 0.0001$. (**E**) Cell viability and CI analysis in H1975 S310F cells treated with indicated concentrations of aumolertinib and tropisetron alone or in combination for 72 h ($n = 4$). P value (Aum 0.25 µM, Tropi 0.75 µM): Ctrl vs Comb, $P < 0.0001$, Aum vs Comb, $P = 0.0003$, Tropi vs Comb, $P = 0.0012$; P value (Aum 0.5 µM, Tropi 1.5 µM): Ctrl vs Comb, $P < 0.0001$, Aum vs Comb, $P = 0.0050$, Tropi vs Comb, $P = 0.0270$; P value (Aum 1 µM, Tropi 3 µM): Ctrl vs Comb, $P < 0.0001$, Aum vs Comb, $P < 0.0001$, Tropi vs Comb, $P < 0.0001$; P value (Aum 2 µM, Tropi 6 µM): Ctrl vs Comb, $P < 0.0001$, Tropi vs Comb, $P = 0.0001$; P value (Aum 4 µM, Tropi 12 µM): Ctrl vs Comb, $P < 0.0001$, Aum vs Comb, $P < 0.0001$, Tropi vs Comb, $P < 0.0001$. (**F**) Cell viability and CI analysis in H1975 S310F cells treated with indicated concentrations of aumolertinib and ondansetron alone or in combination for 72 h ($n = 4$). P value (Aum 0.25 µM, Ondan 0.75 µM): Ctrl vs Comb, $P < 0.0001$, Aum vs Comb, $P < 0.0001$, Ondan vs Comb, $P < 0.0001$; P value (Aum 0.5 µM, Ondan 1.5 µM): Ctrl vs Comb, $P < 0.0001$, Aum vs Comb, $P < 0.0001$, Ondan vs Comb, $P < 0.0001$; P value (Aum 1 µM, Ondan 3 µM): Ctrl vs Comb, $P < 0.0001$, Aum vs Comb, $P < 0.0001$, Ondan vs Comb, $P < 0.0001$; P value (Aum 2 µM, Ondan 6 µM): Ctrl vs Comb, $P < 0.0001$, Aum vs Comb, $P = 0.0006$, Ondan vs Comb, $P < 0.0001$; P value (Aum 4 µM, Ondan 12 µM): Ctrl vs Comb, $P < 0.0001$, Aum vs Comb, $P < 0.0001$, Ondan vs Comb, $P = 0.0020$. (**G**) Cell viability and CI analysis in H1975 S310F cells treated with indicated concentrations of aumolertinib and dolasetron alone or in combination for 72 h ($n = 4$). P value (Aum 0.25 µM, Dola 0.75 µM): Ctrl vs Comb, $P = 0.0003$, Aum vs Comb, $P = 0.0039$, Dola vs Comb, $P = 0.0015$; P value (Aum 0.5 µM, Dola 1.5 µM): Ctrl vs Comb, $P = 0.0007$, Dola vs Comb, $P = 0.0009$; P value (Aum 1 µM, Dola 3 µM): Ctrl vs Comb, $P < 0.0001$, Dola vs Comb, $P < 0.0001$; P value (Aum 2 µM, Dola 6 µM): Ctrl vs Comb, $P < 0.0001$, Dola vs Comb, $P < 0.0001$; P value (Aum 4 µM, Dola 12 µM): Ctrl vs Comb, $P < 0.0001$, Aum vs Comb, $P < 0.0001$, Dola vs Comb, $P < 0.0001$. (**H**) Cell viability and CI analysis in H1975 S310F cells treated with indicated concentrations of aumolertinib and ramosetron alone or in combination for 72 h ($n = 4$). P value (Aum 0.25 µM, Ramo 0.75 µM): Ctrl vs Comb, $P = 0.0179$, Ramo vs Comb, $P = 0.0174$; P value (Aum 0.5 µM, Ramo 1.5 µM): Ctrl vs Comb, $P = 0.0023$, Aum vs Comb, $P = 0.0440$, Ramo vs Comb, $P = 0.0059$; P value (Aum 1 µM, Ramo 3 µM): Ctrl vs Comb, $P = 0.0004$, Ramo vs Comb, $P = 0.0082$; P value (Aum 2 µM, Ramo 6 µM): Ctrl vs Comb, $P < 0.0001$, Ramo vs Comb, $P < 0.0001$; P value (Aum 4 µM, Ramo 12 µM): Ctrl vs Comb, $P < 0.0001$, Aum vs Comb, $P = 0.0074$, Ramo vs Comb, $P < 0.0001$. Data are presented as mean ± SD. Statistical test: one-way ANOVA, ns: not significant, $^*P < 0.05$, $^{**}P < 0.01$, $^{***}P < 0.001$, and $^{****}P < 0.0001$.

© The Author(s)

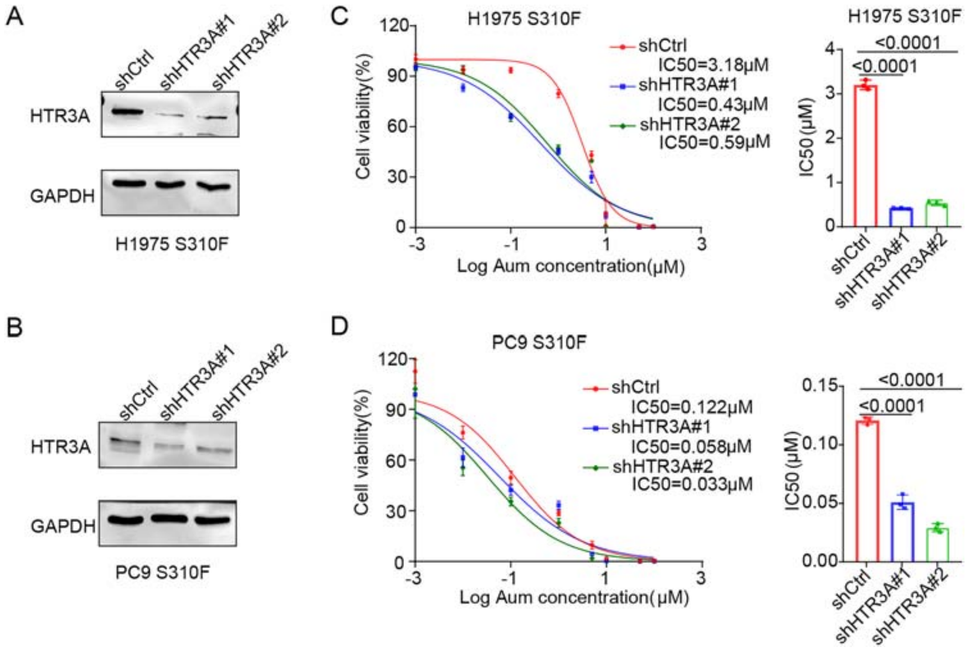

Figure EV2. HTR3A knockdown potentiates the sensitivity to aumolertinib in HER2 S310F-mutant cells.

(**A**, **B**) Western blot validation of stable HTR3A knockdown cell lines. (**C**, **D**) The aumolertinib IC_{50} value of H1975 S310F and PC9 S310F cells were calculated between shCtrl and shHTR3A groups after treating with aumolertinib at the indicated concentrations for 5 days ($n = 3$). Data are presented as mean ± SD. Statistical test: two-tailed unpaired Student's *t* test.

 © The Author(s)

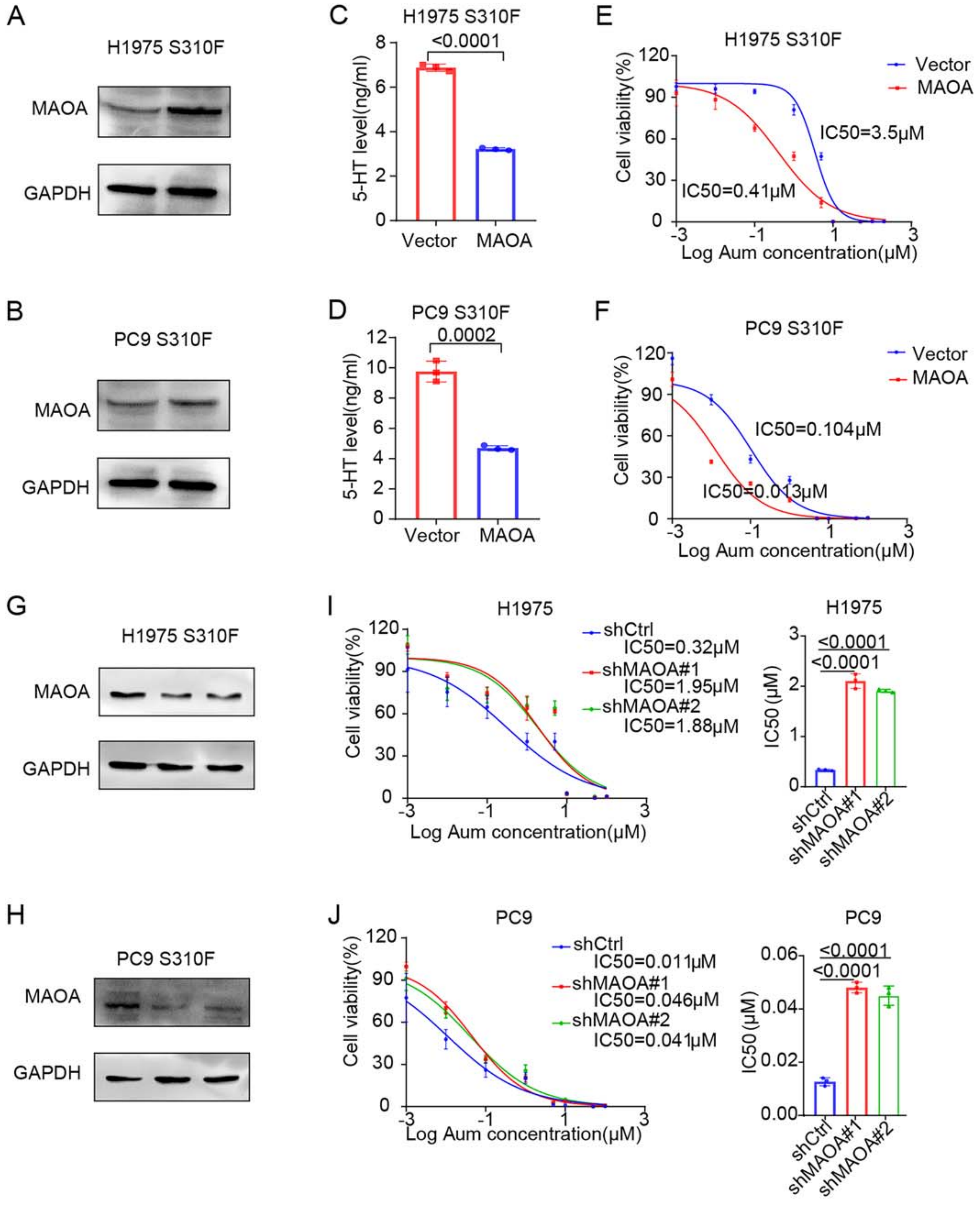
A
H1975 S310F
MAOA
GAPDH
B
PC9 S310F
MAOA
GAPDH
C
H1975 S310F
<0.0001
5-HT level(ng/ml)
Vector
MAOA
D
PC9 S310F
0.0002
5-HT level(ng/ml)
Vector
MAOA
E
H1975 S310F
Vector
MAOA
IC50=3.5μM
IC50=0.41μM
Cell viability(%)
Log Aum concentration(μM)
F
PC9 S310F
Vector
MAOA
IC50=0.104μM
IC50=0.013μM
Cell viability(%)
Log Aum concentration(μM)
G
H1975 S310F
MAOA
GAPDH
H
PC9 S310F
MAOA
GAPDH
I
H1975
shCtrl IC50=0.32μM
shMAOA#1 IC50=1.95μM
shMAOA#2 IC50=1.88μM
Cell viability(%)
Log Aum concentration(μM)
H1975
<0.0001
<0.0001
IC50 (μM)
shCtrl
shMAOA#1
shMAOA#2
J
PC9
shCtrl IC50=0.011μM
shMAOA#1 IC50=0.046μM
shMAOA#2 IC50=0.041μM
Cell viability(%)
Log Aum concentration(μM)
PC9
<0.0001
<0.0001
IC50 (μM)
shCtrl
shMAOA#1
shMAOA#2

Figure EV3. MAOA expression regulates aumolertinib resistance.

(**A**, **B**) H1975 S310F and PC9 S310F cells stably overexpressing MAOA were validated by western blot. (**C**, **D**) 5-HT levels in the supernatant ($n = 3$). (**E**, **F**) The aumolertinib IC_{50} value of H1975 S310F and PC9 S310F cells were calculated between vector and MAOA overexpression groups after treating with aumolertinib at the indicated concentrations for 5 days ($n = 4$). (**G**, **H**) Western blot validation of stable MAOA knockdown cell lines. (**I**, **J**) The aumolertinib IC_{50} value of H1975 and PC9 cells was calculated between shCtrl and shMAOA groups after treating with aumolertinib at the indicated concentrations for 5 days ($n = 3$). Data are presented as mean ± SD. Statistical test: Two-tailed unpaired Student's *t* test.

 © The Author(s)

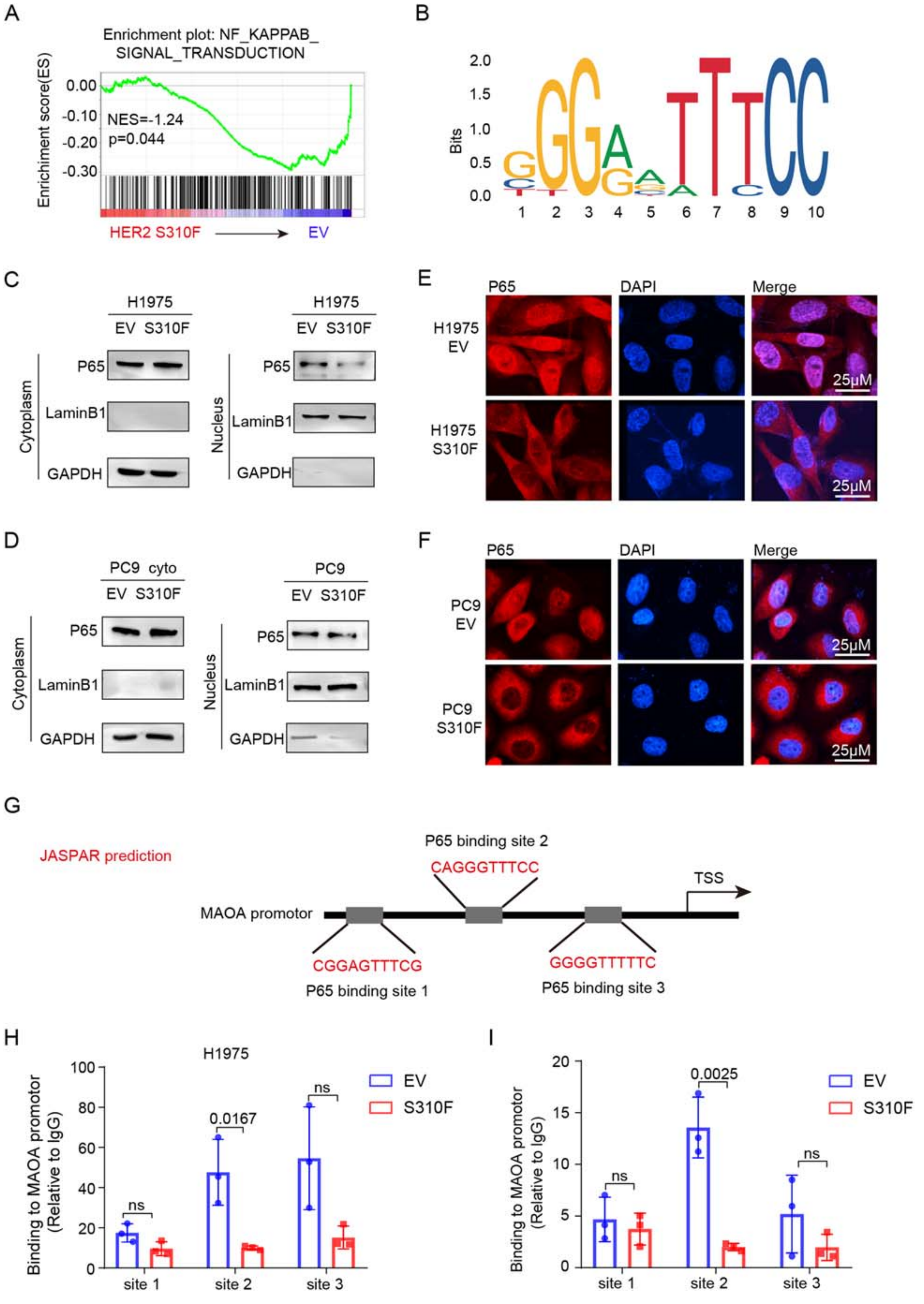
A
Enrichment plot: NF_KAPPAB_SIGNAL_TRANSDUCTION
Enrichment score(ES)
NES=-1.24
p=0.044
HER2 S310F
EV
B
Bits
C
H1975
EV S310F
P65
LaminB1
GAPDH
Cytoplasm
Nucleus
D
PC9 cyto
PC9
E
P65
DAPI
Merge
H1975 EV
H1975 S310F
25μM
F
PC9 EV
PC9 S310F
G
JASPAR prediction
P65 binding site 2
CAGGGTTTCC
TSS
MAOA promotor
CGGAGTTTCG
P65 binding site 1
GGGGTTTTTC
P65 binding site 3
H
H1975
Binding to MAOA promotor (Relative to IgG)
EV
S310F
ns
0.0167
site 1
site 2
site 3
I
0.0025

Figure EV4. The mechanism of downregulation of MAOA expression.

(**A**) Perform GSEA analysis on H1975 EV/S310F RNA seq data. (**B**) The JASPAR database forecasts the sequence of the P65 binding site within the promoter region of the MAOA gene. (**C**, **D**) The nuclear and cytoplasmic expression levels of P65 were assessed in H1975 EV/S310F and PC9 EV/S310F cells through nuclear–cytoplasmic separation experiments. (**E**, **F**) Immunofluorescence assays were conducted to determine the localization of P65 in H1975 EV/S310F and PC9 EV/S310F cells, with P65 appearing in red and the nuclei in blue. (**G**) The P65-binding motif was predicted by JASPAR, and schematic images of the potential P65 binding sites in the MAOA promoter region are shown. (**H**, **I**) ChIP-qPCR analysis of P65 binding on MAOA promoter in H1975 EV/S310F and PC9 EV/S310F cells ($n = 3$). Data are presented as mean ± SD. Statistical test: Two-tailed unpaired Student's *t* test.

 © The Author(s)
